# Multivalent designed proteins neutralize SARS-CoV-2 variants of concern and confer protection against infection in mice

**DOI:** 10.1126/scitranslmed.abn1252

**Published:** 2022-05-25

**Authors:** Andrew C. Hunt, James Brett Case, Young-Jun Park, Longxing Cao, Kejia Wu, Alexandra C. Walls, Zhuoming Liu, John E. Bowen, Hsien-Wei Yeh, Shally Saini, Louisa Helms, Yan Ting Zhao, Tien-Ying Hsiang, Tyler N. Starr, Inna Goreshnik, Lisa Kozodoy, Lauren Carter, Rashmi Ravichandran, Lydia B. Green, Wadim L. Matochko, Christy A. Thomson, Bastian Vögeli, Antje Krüger, Laura A. VanBlargan, Rita E. Chen, Baoling Ying, Adam L. Bailey, Natasha M. Kafai, Scott E. Boyken, Ajasja Ljubetič, Natasha Edman, George Ueda, Cameron M. Chow, Max Johnson, Amin Addetia, Mary Jane Navarro, Nuttada Panpradist, Michael Gale, Benjamin S. Freedman, Jesse D. Bloom, Hannele Ruohola-Baker, Sean P. J. Whelan, Lance Stewart, Michael S. Diamond, David Veesler, Michael C. Jewett, David Baker

**Affiliations:** 1 Department of Chemical and Biological Engineering, Northwestern University, Evanston, IL, 60208, USA; 2 Center for Synthetic Biology, Northwestern University, Evanston, IL, 60208, USA,; 3 Department of Medicine, Washington University School of Medicine, St. Louis, MO, 63110, USA; 4 Department of Biochemistry, University of Washington, Seattle, WA, 98195, USA; 5 Institute for Protein Design, University of Washington, Seattle, WA, 98195, USA; 6 Howard Hughes Medical Institute, University of Washington, Seattle, WA, 98195, USA; 7 Department of Molecular Microbiology, Washington University School of Medicine, St. Louis, MO, 63110, USA; 8 Institute for Stem Cell and Regenerative Medicine, University of Washington School of Medicine, Seattle, WA, 98109, USA; 9 Division of Nephrology, Department of Medicine, University of Washington School of Medicine, Seattle, WA, 98109, USA; 10 Kidney Research Institute, University of Washington School of Medicine, Seattle, WA, 98109, USA; 11 Oral Health Sciences, School of Dentistry, University of Washington, Seattle, WA, 98195, USA; 12 Department of Immunology, Center for Innate Immunity and Immune Disease, University of Washington, Seattle, WA, 98195, USA; 13 Basic Sciences Division, Fred Hutchinson Cancer Research Center, Seattle, WA, 98109, USA; 14 Amgen Research, Biologic Discovery, Burnaby, V5A 1V7, BC, Canada; 15 Invizyne Technologies Inc., Monrovia, CA, 91016, USA; 16 Department of Pathology & Immunology, Washington University School of Medicine, St. Louis, MO, 63110, USA; 17 Department of Pathology & Laboratory Medicine, University of Wisconsin – Madison, Madison, WI, 53705, USA; 18 Department for Synthetic Biology and Immunology, National Institute of Chemistry, Ljubljana, SI-1000, Slovenia; 19 Molecular and Cellular Biology Graduate Program, University of Washington, Seattle, WA, 98195, USA; 20 USA Medical Scientist Training Program, University of Washington, Seattle, WA, 98195, USA; 21 Neolukin Therapeutics Inc., Seattle, WA, 98102, USA; 22 The Molecular and Cellular Biology Program, University of Washington, Seattle, WA, 98195, USA; 23 Department of Bioengineering, University of Washington, Seattle, WA, 98195, USA; 24 Department of Laboratory Medicine and Pathology, University of Washington School of Medicine, Seattle, WA, 98109, USA; 25 Department of Genome Sciences, University of Washington, Seattle, WA, 98195, USA; 26 Andrew M. and Jane M. Bursky Center for Human Immunology and Immunotherapy Programs, Washington University School of Medicine, St. Louis, MO, 63110, USA; 27 Chemistry of Life Processes Institute, Northwestern University, Evanston, IL, 60208, USA; 28 Robert H. Lurie Comprehensive Cancer Center, Northwestern University, Chicago, IL, 60611, USA

## Abstract

New variants of severe acute respiratory syndrome coronavirus 2 (SARS-CoV-2) continue to arise and prolong the coronavirus disease 2019 (COVID-19) pandemic. Here we used a cell-free expression workflow to rapidly screen and optimize constructs containing multiple computationally designed miniprotein inhibitors of SARS-CoV-2. We found the broadest efficacy with a homo-trimeric version of the 75-residue angiotensin converting enzyme 2 (ACE2) mimic AHB2 (TRI2–2) designed to geometrically match the trimeric spike architecture. In the cryo-electron microscopy structure, TRI2 formed a tripod on top of the spike protein which engaged all three receptor binding domains (RBDs) simultaneously as in the design model. TRI2–2 neutralized Omicron (B.1.1.529), Delta (B.1.617.2), and all other variants tested with greater potency than that of monoclonal antibodies used clinically for the treatment of COVID-19. TRI2–2 also conferred prophylactic and therapeutic protection against SARS-CoV-2 challenge when administered intranasally in mice. Designed miniprotein receptor mimics geometrically arrayed to match pathogen receptor binding sites could be a widely applicable antiviral therapeutic strategy with advantages over antibodies and native receptor traps. By comparison, the designed proteins have resistance to viral escape and antigenic drift by construction, precisely tuned avidity, and greatly reduced chance of autoimmune responses.

## ONE SENTENCE SUMMARY

Computationally designed trivalent minibinders provide therapeutic protection in mice against emerging SARS-CoV-2 variants of concern.

## INTRODUCTION

Severe acute respiratory syndrome coronavirus 2 (SARS-CoV-2) continues to cause a global pandemic with more than 300 million infections and 5.5 million deaths as of January 2022 (https://covid19.who.int/). Monoclonal antibodies (mAbs) targeting the SARS-CoV-2 spike (S) glycoprotein (1) have been an effective treatment for improving outcomes for patients with coronavirus disease 2019 (COVID-19) (2–5), but many are sensitive to viral escape through point mutations in their epitopes on the S trimer (6, 7), and producing mAbs in sufficient quantities for population scale use during a global pandemic is technically and financially challenging (8). Indeed, the continued emergence of variants of concern (VOCs) jeopardizes the effectiveness of currently approved mAb treatments and vaccines (9–14). In particular mutations in the rapidly spreading B.1.1.529 (Omicron) variant disrupt binding of most receptor binding motif-targeted mAbs, and have been shown to reduce neutralization potency more than 100-fold for five of the seven clinical mAbs used for the prophylactic or therapeutic treatment of COVID-19 (15–18). Thus, there is an urgent need for interventions whose efficacy is not disrupted by the observed ongoing antigenic drift, as is the case for a few mAbs (19–24).

As an alternative to mAbs, we previously computationally designed two classes of minibinder proteins that block the SARS-CoV-2 receptor binding domain (RBD) interaction with its host receptor, angiotensin converting enzyme 2 (ACE2) (25). The first class, exemplified by AHB2, adopts a similar binding mode to and incorporates residues from the main RBD-interacting helix of ACE2 in a custom designed 3-helix bundle that has low overall sequence similarity with ACE2 (fig. S1). The second class, exemplified by LCB1 and LCB3, contain an entirely new designed RBD binding interface. These minibinders neutralize the WA1/2020 SARS-CoV-2 virus with half maximal inhibitory concentration (IC_50_) values in the range of 23 pM (LCB1) to 15 nM (AHB2) (25). The designs express at high concentrations in *Escherichia coli* and are highly thermostable (25), which could considerably streamline manufacturing and decrease the cost of goods for clinical development. LCB1 has demonstrated protective activity as both pre-exposure prophylaxis and post-exposure therapy in human ACE2 (hACE2)-expressing transgenic mice, but mutations in the B.1.351 (Beta) and P.1 (Gamma) VOCs were shown to reduce binding potency (26, 27).

Here, we sought to develop constructs containing three minibinder domains that could simultaneously engage all three RBDs on a single S protein, and by virtue of this multivalent binding, potently neutralize SARS-CoV-2 variants. Multivalency can increase the apparent affinity for target antigens (28–30), including against SARS-CoV-2 (31–36). We considered two classes of constructs. The first contain multiple distinct minibinder domains linked together to maximize RBD binding avidity; these constructs have the advantages that LCB1 and LCB3 are very high affinity binders on their own, and the three domains contain different sets of contacts with the RBD, making escape in principle more difficult (32, 37). The second consists of self-assembling homotrimers of minibinders geometrically matched to the 3 RBDs on a single spike; although AHB2 is lower affinity than LCB1 and LCB3, and the sites targeted are less diverse than the first class, homotrimers of AHB2 have the advantage that the ACE2 binding site is inherently less mutable as the virus must bind ACE2 to infect cells (24, 38). We describe the design, optimization, and escape resistance of both classes of constructs. We find that the top constructs have considerable promise as potential countermeasures in the ongoing COVID-19 pandemic.

## RESULTS

### RBD mutations impact minibinder binding.

To determine the potential for mutations to arise that disrupt LCB1 and AHB2 binding to the RBD, we performed deep mutational scans using site saturation mutagenesis of the RBD (38). We found that for LCB1, the widely observed K417N mutation results in a greater than 10-fold reduction in affinity and the E406W and Y453K/R mutations result in a greater than 100-fold reduction in affinity (fig. S2). For AHB2, we similarly observed several mutations, including K417N, E406W, and Y453K/R, that reduce the affinity of the minibinder for the RBD.

### Multivalent minibinders bind to SARS-CoV-2 RBDs.

To improve the ability of the minibinders to neutralize circulating SARS-CoV-2 variants, we developed multivalent versions with geometries enabling simultaneous engagement of all 3 RBDs in a single S trimer (1) to increase binding avidity. Multivalent minibinders might be less sensitive to mutations that would escape binding of the monovalent minibinders; a 100x reduction in binding affinity of a sub-picomolar binder would still result in an affinity in a therapeutic range in a multivalent construct (39). We also hypothesized that constructs with binding domains containing different sets of contacts with the target epitope could prevent escape (32, 37). To design multivalent constructs, we started from optimized versions of the previously described LCB1, AHB2, and LCB3 minibinders (hereafter referred to as monomers MON1, MON2, and MON3, respectively; table S1) (25).

To rapidly prototype multivalent minibinder designs, we developed a cell-free protein synthesis (CFPS) workflow which combines an in vitro DNA assembly step followed by polymerase chain reaction (PCR) to generate linear expression templates that are used to drive CFPS and enable rapid prototyping of new minibinder designs (fig. S3). The workflow enables assembly and translation of synthetic genes and generation of purified protein in as little as 6 hours, is compatible with high-throughput, automated experimentation using an acoustic liquid handler (Echo 525), and is easily scaled for the production of mg quantities of protein (40, 41). To assess multivalent binding, we coupled the workflow to an AlphaLISA protein-protein interaction (PPI) competition assay to enable comparison of dissociation rates of the designed proteins against either the monomeric RBD or the trimeric HexaPro SARS-CoV-2-S-glycoprotein (S6P) (42).

Because multivalency largely impacts dissociation rate constants of protein-protein interactions, we reasoned that an in-solution off-rate screen could distinguish differences between mono- and multivalent binding (43). Multivalent minibinders were allowed to fully associate with the target protein, then reactions were split in two and either 100-fold molar excess of untagged competitor (to prevent reassociation) or buffer was added. MON1, MON2, and MON3 target overlapping epitopes (25), and thus mono- or multivalent versions of these minibinders were selected as competitors. The ratio of the competitor to no-competitor condition measurements were calculated to determine the fraction of the complex dissociated (44).

**Fig 1.**
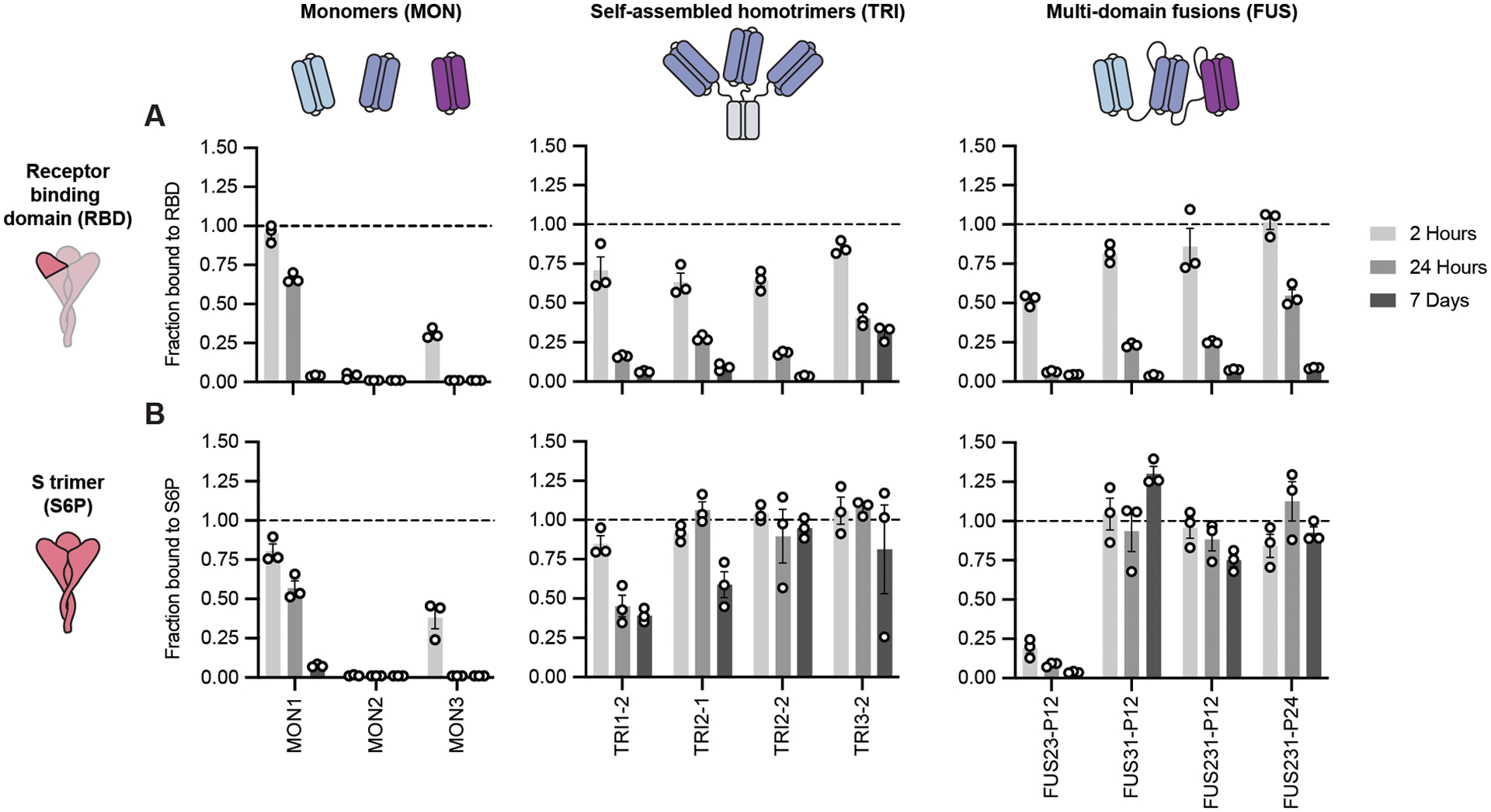
Multivalent minibinders exhibit very slow dissociation rates upon binding to the prefusion SARS-CoV-2-S glycoprotein trimer. Dissociation of the minibinder construct was monitored by competition with 100-fold molar excess of untagged MON1 using AlphaLISA (Mean ± SEM, n = 3 technical replicates from a single experiment). **(A)** Dissociation was measured for indicated minibinder constructs complexed with the receptor-binding domain of SARS-CoV-2 (RBD). **(B)** Dissociation was measured for the indicated minibinder constructs complexed with the S trimer (S6P).

Paralleling previous work where trimeric binders were targeted to the sialic acid-binding site on influenza hemagglutinin (30), we first designed self-assembling homotrimeric versions of the MON1, MON2, and MON3 miniproteins geometrically matched to the three RBDs in the S trimer (hereafter referred to as TRI; for example, TRI1–1 represents a homotrimer of MON1 with homotrimerization domain 1, table S1, data file S1). We designed and screened more than 100 different homotrimeric minibinders, with varied linker lengths and homotrimzeriation domains, using the CFPS workflow. We observed that many of the homotrimeric constructs exhibited slower dissociation rates than the corresponding monomers; much larger effects were observed with dissociation from the S trimer than monomeric RBD, consistent with multivalent binding (Fig. 1 and fig. S4). In total, we tested eleven different oligomerization domains and found that nine of these domains yielded at least one design with a linker length that improved dissociation rates on par with the top binders (fig. S4). Designs with domains four and eleven exhibited slower dissociation rates compared to their monomeric counterpart, but faster than the top designs (fig. S4E); this is likely indicative of an inability to simultaneously engage all three target epitopes or dissociation of the oligomerization domains themselves. The top binders exhibited little to no dissociation from S trimer after 7 days of incubation with competitor, indicating a likely apparent dissociation rate constant of 1×10^−7^ s^−1^ or slower (Fig. 1B). This is a marked improvement, more than four orders of magnitude for the TRI2 proteins, over the dissociation rate constants of the corresponding monomeric minibinders (fig. S5). We selected two trimeric scaffolds, the designed two ring helical bundle SB175 (domain 2) and the T4 foldon (domain 1) (45) (table S2), to proceed with based on the screening results and previous experience with these scaffolds.

Next, we generated two- and three-domain fusions of the MON1, MON2, and MON3 minibinders separated by flexible linkers (hereafter referred to as FUS; for example, FUS31-P12 represents a fusion of MON3 to MON1 separated by a 12 amino acid proline-alanine-serine (P12) linker, table S1, data file S1). We screened more than 100 different fusions using the CFPS workflow, evaluating different minibinder orderings and a range of linker compositions and lengths that span the distances between the termini of the domains when bound to the “open” and “closed” states of the RBD (Fig. 1, and fig. S4, A, B, and F) (1). We evaluated both glycine-serine (denoted as G) and proline-alanine-serine (denoted as P) linkers (46) and observed similar binding characteristics (Fig. 1 and fig. S4). We observed occasional truncation of the G linkers during expression and purification by *E. coli* proteases; however, this was less frequent for the P linkers than for the G linkers. FUS31 and FUS231 constructs showed slower dissociation against S6P than RBD, and exhibited slower dissociation than all monomeric minibinders tested, consistent with multivalent S6P engagement (Fig. 1). The top binders exhibited little dissociation from S6P after 7 days, indicating a likely apparent dissociation rate constant of 1×10^−7^ s^−1^ or slower, representing one order of magnitude or greater improvement over the corresponding monomeric minibinder dissociation rate constant (fig. S5). Finally, to determine the potential for low-cost purification by heat treatment, we recombinantly expressed MON1, FUS231-P12, and TRI2–2 in *E. coli*. The heat-treated soluble fraction was enriched with the expressed minibinder and contaminating background proteins were largely precipitated (fig. S6).

### Structural studies of minibinders in complex with SARS-CoV-2 S.

**Fig 2.**
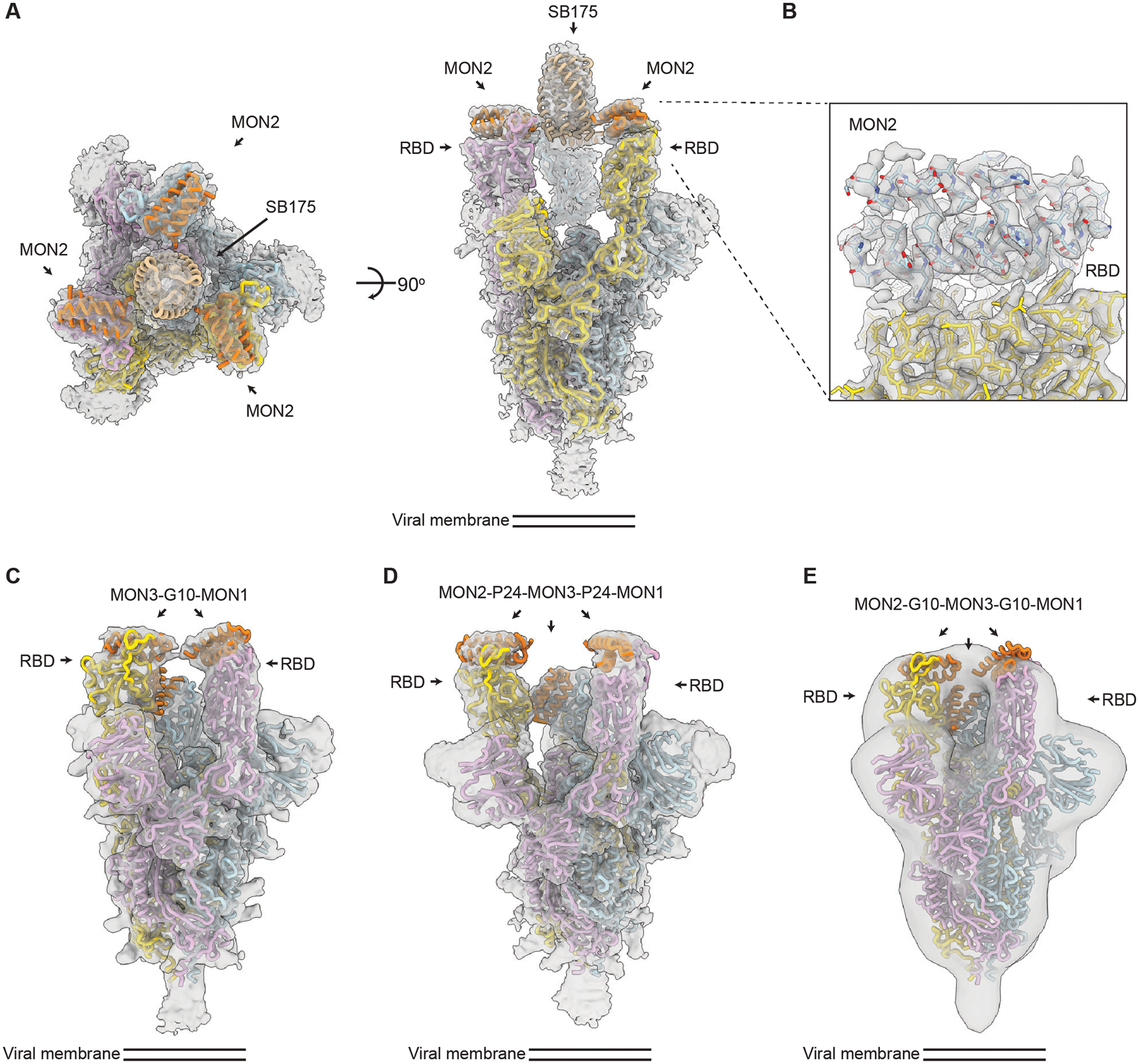
CryoEM structures of multivalent minibinders in complex with the SARS-CoV-2 S6P glycoprotein. TRI2–2 is a homotrimer of MON2 using the SB175 homotrimerization domain, FUS31-G10 is a fusion of MON3 to MON1 with a 10 amino acid glycine-serine linker, FUS213-P24 is a fusion of MON2 to MON1 to MON 3 with a 24 amino acid proline-alanine-serine linker, and FUS213-G10 is a fusion of MON2 to MON1 to MON 3 with a 10 amino acid glycine-serine linker. **(A)** A CryoEM map of TRI2–2 in complex with the S6P in two orthogonal orientations is shown. **(B)** A zoomed-in view of the TRI2–2 and RBD complex was obtained using focused 3D classification and local refinement. The RBD and MON2 built in the 2.9 Å resolution cryoEM map are shown in yellow and blue, respectively. **(C)** A cryoEM map of FUS31-G10 bound to S6P is shown. **(D)** A cryoEM map of FUS231-P24 bound to S6P is shown. **(E)** A negative-stain EM map of FUS231-G10 in complex with S6P is shown. S and minibinder models were docked in the whole map by rigid body fitting for visualization. In all panels, the EM density is shown as a transparent gray surface, S protomers (PDB 7JZL) are rendered in yellow, cyan, and pink and minibinders (PDB 7JZU, 7JZM, and MON2 structure was determined in this study) are shown in orange.

We next determined how the designed multivalent proteins engage multiple RBDs on a single S trimer; multivalent engagement on a virion typically requires binding of a single S trimer due to the relatively sparse S distribution (47–49). For some designs, FUS31-G8 and TRI1–5-G2 for example (table S1), initial screening using negative stain EM revealed considerable cross-linking and aggregation of S trimers upon addition of the constructs (fig. S7), consistent with binding to RBDs on different S trimers. In contrast, for constructs TRI2–2, FUS231-G10, FUS231-P24 and FUS31-G10, we observed less cross-linking, consistent with multivalent engagement of a single S trimer for each minibinder. To determine the binding modes of these compounds to the S trimer and characterize the structure of the MON2 and RBD interactions at high resolution, we carried out cryogenic electron microscopy (cryoEM) characterization of these complexes (Fig. 2).

The cryoEM structures of the TRI2–2, FUS31-G10, and the FUS231-P24 constructs in complex with S6P were determined at resolutions of 2.8, 4.6, and 3.9 Å respectively (Fig. 2A to D, fig. S8 to S11, and table S3), and a negative stain reconstruction was obtained with FUS231-G10 in complex with S6P (Fig. 2E). The TRI2–2/S6P cryoEM structure closely matched the TRI2–2 trimer design, with all three RBDs in the open state bound to MON2 (Fig. 2A and B, fig. S8 and S9). In the FUS31-G10 and S6P complex, FUS31-G10 is bound to two RBDs adopting an open conformation (Fig. 2C, fig. S8 and S10). The distance between the two RBDs in the open conformation is shorter in the FUS31-G10 than in the FUS231-P24 structure (Fig. 2C and D), suggesting that the bound minibinder holds the RBDs together, in agreement with the shorter linkers used in the former minibinder construct. In the structure, two molecules of FUS31-G10 are bound to a single S trimer with the third RBD being occupied by a second FUS31-G10 molecule. In the structure of FUS231-P24 bound to S6P, the three RBDs are participating in complex formation (Fig. 2D, fig. S8 and S11). The limited resolution in the region comprising the minibinder-bound RBDs and linkers precludes definitive assignment of minibinder identity at each binding site and relative connectivity between each minibinder module. The distances between the termini of the minibinder domains, however, is compatible with the computational design models and suggestive of engagement of either 2 (FUS31-G10) or 3 of the RBDs (FUS231-P24) in a single S trimer by the multivalent minibinders.

The structure of MON2 in complex with the S trimer has not previously been determined. Starting from the TRI2–2/S6P cryoEM data, we improved the RBD/MON2 densities using focused classification and local refinement, yielding a map at 2.9 Å resolution enabling visualization of the interactions formed by MON2 with the RBD (Fig. 2B). Superimposition of the design MON2 model to the corresponding cryoEM structure, using the RBD as reference, shows that the MON2 minibinder closely matched the design model with backbone Cɑ RMSD of 1.3 Å (fig. S8E and F). Together with previous structures of MON1 and MON3 (25), these data illustrate the accuracy with which both protein scaffolds and binding interfaces can now be computationally designed.

### Multivalent minibinders enable rapid detection of SARS-CoV-2 S protein.

**Fig 3.**
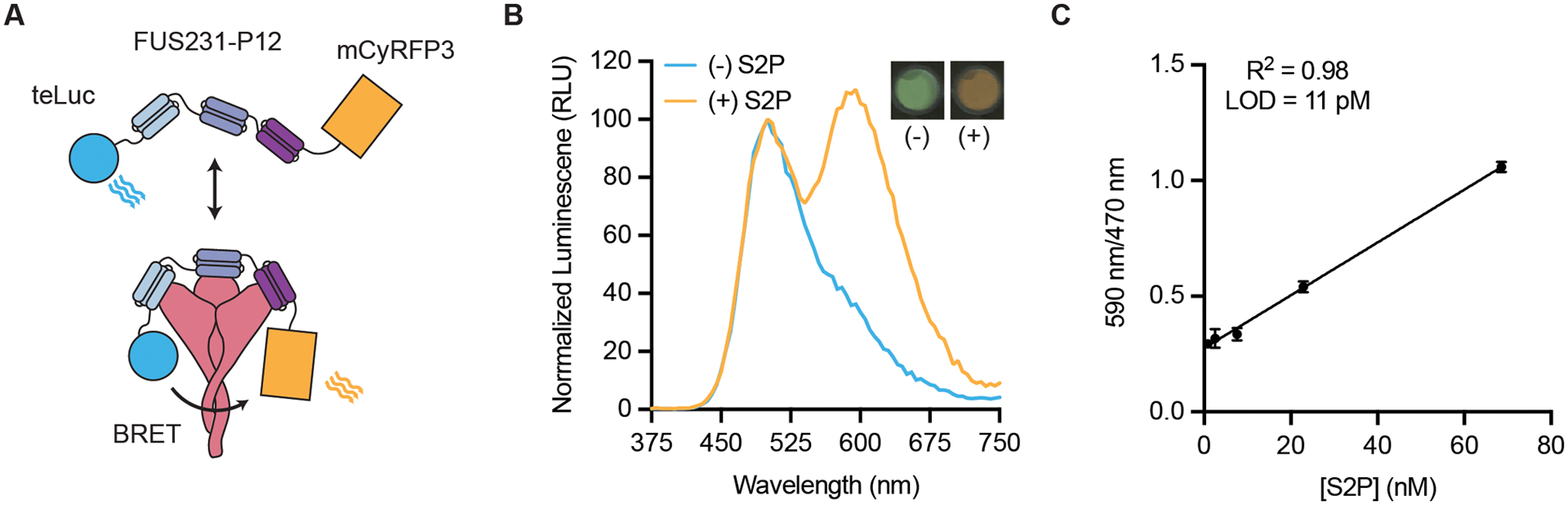
FUS231-P12 enables detection of SARS-CoV-2 S trimer through BRET. **(A)** A schematic representation of the BRET sensor, teluc-FUS231-P12-mCyRFP3, to detect S trimer is shown. **(B)** Luminescence emission spectra and image of the BRET sensor (100 pM) in the presence (orange trace, 100 pM) and absence (blue trace) of S2P are shown. Emission color change was observed using a mobile phone camera (inset top right). RLU, relative light units. **(C)** Titration of S2P with 100 pM sensor protein is shown. LOD indicates limit of detection. R^2^ value is shown on the graph. Data are presented as mean ± SEM with n = 3 technical replicates from a single experiment.

Having confirmed the binding mode of the FUS231 proteins by cryoEM, we designed an S trimer sensor, reasoning that the high affinity binding of the FUS231 proteins to the S trimer could make a useful diagnostic (50). We hypothesized that it would be possible to construct a bioluminescence resonance energy transfer (BRET) sensor for S trimer, where simultaneous engagement of all three minibinders in FUS231 with the S trimer would bring the N- and C-termini close enough together to enable efficient energy transfer. Towards this goal, we designed a BRET sensor based on FUS231-P12 with teLuc and mCyRFP3 fused to the N- and C-terminus of FUS231-P12 respectively (Fig. 3A) (51, 52). Upon binding of the sensor protein to a stabilized S protein with 2 proline mutations (S2P) (1, 50), we observed a 350% increase in the 590 nm:470 nm BRET ratio, which was not observed when bound to the RBD alone, and determined the limit of detection to be 11 pM S2P (Fig. 3B and C, and fig. S12). Furthermore, these results support the proposed multivalent binding mode for the FUS231 proteins.

### Multivalent minibinders bind tightly to SARS-CoV-2 variants.

**Fig 4.**
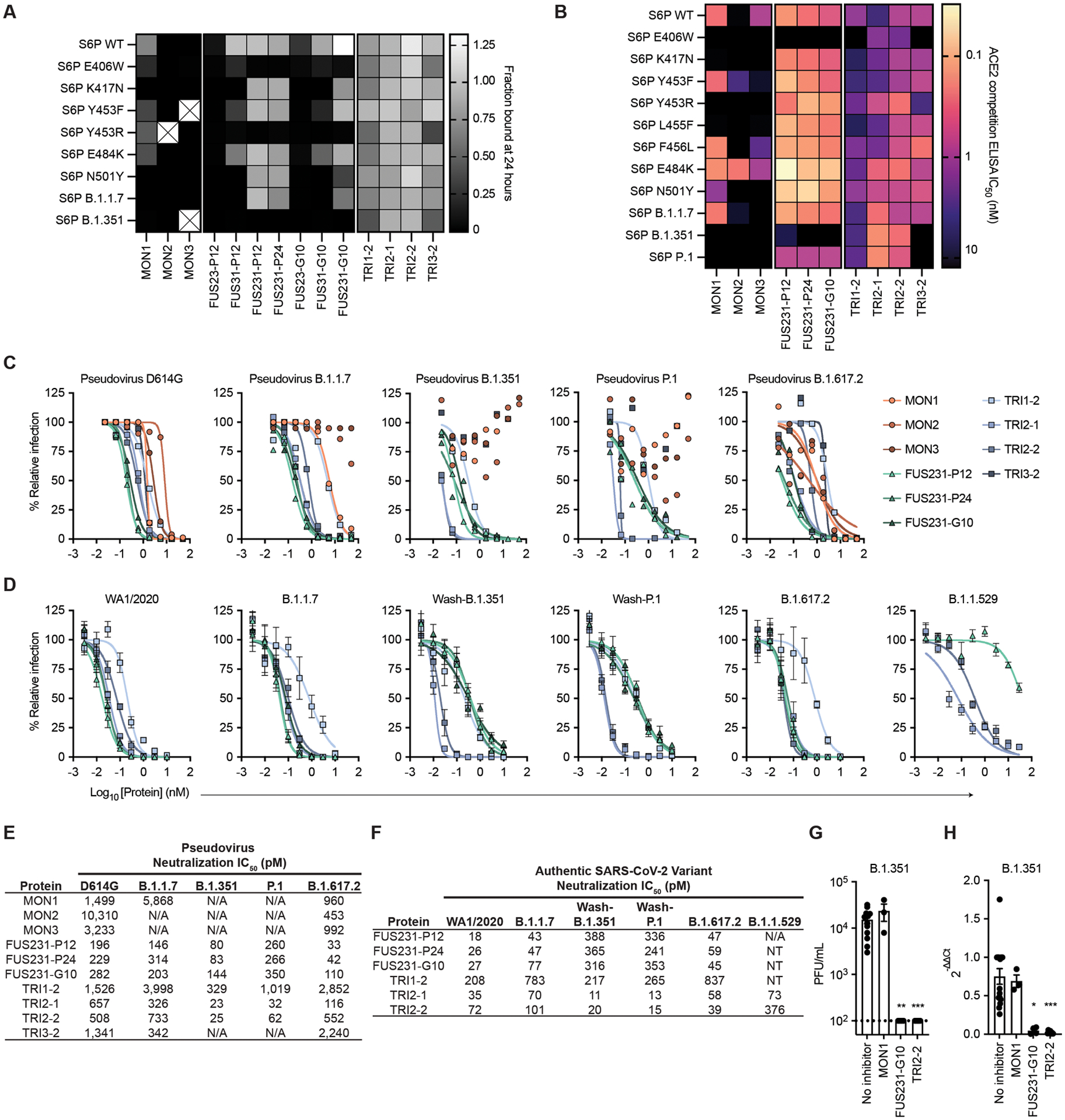
Multivalency enhances both the breadth and potency of neutralization against SARS-CoV-2 variants by minibinders. **(A)** Dissociation of minibinder constructs from S6P variants after 24 hours was measured by competition with untagged TRI2–1 using AlphaLISA. Means are shown with n = 3 technical replicates from a single experiment. Cells containing an X indicate insufficient signal in the absence of a competitor to quantify the fraction of protein bound. **(B)** Competition of minibinder constructs with ACE2 for binding S6P were measured by ELISA. Data are presented as mean values for n = 2 technical replicates representative of two independent experiments. **(C)** Neutralization of SARS-CoV-2 pseudovirus variants by minibinder constructs are shown. Data are presented as means of n = 2 technical replicates representative of two independent experiments. **(D)** Neutralization of authentic SARS-CoV-2 by minibinder constructs was measured. Data are presented as mean ± SEM with n = 4 technical replicates from two independent experiments for all but B.1.1.529, where n = 8 technical replicates from four independent experiments. **(E)** Summary of neutralization potencies of multivalent minibinder constructs against SARS-CoV-2 pseudovirus variants are shown. N/A indicates an IC_50_ value above the tested concentration range and an IC_50_ greater than 50,000 pM. **(F)** Summary of neutralization potencies of multivalent minibinder constructs against authentic SARS-CoV-2 variants are shown. N/A indicates an IC_50_ value above the tested concentration range and an IC_50_ greater than 30,000 pM. NT indicates not tested. **(G)** Replicating authentic B.1.351 virus in the presence of minibinder constructs (0.3 μM) was quantified in human kidney organoids. Data are presented as mean ± SEM, n = 4 biological replicates with 2 to 3 technical replicates per experiment. Data were compared to the no inhibitor control by a Kruskal-Wallis test with Dunn’s post-hoc analysis; ** P < 0.01, *** P < 0.001. Dashed line indicates lower limit of detection of plaque assay. **(H)** Relative gene expression of SARS-CoV-2 envelope protein (SARS-CoV2-E) was measured in kidney organoids post viral infection with and without multivalent minibinders (0.3 μM). Data are presented as mean ± SEM of n = 4 biological replicates with 2 to 3 technical replicates per experiment. Data were compared to the no inhibitor control by a Kruskal-Wallis test with Dunn’s post-hoc analysis; * P < 0.05, *** P < 0.001.

We next evaluated the resiliency of the binding of multivalent minibinders to the previously identified MON1 and MON2 escape mutants as well as mutations present in the B.1.1.7 (Alpha), B.1.351 (Beta), and P.1 (Gamma) SARS-CoV-2 VOCs. We first measured the off-rate of the best multivalent minibinders using competition AlphaLISA with TRI2–1 against a panel of mutant S glycoproteins (Fig. 4A). Multivalent minibinders were allowed to fully associate with mutant S trimers and subsequently were competed with 100-fold molar excess of untagged TRI2–1 to measure dissociation of the complex. The two-domain fusions (FUS23 and FUS31) did not show improved binding to the tested point mutants. The three-domain fusions (FUS231) retained binding to the tested mutants, indicating that they are more resistant to mutations than their monomeric counterparts, although E406W, Y453R, and the combination of K417N, E484K, and N501Y mutations (present in the B.1.351 S trimer) increased the dissociation rate more than 100-fold. Consistent with these results, we also observed increased dissociation rates for the FUS231 proteins against the B.1.351 and P.1 spikes by surface plasmon resonance (SPR) (fig. S13). The TRI1 and TRI3 homotrimers showed similar mutational tolerance in the competition experiment, with the same E406W, Y453R, and B.1.351 mutations causing increased dissociation rates. Strikingly, the TRI2 designs showed little dissociation after 24 hours against any of the tested S trimer mutants.

We subsequently screened the top multivalent minibinders for binding to mutant S trimers by an ACE2 competition enzyme-linked immunosorbent assay (ELISA), which correlates with neutralization potency (53). The minibinders were pre-incubated with the S6P variants before binding to immobilized ACE2 (Fig. 4B and fig. S14). In line with deep mutational scanning data, we observed impaired binding to the E406W, K417N, and Y453R mutants in addition to several other mutants. Two mutations, Y453F and E484K, improved MON2 binding, consistent with MON2 mimicry of the ACE2 interaction interface (38). Compared to the monovalent minibinders, we observed reduced effects of mutations in the competition IC_50_ values of the FUS231 and TRI2 minibinders and, to a lesser extent, of the TRI1 and TRI3 minibinders against the tested S6P variants, except for E406W (Fig. 4B and fig. S14D).

### Multivalent minibinders potently neutralize circulating SARS-CoV-2 variants.

To investigate the efficacy of the multivalent minibinders for preventing viral infection, we performed neutralization assays with the inhibitors using both pseudotyped lentiviruses and authentic SARS-CoV-2 variants (Fig. 4C to F, fig. S15). Against pseudoviruses displaying S proteins corresponding to the B.1.1.7, B.1.351, P.1, B.1.617.1, B.1.617.2 (Delta), and B.1.617.2.1 (Delta plus, AY.1) variants, all three monomer minibinders showed reduced neutralization capacity as compared to the Wuhan-Hu-1 D614G strain; in contrast, many of the multivalent minibinders were less affected in an ACE2 overexpressing cell line (Fig. 4C and E, and fig. S15A and C). The same proteins were also evaluated against pseudoviruses containing the E406W, L452R, and Y453F mutations, which again had little impact on neutralization for most multivalent minibinders tested (fig. S15A and C). This suggests that the increase in affinity from multivalency improved neutralization breadth. The top neutralizing minibinders from this screen were tested for neutralization of a panel of authentic SARS-CoV-2 viruses including a historical WA1/2020 strain, B.1.1.7, B.1.526 (Iota), B.1.1.529 (Omicron), B.1.617.1, B.1.617.2, and B.1.617.2.1 natural isolates, and chimeric WA1/2020 strains encoding spike genes corresponding to those of B.1.351 (Wash-B.1.351), and P.1 (Wash-P.1) variants. Again, the top candidates maintained pM-range IC_50_ values (Fig. 4D and F, and fig. S15B and D), except for the FUS231 proteins, which did not fully neutralize the B.1.1.529 variant in the tested concentration range. The TRI2 proteins maintained potent neutralization across all tested variants, notably including the B.1.1.7, Wash-B.1.351, Wash-P.1, B.1.617.2, and B.1.1.529 variants. Impressively, the TRI2 proteins potently neutralized the B.1.1.529 variant whereas many clinical mAbs for the treatment of COVID-19 do not (table S4) (15–17).

Although Vero-hACE2-TMPRSS2 (transmembrane serine protease 2) cells are useful for neutralization studies, they likely do not fully reflect the human cell infectivity. Recent findings highlight the relevance of using non-transformed human organoid models for SARS-CoV-2 research (54). SARS-CoV-2 can infect and replicate in human kidney organoids, specifically targeting kidney tubular epithelial cells expressing ACE2 receptors, responsible for viral entry (55, 56). Therefore, we generated kidney organoids from the H9 human embryonic stem cell line (57) (fig. S16) and evaluated the ability of the multivalent minibinders to prevent SARS-CoV-2 viral entry and replication. Replication of the B.1.351 variant was inhibited when the virus was pre-incubated with designed multivalent minibinders FUS231-G10 and TRI2–2, but not with MON1 (Fig. 4G). Quantitative reverse transcription PCR (RT-qPCR) analysis of viral RNA from the kidney organoids also showed reduced SARS-CoV-2 envelope protein (SARS-CoV2-E) gene expression in the presence of either FUS231-G10 or TRI2–2 (Fig. 4H). These data show that designed multivalent minibinders are potent neutralizers of the B.1.351 variant in a human organoid system.

### Multivalent minibinders resist viral escape.

**Fig 5.**
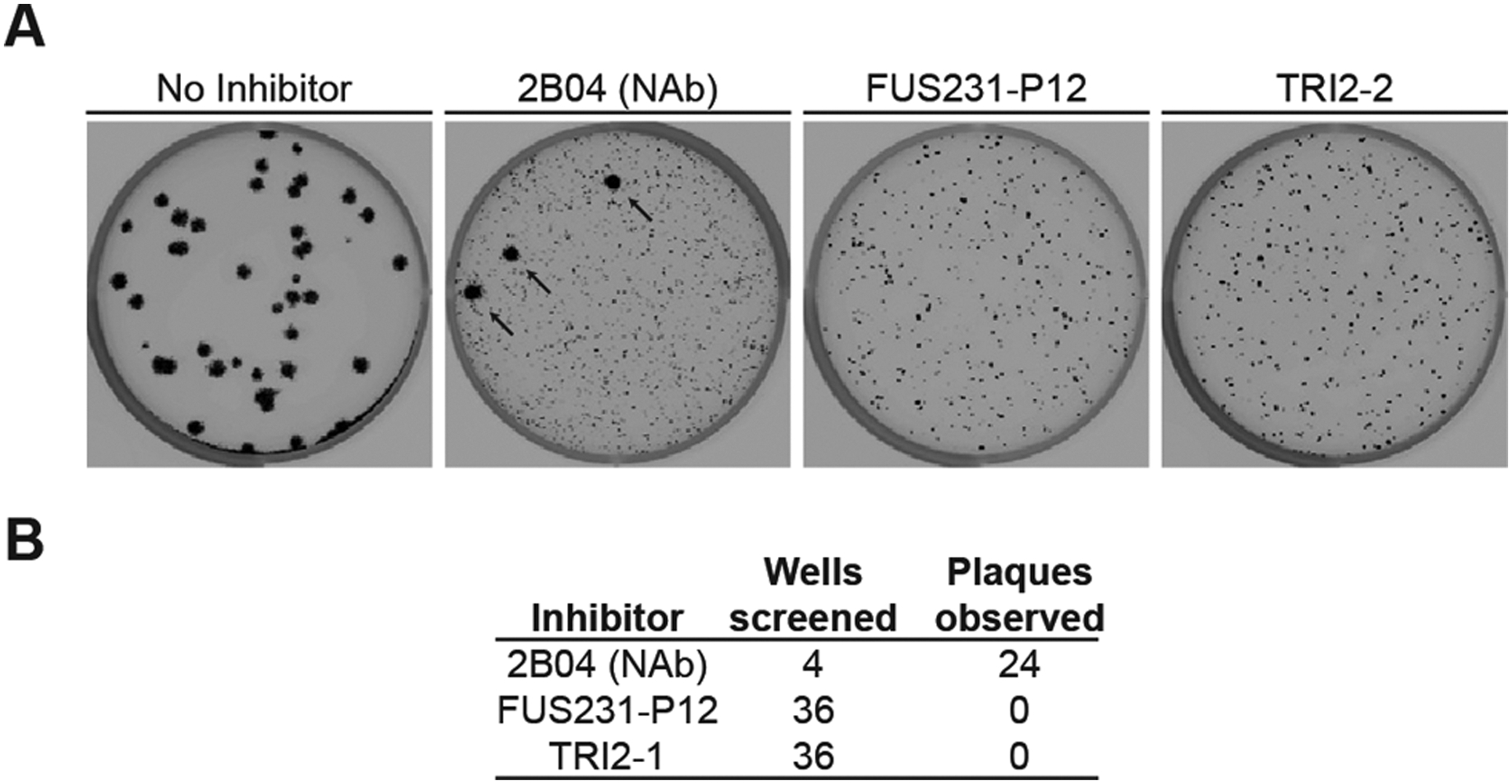
Top multivalent minibinder candidates are resistant to viral escape. **(A)** Plaque assays were performed to isolate VSV-SARS-CoV-2 S chimera virus escape mutants against a control neutralizing antibody (2B04) and the FUS231-P12 and TRI2–2 multivalent minibinders. For each inhibitor tested, Vero CCL-81 cells were incubated with VSV-SARS-CoV-2 S chimera virus for one hour, followed by addition of the inhibitor protein at a fully neutralizing concentration and further incubation to allow for replication and spread of resistant viruses. Thirty-six independent selections were carried out for each minibinder compound in a single experiment; representative examples are shown in the images. Small plaques are indicative of inhibited viral spreading and large plaques, highlighted by black arrows, are indicative of viral escape mutants spreading. **(B)** A summary of the results of the viral escape screen are shown. NAb, neutralizing antibody.

Given the promising data showing that multivalent minibinders can neutralize SARS-CoV-2 VOCs, we tested the multivalent minibinders for resistance against viral escape mutations in the S trimer (Fig. 5A and B) (6). Plaque assays were performed with a VSV-SARS-CoV-2 S chimera on Vero CCL-81 cells with minibinders included in the overlay to halt spread of non-resistant viruses. In positive control wells, inclusion in the overlay of 2B04, a potent neutralizing antibody targeting the RBD (6, 58–60), resulted in multiple escape mutants in each plate similar to previously reported escape mutants (Fig. 5A) (6). In contrast, for both FUS231-P12 and TRI2–2, escape mutants were not isolated in 36 replicate wells for each protein (fig. S17). These data indicate that both the FUS231-P12 and TRI2–2 proteins are more difficult to escape than 2B04. Given the known mutation rate of the VSV RNA polymerase L (61) and the number of viral particles screened, we estimated (table S5) that, for the multivalent minibinders, the screened pool of viral mutants contains a large fraction of the possible single amino acid substitutions (34% to 88%) and a small fraction of the possible double amino acid substitutions (0.4% to 9.6%) within the region of the RBD that contacts the minibinders. Taken together with the results of the single site saturation mutagenesis studies for the monovalent minibinders (fig. S2) these findings indicate that at least two or more mutations in the RBD are likely necessary to escape binding of the multivalent minibinders.

### Multivalent minibinder confers protection in human ACE2-expressing transgenic mice.

**Fig 6.**
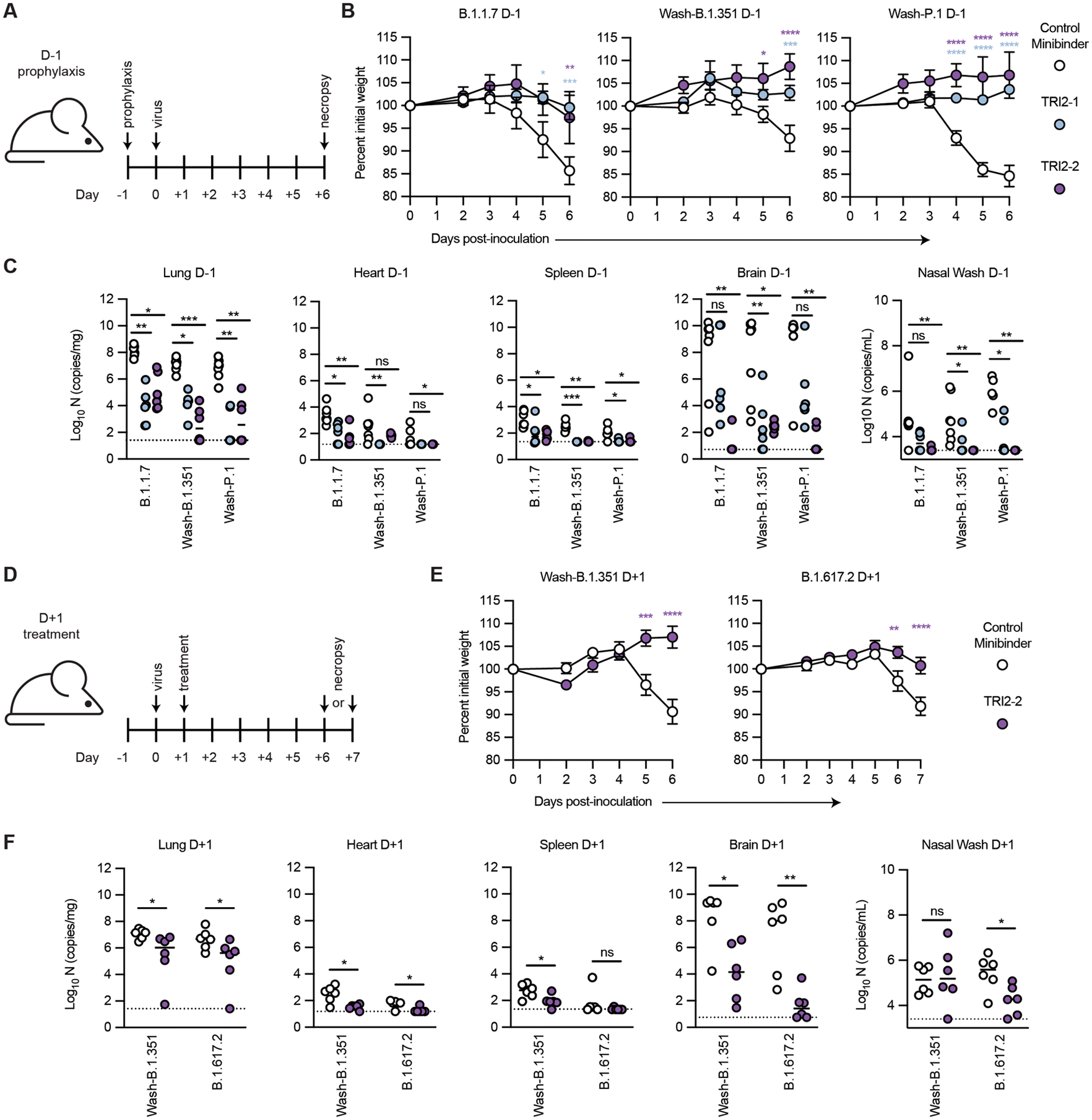
Top multivalent minibinder candidates protect mice from SARS-CoV-2 challenge. **(A)** K18-hACE2-transgenic mice (n = 6 from two independent experiments) were dosed with 50 μg of the indicated minibinder by intranasal (i.n.) administration (50 μl total) 24 hours prior (D-1) to infection with 10^3^ focus forming units (FFU) of SARS-CoV-2 variants B.1.1.7, Wash-B.1.351, or Wash-P.1 i.n. on Day 0. **(B)** Daily weight change following inoculation was measured. Data are presented as mean ± SEM. Data were analyzed by a two-way ANOVA with Sidak’s post-test; * P < 0.05, ** P < 0.01, *** P < 0.001, **** P < 0.0001 as compared to the control minibinder. **(C)** At 6 days post infection (dpi), animals (n = 6 from two independent experiments) were euthanized and analyzed for SARS-CoV-2 viral RNA by RT-qPCR in the lung, heart, spleen, brain, and nasal wash. Horizontal bars indicate median; dashed lines represent the limit of detection. Data were analyzed by a Kruskal-Wallis test with Dunn’s post-hoc analysis; ns, not significant, * P < 0.05, ** P < 0.01, *** P < 0.001. **(D)** K18-hACE2-transgenic mice (n = 6 from two independent experiments) were dosed with 50 μg of the indicated minibinder by i.n. administration (50 μl total) 24 hours after (D+1) infection with 10^3^ FFU of the SARS-CoV-2 Wash-B.1.351 or B.1.617.2 variant on Day 0. **(E)** Daily weight change following inoculation was measured. Data are presented as mean ± SEM. Data were analyzed by two-way ANOVA with Sidak’s post-test; * P < 0.05, ** P < 0.01, *** P < 0.001, **** P < 0.0001). **(F)** At 6 dpi (B.1.351) or 7 dpi (B.1.617.2), animals (n = 6 from two independent experiments) were euthanized and analyzed for SARS-CoV-2 viral RNA by RT-qPCR in the lung, heart, spleen, brain, and nasal wash. Horizontal bars indicate median; dashed lines represent the limit of detection. Data were analyzed by a two-tailed Mann-Whitney test; ns, not significant, * P < 0.05, ** P < 0.01.

To determine whether the multivalent minibinders can prevent or treat SARS-CoV-2 infection in vivo, we performed pre-exposure prophylaxis or post-exposure therapy studies in highly susceptible K18-hACE2 transgenic mice (62) with TRI2 multivalent minibinders, which retained the most consistent binding to all S trimer variants tested. For prophylaxis, a single 50 μg dose (about 2.5 mg/kg) of TRI2–1 or TRI2–2 was administered directly to the nasal cavity (intranasal administration) one day prior to inoculation with 10^3^ focus forming units (FFU) of the indicated SARS-CoV-2 VOCs (Fig. 6A). In all cases, intranasal administration of TRI2–1 or TRI2–2 protected mice against SARS-CoV-2-induced weight loss (Fig. 6B). At 6 days post infection, viral burden in tissues was reduced in almost all primary (lung and nasal wash) and secondary sites (heart, spleen, brain) of viral replication in TRI2–1 and TRI2–2 treated animals (Fig. 6C). To determine the therapeutic potential of TRI2–2, we inoculated K18-hACE2 mice with 10^3^ FFU of Wash-B.1.351 or B.1.617.2 and one day later, administered a single 50 μg dose of minibinder intranasally (Fig. 6D). Treatment with TRI2–2 protected against weight loss and reduced viral burden in all tissues except nasal washes (Wash-B.1.351) or the spleen (B.1.617.2) (Fig. 6E and F). TRI2–2 therapy at D+1 reduced infectious virus titers in the lungs of Wash-B.1.351- and B.1.617.2-infected mice (fig. S18). We determined the pharmacokinetics of TRI2–2 after intranasal administration by quantitative competition ELISA. Substantial concentrations of TRI2–2 were detected in the lung lysate and serum 48 hours after administration (fig. S19) but was too low for confident quantification in nasal turbinates after the first time point and for confident quantification in nasal washes at all time points. These results indicate that intranasal administration of TRI2–1 or TRI2–2 confer protection against SARS-CoV-2 infection as both pre-exposure prophylaxis and post-exposure therapy in a stringent model of disease.

## DISCUSSION

Both strategies for generating multivalent S protein binders from miniproteins, self-assembling homotrimers (TRI) and multi-domain fusions (FUS), yielded designs with improved affinity, neutralization of current and historical VOCs, and resistance to escape mutants over their monovalent counterparts (25, 26). The TRI2 proteins maintained the strongest binding across all S trimer variants tested, likely because MON2 is an ACE2 mimic, similar to the recently reported S2K146 mAb (15, 24). This combination of trivalency and receptor mimicry could be a useful general approach for combating viral escape and antigenic drift (15, 24, 36, 53, 63, 64).

The designs also have potential advantages as therapeutics over ACE2 receptor traps and mAbs. When compared to receptor traps (55, 65–67), TRI2–2 has a low risk of eliciting host-directed anti ACE2 responses due to low sequence similarity between MON2 and ACE2 (fig. S1). On a per mass basis, the TRI2 proteins are more potent neutralizers than all currently authorized mAbs for the treatment of COVID-19 (15, 16), and, unlike most clinical mAbs, they maintain activity against Omicron (table S4). The multivalent minibinders are amenable to large-scale production in microorganisms like *E. coli*, making them more cost-effective to manufacture than mAbs (8). Furthermore, their small size and stability may enable direct nebulization into the human upper respiratory tract (3, 68–70), a strategy that could increase accessibility for patients over the typical intravenous or subcutaneous routes used for administering neutralizing mAbs.

The high potency of the multivalent constructs, in particular TRI2–2 against Omicron, Delta, and the other tested VOCs, makes them promising candidate SARS-CoV-2 therapeutics, and they are currently undergoing further preclinical development and investigational new drug (IND) enabling studies. These efforts will address limitations in our current study. First, anti-drug antibodies are a concern with non-human proteins and, although MON1 and other minibinders (26, 71) elicit little or no immune response, additional studies are required to determine the immunogenicity of the multivalent constructs. Second, it will be important to assess the pharmacokinetics following different modes of administration; in humans, it may be necessary to distribute the compound deeper into the respiratory system for post infection efficacy. Third, as with any new drug candidate going through the drug development pipeline, it will be necessary to assess its stability as well as its potency and toxicity after prolonged administration.

In summary, our integration of structure-guided computational protein design, cell-free DNA assembly, cell-free expression, and a competition-based off-rate screen enabled the rapid design and optimization of S trimer-engaging multivalent minibinders. Scaling cell-free expression to manufacture mg quantities of endotoxin-free protein for cell-based neutralization assays further reduced the time required to evaluate lead molecules. The developed pipeline has direct relevance to diagnostics as well; the FUS231-based BRET sensor is easy to use, fast, and has the potential to be less expensive than state-of-the-art lateral flow assay-based antigen tests (72, 73). Our integrated computational and experimental pipeline should enable the rapid generation of potent protein-based medical countermeasures and diagnostic reagents against newly emerging pathogens.

## MATERIALS AND METHODS

### Study design

The objective of this study was to design and evaluate multivalent minibinders that neutralize SARS-CoV-2 variants containing mutations within the RBD. At the outset, we hypothesized that multivalency would overcome mutations that reduce binding for individual monomeric minibinders. Designed proteins were evaluated in controlled laboratory experiments, first using biophysical methods with purified proteins (AlphaLISA and ELISA competition assays) followed by in vitro methods requiring cell culture (pseudovirus and authentic virus neutralization assays). The top candidates from neutralization assays were screened by electron microscopy for cross-linking multiple S trimers and the candidates that were found to minimally cross-link S trimers were subjected to structural analysis by cryoEM. The most promising proteins were evaluated in vivo in mice. In all studies where cell lines were used, the cell line is noted in the corresponding materials and methods section. The total number and type of experimental replicates is noted in each figure legend. Details on the in vivo mouse study compliance with best practices can be found in the corresponding materials and methods section. No sample-size calculations were performed to power each in vivo study. Instead, sample sizes and study endpoints were determined based on previous in vivo virus challenge experiments. For all other experiments, sample size was selected based on previous literature and previous experience. In the animal studies, mice were randomly assigned to the control and treatment groups. Animal caretakers and researchers were not blinded to the study groups or during the assessment of the outcomes. Data that underlie the results reported in this article can be found in data file S2, data file S3, and in the deposited data listed in the data and materials availability statement.

### Statistical analysis

Statistical significance was determined by a P value < 0.05 using GraphPad Prism 9 software. Only non-parametric tests were used throughout this article. Analysis of mouse weight changes was performed using a two-way analysis of variance (ANOVA) with Sidak’s post-test for multiple comparisons. Statistical analysis of viral load between two groups was performed using either a Kruskal-Wallis test with Dunn’s post-hoc analysis for multiple comparisons or a two-tailed Mann-Whitney test as noted in the corresponding figure captions.

## Supplementary Material

DataFileS1

Supp

DataFileS2

DataFileS3

## Data Availability

All data associated with this study are in the paper or supplementary materials. Structural models and density maps have been deposited in the Protein Data Bank (PDB) (SARS-CoV-2/TRI2–2: 7UHC and SARS-CoV-2/TRI2–2 (local refinement): 7UHB) and Electron Microscopy Data Bank (EMDB) (SARS-CoV-2/TRI2–2: EMD-26512, SARS-CoV-2/TRI2–2 (local refinement): EMD-26511, SARS-CoV-2/FUS31-G10 (2RBD-open): EMD-26509, SARS-CoV-2/FUS31-G10 (3RBD-open): EMD-26510, SARS-CoV-2/FUS231-P24 (2RBD-open): EMD-26507, and SARS-CoV-2/FUS231-P24 (3RBD-open): EMD-26508). Illumina sequencing data for the deep mutational scanning experiments are available on NCBI SRA, BioSample SAMN19925005. Code for the analysis of the deep mutational scanning experiments are available on Zenodo https://doi.org/10.5281/zenodo.6377268. Requests for reagents (antibodies, viruses, and other proteins) should be directed to the corresponding authors and will be made available after completion of a Materials Transfer Agreement with the University of Washington. This work is licensed under a Creative Commons Attribution 4.0 International (CC BY 4.0) license, which permits unrestricted use, distribution, and reproduction in any medium, provided the original work is properly cited. To view a copy of this license, visit http://creativecommons.org/licenses/by/4.0/. This license does not apply to figures/photos/artwork or other content included in the article that is credited to a third party; obtain authorization from the rights holder before using this material.
