# Supplementary material for: Multivalent designed proteins neutralize SARS-CoV-2 variants of concern and confer protection against infection in mice
Source: Sci Transl Med. Author manuscript; Available in PMC 2022 Jul 6. (PMC9258422; doi:10.1126/scitranslmed.abn1252)
Supplement: Supp [file NIHMS1816067-supplement-Supp.pdf]

Supplementary Materials for

Title: Multivalent designed proteins neutralize SARS-CoV-2 variants of concern and confer protection against infection in mice

Authors: Andrew C. Hunt[1,2†], James Brett Case[3†], Young-Jun Park[4†], Longxing Cao[4,5†], Kejia Wu[4,5†], Alexandra C. Walls[4,6†], Zhuoming Liu[7], John E. Bowen[4], Hsien-Wei Yeh[4,5], Shally Saini[4,8], Louisa Helms[8,9,10,24], Yan Ting Zhao[4,8,11], Tien-Ying Hsiang[12], Tyler N. Starr[13], Inna Goreshnik[4,5], Lisa Kozodoy[4,5], Lauren Carter[4,5], Rashmi Ravichandran[4,5], Lydia B. Green[14], Wadim L. Matochko[14], Christy A. Thomson[14], Bastian Vögeli[1,2,15], Antje Krüger[1,2], Laura A. VanBlargan[3], Rita E. Chen[3,16], Baoling Ying[3], Adam L. Bailey[16,17], Natasha M. Kafai[3,16], Scott E. Boyken[4,5], Ajasja Ljubetič[4,5,18], Natasha Edman[4,5,19,20], George Ueda[4,5], Cameron M. Chow[4,5,21], Max Johnson[4,5], Amin Addetia[4,22], Mary Jane Navarro[4], Nuttada Panpradist[23], Michael Gale Jr.[12], Benjamin S. Freedman[8,9,10,23,24], Jesse D. Bloom[13,6,25], Hannele Ruohola-Baker[4,8,11,23], Sean P. J. Whelan[7], Lance Stewart[4,5], Michael S. Diamond[3,7,16,26*], David Veesler[4,6*], Michael C. Jewett[1,2,27,28*], David Baker[4,5,6*]

Affiliations:

[1]Department of Chemical and Biological Engineering, Northwestern University, Evanston, IL, 60208, USA

[2]Center for Synthetic Biology, Northwestern University, Evanston, IL, 60208, USA,

[3]Department of Medicine, Washington University School of Medicine, St. Louis, MO, 63110, USA

[4]Department of Biochemistry, University of Washington, Seattle, WA, 98195, USA

[5]Institute for Protein Design, University of Washington, Seattle, WA, 98195, USA

[6]Howard Hughes Medical Institute, University of Washington, Seattle, WA, 98195, USA

[7]Department of Molecular Microbiology, Washington University School of Medicine, St. Louis, MO, 63110, USA

[8]Institute for Stem Cell and Regenerative Medicine, University of Washington School of Medicine, Seattle, WA, 98109, USA

[9]Division of Nephrology, Department of Medicine, University of Washington School of Medicine, Seattle, WA, 98109, USA

[10]Kidney Research Institute, University of Washington School of Medicine, Seattle, WA, 98109, USA

[11]Oral Health Sciences, School of Dentistry, University of Washington, Seattle, WA, 98195, USA

[12]Department of Immunology, Center for Innate Immunity and Immune Disease, University of Washington, Seattle, WA, 98195, USA

[13]Basic Sciences Division, Fred Hutchinson Cancer Research Center, Seattle, WA, 98109, USA
[14]Amgen Research, Biologic Discovery, Burnaby, V5A 1V7, BC, Canada
[15]Invizyne Technologies Inc., Monrovia, CA, 91016, USA
[16]Department of Pathology & Immunology, Washington University School of Medicine, St. Louis, MO, 63110, USA
[17]Department of Pathology & Laboratory Medicine, University of Wisconsin – Madison, Madison, WI, 53705, USA
[18]Department for Synthetic Biology and Immunology, National Institute of Chemistry, Ljubljana, SI-1000, Slovenia
[19]Molecular and Cellular Biology Graduate Program, University of Washington, Seattle, WA, 98195, USA
[20]USA Medical Scientist Training Program, University of Washington, Seattle, WA, 98195, USA
[21]Neolukin Therapeutics Inc., Seattle, WA, 98102, USA
[22]The Molecular and Cellular Biology Program, University of Washington, Seattle, WA, 98195, USA
[23]Department of Bioengineering, University of Washington, Seattle, WA, 98195, USA
[24]Department of Laboratory Medicine and Pathology, University of Washington School of Medicine, Seattle, WA, 98109, USA
[25]Department of Genome Sciences, University of Washington, Seattle, WA, 98195, USA
[26]Andrew M. and Jane M. Bursky Center for Human Immunology and Immunotherapy Programs, Washington University School of Medicine, St. Louis, MO, 63110, USA
[27]Chemistry of Life Processes Institute, Northwestern University, Evanston, IL, 60208, USA
[28]Robert H. Lurie Comprehensive Cancer Center, Northwestern University, Chicago, IL, 60611, USA
[†]These authors contributed equally

***Corresponding authors:** Michael S. Diamond (mdiamond@wustl.edu), David Veesler (dveesler@uw.edu), Michael C. Jewett (m-jewett@northwestern.edu), David Baker (dabaker@uw.edu)

This PDF file includes:

Materials and Methods
Fig. S1. Sequence alignment of AHB2 and ACE2.
Fig. S2. Site saturation mutagenesis of RBD to predict MON1 (LCB1v2.2) and MON2 (AHB2) escape mutants.

Fig. S3. Cell-free DNA assembly and protein synthesis of multivalent minibinders.
Fig. S4. Off-rate screen of multivalent minibinders.
Fig. S5. Kinetics of MON1, MON2, and MON3 binding to the SARS-CoV-2 RBD.
Fig. S6. Heat-based purification of mono- and multivalent minibinders.
Fig. S7. Negative stain EM analysis of minibinder-mediated crosslinking and aggregation of S trimers.
Fig. S8. Cryo-EM data collection and processing of the S6P/TRI2-2, S6P/FUS31-G10, and S6P/FUS231-P24 datasets.
Fig. S9. Cryo-EM processing scheme of SARS-CoV-2 S/TRI2-2 complex.
Fig. S10. Cryo-EM processing scheme of SARS-CoV-2 S/FUS31-G10 complex.
Fig. S11. Cryo-EM processing scheme of SARS-CoV-2 S/FUS231-P24 complex.
Fig. S12. FUS231-P12 BRET sensor does not detect monomeric RBD.
Fig. S13. Kinetic analysis of interactions between multivalent minibinders and SARS-CoV-2 S protein variants using surface plasmon resonance.
Fig. S14. Competition of ACE2 and mini binder constructs for S6P.
Fig. S15. Neutralization of additional SARS-CoV-2 variants.
Fig. S16. Representative confocal images of human kidney organoids.
Fig. S17. Replicate plates for VSV-SARS-CoV-2 escape studies.
Fig. S18. Measurement of SARS-CoV-2 viral titers in the lung of K18-hACE2-transgenic mice post intranasal delivery of TRI2-2.
Fig. S19. Pharmacokinetics of TRI2-2 delivered intranasally in C57BL/6J mice.
Table S1. List of abbreviations used to describe multivalent minibinders in this article.
Table S2. Oligomerization domains tested in this work.
Table S3. CryoEM data collection and refinement statistics.
Table S4. Comparison of multivalent minibinder and FDA authorized neutralizing antibody potencies.
Table S5. Estimates of the diversity of mutants in the VSV-SARS-CoV-2 chimera virus pool used in the multivalent minibinder escape selections.

Other Supplementary Material for this article includes the following:

Data File S1 (.xlsx). List of DNA and protein sequences for multivalent minibinders used in this article.
Data File S2 (.xlsx). Individual data for all main text figures.
Data File S3 (.xlsx). Individual data for all supplementary figures.

Materials and Methods

Cell lines and cell culture

For production of S protein variants, Expi293F cells (Thermo Fisher Scientific, A14527) were grown in Expi293 Expression Medium (Gibco), cultured at 37°C with 8% CO_2 and shaking at 130 rpm. For pseudovirus neutralization studies, the HEK293T/17 female human embryonic kidney cell line was obtained from the American Type Culture Collection (ATCC, CRL-11268). The HEK-ACE2 adherent cell line was obtained through Biodefense and Emerging Infections Research Resources Repository (BEI Resources), NIAID, NIH: NR-52511 *(74)*. All adherent cells were cultured at 37°C with 8% CO_2 in flasks with Dulbecco's Modified Eagle medium (DMEM) + 10% fetal bovine serum (FBS, HyClone) + 1% penicillin-streptomycin. Cell lines were not tested for mycoplasma contamination nor authenticated. For authentic SARS-CoV-2 studies, Vero CCL-81 (ATCC, CCL-81; RRID:CVCL_0059), Vero-TMPRSS2 (trans membrane serine protease 2), and Vero-hACE2-TMPRSS2 (human angiotensin converting enzyme 2) (a gift of A. Creanga and B. Graham, NIH) were cultured at 37°C in DMEM supplemented with 10% FBS, 10 mM HEPES pH 7.3, 1 mM sodium pyruvate, 1× non-essential amino acids, and 100 U/ml of penicillin–streptomycin. Additionally, Vero-TMPRSS2 and Vero-hACE2-TMPRSS2 cells were cultured in the presence of 5 μg/mL of blasticidin or puromycin, respectively. The WA1/2020 (2019n-CoV/USA_WA1/2020) isolate of SARS-CoV-2 was obtained from the US Centers for Disease Control (CDC). WA1/2020 stocks were propagated on Vero CCL-81 cells and used at passage 6. The B.1.1.7, Wash-B.1.351, and Wash-P.1 (previously described as Wash-B.1.1.28 and Wash BR-B.1.1.248) viruses

have been described previously *(12, 75)*. B.1.526 (S477N), B.1.617.1, B.1.617.2, B.1.617.2.1 (AY.1), B.1.1.529 viruses were isolated from infected individuals. For all strains, infectious stocks were propagated by inoculating Vero CCL-81 or Vero-TMPRSS2 cells. Supernatant was collected, aliquoted, and stored at -80°C. All work with infectious SARS-CoV-2 was performed in Institutional Biosafety Committee-approved BSL3 and A-BSL3 facilities at Washington University School of Medicine using positive pressure air respirators and protective equipment. All virus stocks were deep sequenced after RNA extraction to confirm the presence of the anticipated substitutions. For VSV SARS-CoV-2 chimera escape selections, Vero CCL-81 (ATCC, CCL-81; RRID: CVCL_0059) and MA104 (a gift from H. B. Greenberg, Stanford School of Medicine) were used as described previously *(76)*.

LCB1 (MON1) and LCB3 (MON3) optimization

Site saturation mutagenesis data were collected for LCB1 and LCB3 using the method described previously *(25)*. Beneficial mutations that showed increased binding with RBD were selected and used to construct a Resfile *(77)*. 10,000 sequence design trajectories were performed using the Rosetta FastDesign protocol, constrained by the Resfile. Eight sequences were selected based on rosetta score and binding ddG for LCB1 and LCB3 respectively, while keeping the sequence diversity. Genes encoding the selected sequences were cloned into modified pET-29b(+) *E. coli* plasmid expression vectors (GenScript, N-terminal 8 His-tag followed by a tobacco etch virus (TEV) cleavage site) for checking the expression yield (the expression yield is often correlated with protein solubility and stability) and the binding with RBD was characterized with the AlphaLISA

assay (see below). The sequences with high expression yield, as well as tight binding affinity with RBD were selected for downstream multivalent constructs design, with the names as LCB1_v2.2 and LCB3_v2.2.

Multivalent fusion constructs design

The CryoEM structures of LCB1 and LCB3 in complex with the spike protein, and the design model of AHB2, were used to determine the order of the monomers in the multivalent fusion constructs. The structures/models were firstly aligned using the spike protein as the reference and the order was generally determined by the shortest distance between the n- to c-terminus of the two minibinders (e.g., the AHB2 c-terminus is closer to the LCB1 n-terminus than vice versa). The combinations: AHB2-LCB1, LCB3-LCB1, AHB2-LCB3, LCB3-AHB2, LCB3-AHB2-LCB1, and AHB2-LCB2-LCB1 were tested with either a flexible glycine-serine (G) linker or a proline-alanine-serine (P) linker *(46)*. The linker length was optimized using the AlphaLISA assay and screening by negative stain EM (see below).

Scaffold selection and backbone generation for self-assembling homotrimer design

De novo designed C3 symmetric protein scaffolds and de novo designed helical repeat proteins (DHRs) were the basis for the generation of trimeric mini-binders. Thirty-four designed C3 scaffolds and 62 DHRs were selected after preliminary screening based on the geometry matching with the trimeric SARS-CoV-2 S protein and scaffold quality. Fusing the three components (C3 scaffolds, DHRs and designed mini-binders) together

through a modified version of the WORMS *(78)* software, 1096 trimeric binder backbones were generated. This was followed by steric clashing filtering (Rosetta centroid energy < 10), visual inspection in a molecular graphics viewer (Pymol), and sequence design for residues within 9 Å from the fused junction. Fifty-six designs were selected for further validation. To ensure the matching geometry between the fused constructs and the SARS-CoV-2 S trimer with three RBDs in the open state (PDB: 7CAK), the WORMS package was customized with the "stack" orientation implemented, where the two cyclic symmetrical axes from both C3 scaffolds and S trimer are aligned along Z axis.

Junction modification and flexibility insertion for self-assembling homotrimer design

Considering the strict geometric requirement, the dynamic nature of the S trimer, and the low expression successful rate, two new approaches have been pursued to simplify the constructs and enhance the flexibility. The first approach was creating semi-flexible binders. Starting from the high-quality rigid fusion models with a relatively small size (less than 250 amino acids in total), DHR junctions were omitted. Instead, the last one or two helices from the C3 scaffolds were modified, truncated, or extended with one or two helices using blueprint-based backbone generation *(79, 80)*. The newly generated last helix was required to be within 12 Å to the first helix from minibinders in models. (Glycine-Serine)x1 to (Glycine-Serine)x6 were modeled in Pymol as the flexible linker between the modified C3 scaffolds and mini-binders. Based on the success of this approach, the second was introduced to simplify the constructs further with short flexible linkers only. Four de novo C3 scaffolds and six native C3 scaffolds were selected as final candidates

with the right geometry, some of which confirmed by the solved cryo-electron microscopy structures. The modification on the last helix was allowed within two to ten residues to be as geometrically compatible as previously. The sequence was optimized based on Rosetta combinatorial sequence optimization packages *(81–83)* and homology models.

Deep mutational scanning profiles of minibinder escape

Minibinder escape mapping experiments were performed in biological duplicate using a deep mutational scanning approach as previously described *(7, 84)*. Briefly, yeast-surface display libraries expressing 3,804 of the 3,819 possible amino acid mutations in the SARS-CoV-2 receptor binding domain (RBD) (Wuhan-Hu-1 sequence, Genbank MN908947, residues N331-T531) were constructed in *(38)*, and sorted to purge mutations that abolish RBD folding or ACE2 binding. Libraries were induced for RBD surface expression and labeled with minibinder at the concentration of the 90% maximal effective concentration (EC_{90}) for binding to yeast-displayed RBD ("sensitive" selection) or 400 ng/mL ("stringent" selection), the concentration used for prior selections of clinical antibodies *(7, 85)*, followed by secondary labeling with 1:100 fluorescein isothiocyanate (FITC)-conjugated anti-Myc (Immunology Consultants Lab, CMYC-45F), and 1:100 iFluor-647-conjugated mouse anti-His tag antibody (Genscript A01802). Library variants that escape miniprotein binding were sorted on a BD FACSAria II based on gates drawn from control populations labeled at 0.1x ("sensitive" selection) or 0.01x ("stringent" selection) the selection concentration, as shown in fig. S2A to C. For each sample, at least 10 million RBD^+ cells were processed, and minibinder-escape cells were sorted and grown overnight. The plasmid was purified and sequenced on a HiSeq 2500. Escape

fractions were computed from sequencing counts exactly as described in Starr *et al*. *(7)*. Illumina sequencing counts are available from the NCBI SRA (BioProject SAMN19925005). All code and analysis steps are described on GitHub: https://github.com/jbloomlab/SARS-CoV-2-RBD_MAP_minibinders and Zenodo: https://doi.org/10.5281/zenodo.6377268. A table reporting all mutation escape fractions is available: https://github.com/jbloomlab/SARS-CoV-2-RBD_MAP_minibinders/blob/main/results/supp_data/IPD_ligands_raw_data.csv.

Cell-free DNA assembly and CFPS template preparation

Proteins to be manufactured using CFPS were codon optimized using the Integrated DNA Technologies (IDT) codon optimization tool and ordered as gblocks containing the pJL1 5' (tttgtttaactttaagaaggagatatacat) and 3' (gtcgaccggctgctaacaaagcccgaaagg) Gibson assembly overhangs. DNA was resuspended at a concentration of 50 ng/µL. Linearized pJL1 plasmid backbone (Addgene plasmid # 69496) was ordered as a gblock from IDT (see below) and amplified using the pJL1_F (gtcgaccggctgcta) and pJL1_R (atgtatatctccttcttaaagttaaacaaaattatttcta) primers by PCR using Q5 Hot Start DNA polymerase (New England Biolabs (NEB), M0493L) following manufacturer instructions. Amplified pJL1 backbone was purified using the DNA Clean and Concentrate Kit (Zymo Research, D4006) and diluted to a concentration of 50 ng/µL.

Linearized pJL1 plasmid backbone:

gtcgaccggctgctaacaaagcccgaaaggaagctgagttggctgctgccaccgctgagcaataactagcataacccct
tggggcctctaaacgggtcttgaggggttttttgctgaaagccaattctgattagaaaaactcatcgagcatcaaatgaaact

gcaatttattcatatcaggattatcaataccatatttttgaaaaagccgtttctgtaatgaaggagaaaactcaccgaggcagt
tccataggatggcaagatcctggtatcggtctgcgattccgactcgtccaacatcaatacaacctattaatttcccctcgtcaa
aaataaggttatcaagtgagaaatcaccatgagtgacgactgaatccggtgagaatggcaaaagcttatgcatttctttcca
gacttgttcaacaggccagccattacgctcgtcatcaaaatcactcgcatcaaccaaaccgttattcattcgtgattgcgcct
gagcgagacgaaatacgcgatcgctgttaaaaggacaattacaaacaggaatcgaatgcaaccggcgcaggaacac
tgccagcgcatcaacaatattttcacctgaatcaggatattcttctaatacctggaatgctgttttcccggggatcgcagtggtg
agtaaccatgcatcatcaggagtacggataaaatgcttgatggtcggaagaggcataaattccgtcagccagtttagtctg
accatctcatctgtaacatcattggcaacgctacctttgccatgtttcagaaacaactctggcgcatcgggcttcccatacaat
cgatagattgtcgcacctgattgcccgacattatcgcgagcccatttatacccatataaatcagcatccatgttggaatttaat
cgcggcttcgagcaagacgtttcccgttgaatatggctcataacaccccttgtattactgtttatgtaagcagacagttttattgtt
catgatgatatatttttatcttgtgcaatgtaacatcagagattttgagacacaacgtgagatcaaaggatcttcttgagatcctt
tttttctgcgcgtaatctgctgcttgcaaacaaaaaaaccaccgctaccagcggtggtttgtttgccggatcaagagctacca
actctttttccgaaggtaactggcttcagcagagcgcagataccaaatactgttcttctagtgtagccgtagttaggccacca
cttcaagaactctgtagcaccgcctacatacctcgctctgctaatcctgttaccagtggctgctgccagtggcgataagtcgt
gtcttaccgggttggactcaagacgatagttaccggataaggcgcagcggtcgggctgaacggggggttcgtgcacaca
gcccagcttggagcgaacgacctacaccgaactgagatacctacagcgtgagctatgagaaagcgccacgcttcccga
agggagaaaggcggacaggtatccggtaagcggcagggtcggaacaggagagcgcacgagggagcttccagggg
gaaacgcctggtatctttatagtcctgtcgggtttcgccacctctgacttgagcgtcgatttttgtgatgctcgtcaggggggcg
gagcctatggaaaaacgccagcaacgcgatcccgcgaaattaatacgactcactatagggagaccacaacggtttccc
tctagaaataattttgtttaactttaagaaggagatatacat

Gibson assembly was used to assemble protein open reading frame DNA with the pJL1 backbone following the published protocol with the addition 3.125 µg/mL of ET SSB (NEB,

product no. M2401S) *(86, 87)*. Twenty ng of purified, linearized pJL1 backbone and 20 ng of the protein open reading frame insert were combined in 2 µL Gibson assembly reactions and incubated at 50°C for 30 minutes. Similar to other published methods *(40, 88)*, the unpurified assembly reactions were diluted in 40 µL of nuclease free water (Thermo Fisher Scientific, AM9937) and 1 µL of the diluted reaction were used as the template for a PCR to generate linear expression templates (LETs) for CFPS. LETs were amplified by PCR using the pJL1_LET_F (ctgagatacctacagcgtgagc) and pJL1_LET_R (cgtcactcatggtgatttctcacttg) primers and the Q5 Hot Start DNA polymerase (NEB, M0493L) following manufacturer instructions.

CFPS cell extract preparation, reactions, and purification

Cell extracts for CFPS reactions were prepared from BL21 Star (DE3) (Thermo Fisher, C601003) as previously described *(89)*.

CFPS reactions were assembled as previously described, except for the DNA template *(89)*. Unpurified linear expression templates in PCR reaction buffer were added to the CFPS reaction at 6.66% v/v to drive protein synthesis. CFPS reactions were run in 24 well plates at 30°C for 14 to 20 hours. Reactions of 100-200 µL were run in 24-well plates to produce protein for AlphaLISA off-rate screening (Falcon, 351147), and purified using StrepTactinXT spin columns (IBA, 2-4151-000) following manufacturer instructions. Post purification, proteins were extensively dialyzed (Thermo Fisher Scientific, 69552) into 50 mM HEPES pH 7.4, 150 mM NaCl.

Two mL reactions, to produce protein for ELISA, neutralization, and VSV escape studies, were run in 6-well plates (Costar, 3736) and purified using gravity flow using StrepTactinXT Superflow high capacity resin (IBA, 2-4030-010). CFPS reactions were incubated with resin on an end-over-end rotator for 30 minutes at room temperature. The resin was spun down at 2,500 x g for 2 minutes and the supernatant was removed. Resin was resuspended in Buffer W (IBA, 2-1003-100) and loaded onto a gravity flow column (BioRad, 7321010). Resin was washed with 20X resin volumes of Buffer W and proteins were eluted by the addition of Buffer BXT (IBA, 2-1042-025). Following purification, EDTA and CHAPS detergent were added to a concentration of 10 mM and 4 mg/mL, respectively to aid in the removal of endotoxin by dialysis *(90)*. Proteins were dialyzed (Thermo Fisher Scientific, 87724) into 1L of endotoxin-free 1x phosphate-buffered saline (PBS, Thermo Fisher Scientific, SH30256LS) in a 4 L glass beaker. Glass beakers were baked at 240°C for 12 hours to degrade endotoxin prior to use. Samples were dialyzed at 4°C for at least 6 hours. At least two additional rounds of dialysis with the addition of EDTA and CHAPS to concentrations of 10 mM and 4 mg/mL, respectively, were performed to remove endotoxin. Endotoxin was quantified using the Pierce Chromogenic Quant Kit (Thermo Fisher Scientific, A39553) and samples with less than 10 EU/mL were used for cell-based assays.

Expression and purification of competitor proteins for AlphaLISA experiments

Proteins used as competitors in the AlphaLISA off-rate screen were cloned into pET28a, transformed into BL21 Star DE3 (Thermo Fisher Scientific,C601003), plated on LB agar, and cultured overnight at 37°C. One L of Overnight Express TB (Thermo Fisher Scientific,

71491-4) was inoculated by scraping all colonies on a transformation plate and cultured at 37°C in 2.5 L tunair flasks (IBI Scientific, SS-8003) at 220 rpm overnight. Cells were harvested, resuspended at a ratio of 1 g cell mass to 4 mL resuspension buffer (50 mM HEPES pH 7.5, 500 mM NaCl, 1X HALT protease inhibitor without EDTA (Thermo Fisher Scientific, 78429), 1 mg/mL lysozyme, 62.5 U/mL cell suspension of benzonase (Sigma Aldrich, E1014-25KU)) and lysed using an Avestin B15 homogenizer at 21,000 PSI. Lysate was spun down 14,000 x *g* for 10 minutes and the clarified supernatant was incubated with Ni-NTA Agarose (Qiagen, 30230) for 60 minutes on an end-over-end shaker. Resin was spun down 2,500 x *g* for 2 min, supernatant removed, resuspended in wash buffer (50 mM HEPES pH 7.5, 500 mM NaCl, 50 mM Imidazole), loaded on a gravity flow column, and subsequently washed with 20X resin volumes of wash buffer. Protein was eluted using elution buffer (50 mM HEPES pH 7.5, 500 mM NaCl, 500 mM Imidazole) and exchanged into 50 mM HEPES pH 7.4, 150 mM NaCl using PD-10 desalting columns (Cytiva, 17-0851-01).

His tags were removed with cleavage by ProTEV Plus (Promega, V6102). Prior to cleavage, 10% v/v glycerol was added to the protein. ProTEV Plus was added to a concentration of 0.5 U/µg purified protein and dithiothreitol (DTT) was added to a concentration of 1 mM. Cleavage reactions were carried out at 30°C for 4 hours. Free his tag and ProTEV Plus were removed by incubating with Ni-NTA Agarose for 1 hour at 4°C and collecting the supernatant. Proteins were subsequently concentrated to greater than 1mg/mL (Millipore, UFC800396). His tag removal was validated by SDS PAGE and the AlphaScreen Histidine (Nickel Chelate) Detection Kit (Perkin Elmer, 6760619C).

Expression and purification of SARS-CoV-2 S proteins

S2P *(1, 50, 91, 92)*, S6P *(42)*, and S6P variants were produced in Expi293F cells (Thermo Fisher Scientific, A14527) grown in suspension using Expi293F expression medium (Life Technologies) at 33°C, 70% humidity, 8% CO2 rotating at 150 rpm. The cultures were transfected using PEI-MAX (Polyscience) with cells grown to a density of 3 million cells per mL and cultivated for 3 days. Supernatants were clarified by centrifugation (5 minutes at 4000 rcf), addition of poly(diallyldimethylammonium chloride) (PDADMAC) solution to a final concentration of 0.0375% (Sigma Aldrich, #409014), and a second centrifugation (5 minutes at 4000 rcf).

Proteins were purified from clarified supernatants through a batch bind method where each clarified supernatant was supplemented with 1 M Tris-HCl pH 8.0 to a final concentration of 45 mM and 5 M NaCl to a final concentration of about 310 mM. Talon cobalt affinity resin (Takara) was added to the treated supernatants and allowed to incubate for 15 minutes with gentle shaking. Resin was collected using vacuum filtration with a 0.2 µm filter and transferred to a gravity column. The resin was washed with 20 mM Tris pH 8.0, 300 mM NaCl, and the protein was eluted with 3 column volumes of 20 mM Tris pH 8.0, 300 mM NaCl, 300 mM imidazole. The batch bind process was then repeated and the first and second elutions were combined. SDS-PAGE was used to assess purity. Immobilized metal affinity chromatography (IMAC) elutions were concentrated to about 1 mg/mL and dialyzed three times into 50 mM Tris pH 8, 150 mM NaCl, 0.25% L-Histidine in a hydrated 10K molecular weight cutoff dialysis cassette

(Thermo Fisher Scientific). Due to inherent instability, S2P was immediately flash frozen and stored at −80°C.

Competition-based off-rate screening by AlphaLISA

AlphaLISA reactions were carried out in 50 mM HEPES pH 7.4, 150 mM NaCl, 1 mg/mL BSA, and 0.015% v/v TritonX-100 (hereafter referred to as Alpha buffer). All components were dispensed using an Echo 525 liquid handler from a 384-Well Polypropylene 2.0 Plus microplate (Labcyte, PPL-0200) using the 384PP_Plus_GPSA fluid type. All AlphaLISA reactions were performed in a ProxiPlate-384 Plus (Perkin Elmer, 6008280). AlphaLISA StrepTactin donor beads, to capture StrepII or TwinStrep-tagged minibinder variants, (Perkin Elmer, AS106) and AlphaLISA Anti-6x-his, to capture 6xhis-tagged S6P or RBD, (Perkin Elmer, AL178C) were combined to prepare a 4X stock in Alpha buffer immediately prior to use and added to the proteins to yield a concentration of 0.08 mg/mL donor beads and 0.02 mg/mL acceptor beads in the final reaction.

Multivalency screening experiments were carried out at a final concentration of 2.5 nM S6P, 2.5 nM minibinder variant, and 250 nM of the specified untagged competitor in Alpha buffer. First, the minibinder variant and S6P were diluted to 1.33x final concentration (adjusted for the later addition of competitor) in 120 µL in a 384-Well Polypropylene 2.0 Plus plate and sealed (BioRad, MSB1001). Samples were allowed to fully associate for 12 to 16 hours at 20°C. Next, conditions were split in half and the same volume of either buffer or 250 nM (100x molar excess) untagged competitor were added to achieve 1.33x final concentration of all components (competitor was previously concentrated to achieve

less than 5% volume change at this step). Samples were then incubated for the specified time, with replicates measured by dispensing 1.5 µL of each condition and 0.5 µL of 4X Alpha bead stock using the Echo 525 liquid handler. Plates were immediately spun down following the dispense and sealed (BioRad, MSB1001). Reactions were incubated with beads for 1 hour for the 2-hour dissociation time points and up to 2 hours for longer dissociation time points before measurement (bead incubation time was included in the specified timepoints). AlphaLISA measurements were taken on a Tecan Infinite M1000 Pro using the AlphaLISA filter with an excitation time of 100 ms, an integration time of 300 ms and a settle time 20 ms. Prior to measurement, plates were allowed to equilibrate inside the instrument for 10 minutes. Fraction of protein bound was determined by subtracting the average background bead signal and then dividing the plus competitor by the minus competitor condition. Values below zero after background subtraction were set to zero. Conditions with signal in the no competitor condition within 3 standard deviations (s.d.) of the background were set to zero. After normalization, conditions with values less than 0.01 were set to zero to account for the typical max dynamic range of the measurement. Prism 9 (GraphPad) was used to plot the data.

Monovalent optimization experiments were performed in the same manner as multivalency screening experiments. S6P and RBD comparison experiments were carried out in the same manner as the multivalency screening experiments except for concentrations of 5 nM of S6P or RBD (Sino Biological, 40592-V08H), 5 nM minibinder variant, and 500 nM (100x molar excess) of the specified untagged competitor in the final reactions. In S6P variant experiments, reactions were carried out in the same manner as

the multivalency screening experiments.

Negative stain electron microscopy

S6P was produced in HEK293F cells grown in suspension using FreeStyle 293 expression medium (Life Technologies) at 37˚C in a humidified 8% CO2 incubator rotating at 130 rpm. The cultures were transfected using polyethylenimine (PEI) (9 µg/mL) with cells grown to a density of 2.5 million cells per mL and cultivated for 3 days. The supernatants were harvested, and cells resuspended for another 3 days, yielding two harvests. Spike proteins were purified from clarified supernatants using a 5 mL Cobalt affinity column (Cytiva, HiTrap TALON crude), concentrated and flash frozen in a buffer containing 20 mM Tris pH 8.0 and 150 mM NaCl prior to analysis.

10 µM SARS-CoV-2 Spike was incubated with 13 µM minibinders for 1 hour at room temperature. Samples were diluted to 0.01 mg/mL immediately prior to adsorption to glow-discharged carbon-coated copper grids for about 30 seconds prior to a 2% uranyl formate staining. Micrographs were recorded using the Leginon on a 120 KV FEI Tecnai G2 Spirit with a Gatan Ultrascan 4000 4k x 4k CCD camera at 67,000 nominal magnification. The defocus ranged from -1.0 to -2.0 µm and the pixel size was 1.6 Å.

Cryo-electron microscopy

S6P *(42)* at 1.2 mg/mL was incubated with 1.2 fold molar excess of recombinantly purified TRI2-2, FUS31-G10, or FUS231-P24 at 4°C before application onto a freshly glow discharged 2.0/2.0 UltrAuFoil grid (200 mesh) *(93)*. Plunge freezing used a vitrobot

MarkIV (Thermo Fisher Scientific) using a blot force of 0 and 6.5 second blot time at 100% humidity and 23°C.

For the S6P/TRI2-2 data set, data were acquired using an FEI Titan Krios transmission electron microscope operated at 300 kV and equipped with a Gatan K3 direct detector and Gatan Quantum GIF energy filter, operated in zero-loss mode with a slit width of 20 eV. Automated data collection was carried out using Leginon *(94)* at a nominal magnification of 105,000x with a pixel size of 0.4215 Å. The dose rate was adjusted to 15 counts/pixel/s, and each movie was acquired in super-resolution mode fractionated in 75 frames of 40 ms. 5,991 micrographs were collected with a defocus range between -0.5 and -2.5 μm. Movie frame alignment, estimation of the microscope contrast-transfer function parameters, particle picking, and extraction were carried out using Warp *(95)* (fig. S8 and S9).

For the S6P/FUS31-G10 data set, data were acquired on an FEI Titan Krios transmission electron microscope operated at 300 kV equipped with a Gatan K2 Summit direct detector and Gatan Quantum GIF energy filter, operated in zero-loss mode with a slit width of 20 eV. Automated data collection was carried out using Leginon *(94)* at a nominal magnification of 130,000x with a pixel size of 0.525 Å. The dose rate was adjusted to 8 counts/pixel/s, and each movie was acquired in counting mode fractionated in 50 frames of 200 ms. 1000 micrographs were collected in a single session with a defocus range between -0.5 and -2.5 μm (fig. S10).

For the S6P/FUS231-P24 data set, data were acquired on an FEI Glacios transmission electron microscope operated at 200 kV equipped with a Gatan K2 Summit direct detector. Automated data collection was carried out using Leginon *(94)* at a nominal magnification of 36,000x with a pixel size of 1.16 Å. The dose rate was adjusted to 8 counts/pixel/s, and each movie was acquired in counting mode fractionated in 50 frames of 200 ms. 1,663 micrographs were collected in a single session with a defocus range between -0.5 and -2.5 μm (fig. S11).

For the S6P/TRI2-2, S6P/FUS31-G10, and S6P/FUS231-P24 datasets, two rounds of reference-free 2D classification were performed using Cryo-EM Single Particle Ab-Initio Reconstruction and Classification (CryoSPARC) *(96)* to select well-defined particle images. These selected particles were subjected to two rounds of 3D classification with 50 iterations each (angular sampling 7.5˚ for 25 iterations and 1.8˚ with local search for 25 iterations), using our previously reported closed SARS-CoV-2 S structure as initial model (PDB 6VXX) in Relion *(97)*. 3D refinements were carried out using non-uniform refinement *(98)* along with per-particle defocus refinement in CryoSPARC. Selected particle images were subjected to the Bayesian polishing procedure implemented in Relion3.0 *(99)* before performing another round of non-uniform refinement in CryoSPARC followed by per-particle defocus refinement and a third round of non-uniform refinement. To further improve the density of the TRI2-2, the particles were then subjected to focus 3D classification without refining angles and shifts using a soft mask comprising the RBD and TRI2-2 region with a tau value of 60 in Relion. Particles belonging to classes with the best resolved local density were selected and subject to local refinement using

CryoSPARC. Local resolution estimation, filtering, and sharpening were carried out using CryoSPARC. Reported resolutions are based on the gold-standard Fourier shell correlation (FSC) of 0.143 criterion and Fourier shell correlation curves were corrected for the effects of soft masking by high-resolution noise substitution *(100, 101)*. UCSF Chimera *(102)* and Coot *(103)* were used to fit atomic models into the cryoEM maps. Spike-RBD/TRI2-2 model was refined and relaxed using Rosetta *(104, 105)* using sharpened and unsharpened maps (table S3, fig. S9 to S11).

BRET sensor for SARS-CoV-2 S detection

Synergy Neo2 plate reader (BioTek) was used for all luminescent assays. Ten μL of 1 nM teluc-FUS231-P12-mCyRFP3, 10 μL of serial diluted 10X S2P protein (final concentrations range from 1.85 nM to 0.85 pM), and 30 μL of buffer (25 mM Tris, pH8, 50mM NaCl) were mixed and incubated for 10 minutes at room temperature. Diphenylterazine stock solution was prepared as previously described *(51)*. Then, 50 μL of diluted diphenylterazine solution (60 μM) was added to each well. The luminescence spectra were collected under the monochromator mode with 0.1 s integration and 5 nm increments from 400 to 750 nm. The luminescent image was taken by an iPhone 8 camera. To record the emission ratio, luminescence signals were acquired by a filter mode with a two-channel cube (470/40 nm and 590/35 nm). The emission ratios were calculated from 590 nm:470 nm channels directly. The linear region of ratiometric responses was extracted and a linear regression curve was plotted, which was used to derive the s.d. of the response and the slope of the calibration curve (S). The limit of detection was determined as 3 s.d. above background signal. The limit of detection of the

sensor was compared with the performance of commercial lateral flow nucleocapsid (N) protein antigen tests in Corman *et al. (106)* on purified N protein assuming a 10:1 molar ratio of N protein to S trimer per virion *(107)*.

BRET sensor protein sequence [His-TEV][teluc][FUS231-P12][mCyRFP3]:

[MGSHHHHHHGSGSENLYFQGSG][VFTLEDFVGDWRQTAGYNLSQVLEQGGVSSLF
QNLGVSVTPIQRIVLSGENGLKIDIHVIIPYEGLSGDQMGQIEKIFKVVYPVDNHHFKVIL
HYGTLVIDGVTPNMIDYFGRPYEGIAVFDGKKITVTGTLWNGNKIIDERLINPDGSLLFR
VTINGVTGWRLHERILASGSSG][ELEEQVMHVLDQVSELAHELLHKLTGEELERAAYF
NWWATEMMLELIKSDDEREIREIEEEAARILEHLEELARTGGASPAAPAPGGNLDELH
MQMTDLVYEALHFAKDEEFQKHVFQLFEKATKAYKNKDRQKLEKVVEELKELLERLLS
GGASPAAPAPGGDKENVLQKIYEIMKELERLGHAEASMQVSDLIYEFMKTKDENLLEE
AERLLEEVKR][GEELIKENMRSKLYLEGSVNGHQFKCTHEGEGKPYEGKQTARIKVVE
GGPLPFAFDILATMFMYGSKVFIKYPADLPDYFKQSFPEGFTWERVMVFEDGGVLTAT
QDTSLQDGELIYNVKLRGVNFPANGPVMQKKTLGWEPSTETMTPADGGLEGRCDKVL
KLVGGGHLHVNFKTTYKSKKPVKMPGVHYVDRRLERIKEADNETYVEQYEHAVARYS
NLGGGMDELYK]

BRET Sensor DNA sequence:

atgggcagccatcatcatcaccatcatggtagcggcagcgaaaacttgtattttcaggggagcggcgtgtttaccctggaa
gattttgtgggcgattggcgccagaccgcgggctataacctgagccaggttctggaacagggtggtgtgagcagcctgttt
cagaatctgggcgtgagcgttaccccgatccagcgcattgtactgtctggtgaaaacggcctgaaaattgatatccatgtga
ttattccgtatgaaggcctgagcggcgatcagatgggccagattgaaaagatctttaaagtggtgtatccggtggataacca

tcatttcaaagtgattctgcattatggcaccctggttattgatggggttacgccgaacatgattgattattttggccgcccgtacg
aaggcattgccgtgtttgatggcaaaaagattaccgtgaccggcacgctgtggaacggtaacaaaattattgacgaacgc
ctgattaacccggatggctctctgctgtttcgcgtgaccattaatggcgtcaccggctggcgtctgcacgaacgtatcttggca
agcggtagcagtggtgaactggaagaacaagtgatgcatgtgctggatcaagtgagcgaactggcccatgagctgctgc
ataaactgaccggtgaagaacttgaacgcgcggcgtattttaactggtgggcaaccgaaatgatgctggaactgatcaaa
agcgatgatgaacgtgaaattcgcgaaattgaagaagaggcggcccgtattctcgaacatctggaagaattggcgaga
acaggtggtgcttcacctgctgctcctgcgccaggtggtaatttagatgaattgcacatgcagatgaccgatctggtttatga
agcgctgcattttgccaaagatgaagaatttcagaaacatgttttccagctgtttgaaaaagcgacgaaagcgtacaaaaa
caaagaccgccagaaattggaaaaagttgtcgaagaactgaaagaattactggaacgtctgttgagcggtggagcaag
cccagctgcaccagctcctggtggtgacaaagaaaacgtcctccagaaaatttacgaaattatgaaagagctggaaag
gctgggccatgctgaagcgagtatgcaggttagcgatctgatctatgaattcatgaaaaccaaagacgaaaacctgttgg
aagaagccgaacgtttgttagaagaagtgaaacgcggcgaagaattaattaaagaaaacatgcgtagcaaactgtattt
ggaaggcagcgtgaacggccatcagtttaaatgcacccatgaaggtgaaggcaaaccttacgaaggtaaacagacgg
cgcgcattaaagtcgtggaaggtggcccgctgccgtttgcgtttgatattctggcgaccatgtttatgtatggcagcaaagtgt
ttattaaatatccggcggatctgccggattatttcaaacagagctttccggaaggctttacctgggaacgcgtgatggtgttcg
aagatggcggtgtgctgaccgcgacccaggataccagcctgcaagatggggaactcatttataacgttaaactgcgcggt
gttaacttcccggcgaacggtccggttatgcagaagaaaaccctgggttgggaaccgagcaccgaaaccatgaccccg
gcagatggtggtttagaaggccgctgcgataaagtgctgaaactggttggcggcggccacctgcatgtcaactttaaaac
cacgtataaaagcaaaaagccggttaaaatgccgggcgtgcactatgtggatcgccgtctggaacgcatcaaagaagc
ggataacgaaacctatgttgaacagtatgaacacgccgtggcgcgttatagcaacctcggtggtggcatggatgaactgt
acaaataa

Minibinder/RBD interaction kinetics through bio-layer interferometry (BLI)

The Octet HTX instrument was used to determine affinities of minibinders to monovalent soluble RBD (sol-RBD) (obtained from the Institute of Protein Design at University of Washington). Prior to sol-RBD affinity measurements, Amine Reactive 2nd Generation (AR2G) biosensors (Sartorius, #18-5094) were pre-hydrated for 10 minutes in water before being activated using (1-ethyl-3-(3-dimethylaminopropyl)carbodiimide hydrochloride)/N-hydroxysuccinimide (EDC/NHS) chemistry (solution containing 20 mM EDC and 10 mM NHS in ddH2O) for 5 min. Sol-RBD in 10 mM Acetate buffer pH 5.0 was reacted with activated AR2G biosensors for 10 minutes and quenched for 5 minutes in 1M ethanolamine pH 8.5. Sol-RBD linked AR2G biosensors were equilibrated for 60 s in running buffer (10 mM Tris, 150 mM NaCl, 1 mM CaCl2, 0.1 mg/mL bovine serum albumin (BSA), and 0.1% Triton-X 100, pH7.4) before measuring association of minibinders at concentrations ranging from 20 nM to 0.25 nM for 10 minutes, followed by 20 minutes of dissociation in running buffer. Minibinders were prepared in the running buffer. For data evaluation, the ForteBio Data Analysis v11.0 software was used. The kinetic rate constants, association rate constant (k_a, M-1s-1), dissociation rate constant (k_d, s-1), and the equilibrium rate constant (K_D, M) were determined by using a 1:1 Langmuir model. Following standard best practice, 5% dissociation during the experiment was used as a threshold to determine the lower limit of detection for the dissociation rate constant *(108)*.

Multivalent Minibinder/S6P variant interaction kinetics using surface plasmon resonance (SPR)

The Carterra LSA instrument was used to perform high throughput (HT)-SPR of minibinder affinity to S protein variants. To prepare the surfaces, the Single Flow Channel

(SFC) and 96-Print Head (96PH) were primed with running buffer (Hepes buffered Steinberg's Solution (HBS-T); 50 mM HEPES pH 7.5, 150 mM NaCl, 0.1% Tween 20). The capture surface was prepared in the 96PH by standard amine-coupling. A HC30-M chip (Carterra LSA cat# 4279) was activated with a 10-minute injection of freshly prepared 1:1:1 (v/v/v) mixture of 0.4 M EDC + 0.1 M NHS + 0.1 M MES pH 5.5. S protein trimers were diluted to 12.5 μg/ml in 10 mM sodium acetate pH 4.5 (Carterra, #3628) and coupled for 20 minutes. Excess reactive esters were blocked with a 7-minute injection of 1 M ethanolamine HCl pH 8.5 (Carterra, #3626). Final coupling amounts were greater than 1000 response units (RU).

Minibinders were prepared in HBS-T buffer, at a three-fold dilution series for 6 points starting at 20nM. Association was for 20 minutes, with a 60-minute dissociation time. Samples were injected in ascending concentration without any regeneration. The data was double referenced in that both a local reference and a zero nanomolar analyte concentration (buffer) were subtracted. The double-referenced data were fit globally to a 1:1 Langmuir binding model in Carterra's Kinetic tool, allowing each spot its own K_a and K_d value to determine K_D. Following standard best practice, 5% dissociation during the experiment was used as a threshold to determine the lower limit of detection for the dissociation rate constant *(108)*.

Competition ELISA of minibinders and SARS-CoV-2 S6P variants for immobilized hACE2-Fc

A 0.003 mg/mL solution of hACE2-Fc in 20mM Tris pH 8 and 100mM NaCl was

immobilized to a Maxisorp 384-well plate (Thermo Fisher Scientific 464718) overnight at 4°C. Plates were slapped dry and blocked with Blocker Casein in Tris-buffered saline (TBS, Thermo Fisher Scientific 37532) for one hour at 37°C. 20nM mini binders were serially diluted 1:3 in avi-tagged prefusion-stabilized SARS-CoV-2 S6P *(42)* variants at their EC_{50} concentrations of 1.2nM for WT, 6.3nM for E406W, 1.7nM for K417N, 0.5nM for Y453F, 0.6nM for Y453R, 1.3nM for L455F, 1.1nM for F456L, 0.1nM for E484K, 0.2nM for N501Y, 0.3nM for B.1.1.7, 0.2nM for B.1.351, or 0.2nM for P.1 and incubated for 30 minutes on a non-binding plate (Greiner 781901) at 37°C. The plates with blocking buffer were slapped dry and the pre-incubated mini binders and spikes were added. Plates were incubated for 1 hour at 37°C then washed 4x with TBS with 0.1% Tween 20 (TBS-T) using a 405 TS Microplate Washer (BioTek) followed by addition of 30 µL avi-tag pAb (GenScript A00674) at 0.2 µg/mL. Plates were incubated for 1 hour at 37°C then washed 4x with TBS-T using a 405 TS Microplate Washer (BioTek) followed by addition of 30 µL 1:2000 Goat anti-Rabbit horseradish peroxidase (HRP, Invitrogen 656120) and a 1-hour incubation at 37°C. Plates were washed 4x and 3,3',5,5'-Tetramethylbenzidine (TMB) Microwell Peroxidase (Seracare 5120-0083) was added. The reaction was quenched after 2 to 3 minutes with 1 N HCl and the A450 of each well was read using a BioTek plate reader (BioTek). Data were plotted and fit in Prism (GraphPad) using nonlinear regression sigmoidal, 4 parameter logistic (4PL) curve, where X is log(concentration) to determine IC_{50} values from curve fits. IC_{50} values less than two-fold below the concentration of S6P in that condition were not considered different from two-fold below the concentration of S6P.

Pseudovirus production

HIV-based pseudotypes were prepared as previously described *(74)*. Briefly, HEK293T cells were co-transfected using Lipofectamine 2000 (Life Technologies) with an S-encoding plasmid with full suite of variant mutations, an HIV Gag-Pol, Tat, Rev1B packaging construct, and the HIV transfer vector encoding a luciferase reporter according to the manufacturer's instructions. Cells were washed 3x with Opti-MEM and incubated for 5 hours at 37°C with transfection medium. DMEM containing 10% FBS was added for 60 hours. The supernatants were harvested by spinning at 2,500 x g, filtered through a 0.45 μm filter, concentrated with a 100 kDa membrane for 10 minutes at 2,500 x *g* and then aliquoted and stored at −80°C.

Pseudovirus neutralization

HEK-hACE2 cells were cultured in DMEM with 10% FBS (HyClone) and 1% penicillin-streptomycin with 8% CO_2 in a 37°C incubator (Thermo Fisher Scientific). One day prior to infection, 40 μL of poly-lysine (Sigma Aldrich) was placed into 96-well plates and incubated with rotation for 5 minutes. Poly-lysine was removed, plates were dried for 5 minutes, then washed 1x with water prior to plating with 40,000 cells. The following day, cells were checked to be at 80% confluence. In an 80 μL final volume, minibinders were serially diluted in DMEM 1:3 starting at 100 nM. Pseudovirus was added 1:1 to the diluted minibinders and allowed to incubate for 30 to-60 minutes at room temperature. After incubation, the mixture of minibinder and virus was added to the cells at 37°C and allowed to incubate for 2 hours. Post-infection, 160 μL of DMEM with 20% FBS and 2% penicillin-streptomycin was added. After 48 hours, 40 μL/well of One-Glo-EX substrate (Promega)

was added to the cells and incubated in the dark for 5 to 10 minutes prior reading on a BioTek plate reader. Measurements were done in at least duplicate. Relative luciferase units were plotted and normalized in Prism (GraphPad). Nonlinear regression of log(inhibitor) versus normalized response was used to determine IC_{50} values from curve fits.

SARS-CoV-2 neutralization

Serial dilutions of minibinders were incubated with 10^2 FFU of SARS-CoV-2 for 1 hour at 37°C. Binder-virus complexes were added to Vero-hACE2-TMPRSS2 cell monolayers in 96-well plates and incubated at 37°C for 1 hour. Subsequently, cells were overlaid with 1% (w/v) methylcellulose in MEM supplemented with 2% FBS. Plates were harvested 20 to 24 hours later by removing overlays and fixed with 4% paraformaldehyde (PFA) in PBS for 20 minutes at room temperature. Plates were washed and sequentially incubated with an oligoclonal pool of SARS2-2, SARS2-11, SARS2-16, SARS2-31, SARS2-38, SARS2-57, and SARS2-71 anti-spike protein antibodies *(23)* and HRP-conjugated goat anti-mouse IgG in PBS supplemented with 0.1% saponin and 0.1% BSA. SARS-CoV-2-infected cell foci were visualized using TrueBlue peroxidase substrate (KPL) and quantitated on an ImmunoSpot microanalyzer (Cellular Technologies). Data were processed using GraphPad Prism 8.0.

Kidney organoid differentiation and infection with SARS-CoV-2

Kidney organoids were differentiated from H9 human embryonic stem cells (WiCell, WA09) for 21 days prior to infection in adherent, thin-layer Matrigel sandwich cultures

induced for 36 hours with CHIR99021 as described previously *(57)*. SARS-CoV-2 strain B.1.351-HV001 containing E484K/N501Y/D614G mutations along with furin cleavage site point mutation was obtained directly from B.1.351 clinical isolates. All experiments using live viruses were performed at Biosafety Level 3 (BSL-3) facilities at the University of Washington in compliance with BSL-3 laboratory safety protocols (CDC BMBL 5th ed.) and the recent CDC guidelines for handling SARS-CoV-2. Virus stocks were generated and titrated using plaque forming assays in Vero CCL-81 cells (ATCC Cat# CCL-81, RRID: CVCL_0059) (a gift from the United States Army Medical Research Institute of Infectious Diseases (USAMRIID)). Minibinders FUS231-10GS, TRI2-2, and MON1 at 0.3 µM were diluted in serum-free DMEM and pre-incubated with virus (10 multiplicity of infection) for 1 hour at 37°C. The virus minibinder mix was then added to kidney organoids for 1 hour at 37°C. After 1 hour, kidney organoids were washed with 1X PBS and fresh organoid growth media (Advanced RPMI-1640 + 1X Glutamax + 1X B27 Supplement, Thermo Fisher Scientific) was added and incubated for 72 hours. Supernatants were harvested from the infected organoids for plaque forming assays and cells were lysed using Trizol RNA extract for gene expression analysis.

Kidney organoid plaque assay

Vero CCL-81 cells (ATCC Cat# CCL-81, RRID: CVCL_0059) were plated at 80% confluency, washed with 1X PBS, and incubated with serially diluted supernatant from infected organoids for 1 hour at 37°C. Cells were then overlaid with a 1:1 mixture of 1.8% cellulose in water:2X DMEM supplemented with 4% heat-inactivated FBS, L- glutamine, 1X antibiotic-antimycotic (Gibco), and 220 mg/mL sodium pyruvate was layered on top of

the cells and incubated for 48 hours at 37°C. Cells were then fixed using 10% formaldehyde and incubated for 30 minutes at room temperature. Overlay was removed carefully and cells were stained with 0.5% crystal violet solution in 20% ethanol. Plaques were counted and the virus titer in the original sample was calculated as plaque-forming units per mL (PFU/mL). Data was analyzed using GraphPad Prism 8.0. Limit of detection for the plaque assay was determined by calculating the concentration (PFU/mL) corresponding to a single observed plaque. Conditions where zero plaques were observed were set to the value of the limit of detection.

Kidney organoid gene expression analysis

RNA from infected kidney organoids was harvested using Trizol reagent. cDNA was generated using iScript cDNA Synthesis Kit (catalog #1708890, Bio-rad). qRT-PCR was performed using SYBR green master mix (catalog #4309155, Applied Biosystems) and the following primers: SARS-CoV2-E F (GAACCGACGACGACTACTAGC), SARS-CoV2-E R (ATTGCAGCAGTACGCACACA), β-ACTIN F (GCAAAGACCTGTACGCCAACA), β-ACTIN R (ACACGGAGTACTTGCGCTCAG).

Selection of escape mutants in SARS-CoV-2 S using VSV-SARS-CoV-2 chimera

VSV-SARS-CoV-2 chimera (S from Wuhan-Hu-1 D614G strain) was used to select for SARS-CoV-2 S minibinder resistant mutants as described previously for selection of SARS-CoV-2 monoclonal antibody escape mutants *(6, 21)*. Briefly, VSV-SARS-CoV-2 chimera virus was allowed to infect Vero CCL-81 cells for 1 hour at 37 °C. For the no inhibitor control, approximately 40 PFU of VSV-SARS-CoV-2 chimera were added per

well. For the inhibitor containing conditions, 10^6 PFU of VSV-SARS-CoV-2 chimera were added per well. Following infection, inhibitor protein (minibinder or monoclonal antibody) was added to the culture medium and incubated for 72 hours to allow for the replication and spreading of resistant viruses. Neutralization resistant mutants were recovered by plaque isolation on Vero CCL-81 cells with the indicated minibinder in the overlay. The concentration of minibinder in the overlay was determined by neutralization assays at a multiplicity of infection (MOI) of 100. The concentration of 2B04, FUS231-P12 and TRI2-2 added in the overlay was 102 ng/ml, 58 ng/ml and 58 ng/ml respectively. Escape clones were plaque-purified on Vero cells in the presence of peptide binder or monoclonal antibody 2B04 respectively, and plaques in agarose plugs were amplified on MA104 cells with the peptide binder present in the medium. Viral stocks were amplified on MA104 cells at an MOI of 0.01 in Medium 199 containing 2% FBS and 20 mM HEPES pH 7.7 (Millipore Sigma) at 34°C. Viral supernatants were harvested upon extensive cytopathic effect and clarified of cell debris by centrifugation at 1,000 x g for 5 minutes. Aliquots were maintained at -80°C. Viral RNA was extracted from VSV-SARS-CoV-2 mutant viruses using an RNeasy Mini kit (Qiagen), and S was amplified using OneStep RT-PCR Kit (Qiagen). The mutations present in 2B04 escape variants were identified by Sanger sequencing (GENEWIZ).

To determine the diversity present in the pool of viral particles used for the escape selection, the Pedel-AA *(109)* web tool for predicting protein diversity in randomized libraries was used (http://guinevere.otago.ac.nz/aef/STATS/index.html). Upper (10^{-3} errors per nucleotide) and lower (10^{-4} errors per nucleotide) estimates of the VSV RNA

polymerase L error rate *(61)* were evaluated separately to provide a plausible range of library diversities. Only the continuous amino acid sequence that contacts the minibinders (Genbank accession# MN908947.3, surface glycoprotein residues 400 to 508) within the SARS-CoV-2 RBD was considered. The library diversity was calculated using an equal probability of mutating from any nucleotide to any other, a zero probability of insertions and deletions, and a Poisson distribution to estimate the mean number of mutations per sequence.

Protein production for animal studies

Protein was produced by fermentation in the *E. coli* strain BL21 pLysS (Thermo Fisher Scientific, C602003) strain using pET vectors induced with Isopropyl β- d-1-thiogalactopyranoside (IPTG). The 6xHis tagged proteins were purified from clarified cell lysates by immobilized metal chelate chromatography (IMAC, Ni-NTA resin) and step eluted with 300 mM imidazole. Proteins were further purified by size exclusion chromatography using an S75 Increase Column into a final buffer of 20mM NaPO4 150mM NaCl pH 7.4. Proteins were analyzed by SDS-PAGE after heating 95°C without reducing agent DTT followed by Coomassie blue staining (molecular weight standards shown). Protein endotoxin amounts are less than 10 endotoxin units (E.U.)/mg.

Mouse studies

Animal studies were carried out in accordance with the recommendations in the Guide for the Care and Use of Laboratory Animals of the National Institutes of Health. The protocols were approved by the Institutional Animal Care and Use Committee at the

Washington University School of Medicine (assurance number A3381–01). Virus inoculations and intranasal minibinder treatments were performed under anesthesia that was induced and maintained with ketamine hydrochloride and xylazine, and all efforts were made to minimize animal suffering.

Heterozygous K18-hACE2 C57BL/6J female mice (strain: 2B6.Cg-Tg(K18-ACE2)2Prlmn/J) were obtained from The Jackson Laboratory. Animals were housed in groups and fed standard chow diets. For inoculation, 8-week-old mice were administered 10^3 PFU of the indicated SARS-CoV-2 strain in 50 µL total volume by the intranasal route. Briefly, mice were anesthetized and the virus inocula was slowly pipetted into both nostrils of each mouse. Following administration, animals were placed on their back and observed for 5 minutes to ensure aspiration into the lungs. This procedure was performed identically for administration of minibinders at D-1 or D+1 relative to virus inoculation. A previously described influenza minibinder was used as a negative control *(71)*.

Measurement of viral burden in mouse studies

Tissues were weighed and homogenized with zirconia beads in a MagNA Lyser instrument (Roche Life Science) in 1 mL of DMEM media supplemented with 2% heat inactivated FBS. Tissue homogenates were clarified by centrifugation at 10,000 rpm for 5 minutes and stored at −80°C. To measure viral RNA, RNA was extracted using the MagMax mirVana Total RNA isolation kit (Thermo Fisher Scientific) on a Kingfisher Flex extraction robot (Thermo Fisher Scientific). RNA was reverse transcribed and amplified using the TaqMan RNA-to-CT 1-Step Kit (Thermo Fisher Scientific). Reverse transcription

was performed at 48°C for 15 minutes followed by 2 minutes at 95°C. Amplification was accomplished over 50 cycles as follows: 95°C for 15 s and 60°C for 1 minute. Copies of SARS-CoV-2 *N* gene RNA in samples were determined using a previously published assay *(62, 110)*. Briefly, a TaqMan assay was designed to target a highly conserved region of the *N* gene (Forward primer: ATGCTGCAATCGTGCTACAA; Reverse primer: GACTGCCGCCTCTGCTC; Probe: /56-FAM/TCAAGGAAC/ZEN/AACATTGCCAA/3IABkFQ/). This region was included in an RNA standard to allow for copy number determination down to 10 copies per reaction. The reaction mixture contained final concentrations of primers and probe of 500 and 100 nM, respectively.

To measure viral titers, Vero-TMPRSS2-hACE2 cells were seeded at a density of 1×10^5 cells per well in 24-well tissue culture plates. The following day, medium was removed and replaced with 200 µL of material to be titrated diluted serially in DMEM supplemented with 2% FBS. One hour later, 1 mL of 1:1 DMEM:2% (w/v) methylcellulose overlay was added. Plates were incubated for 72 hours, then fixed with 4% PFA (final concentration) in PBS for 20 minutes. Plates were stained with 0.05% (w/v) crystal violet in 20% methanol and washed twice with distilled, deionized water.

Pharmacokinetics of TRI2-2 in mouse studies

For pharmacokinetic studies, 6-week-old C57BL/6J mice were administered TRI2-2 intranasally as described above for mouse protection studies. At the indicated time points post-administration, tissues were collected, weighed, and homogenized with zirconia

beads in a MagNA Lyser instrument (Roche Life Science) in 1 mL of DMEM supplemented with 2% heat inactivated FBS. Tissue homogenates were clarified by centrifugation at 10,000 rpm for 5 minutes and stored at −80 °C. To determine TRI2-2 binder concentrations in tissues, a competitive ELISA was performed in the same manner as previously reported *(26)*. Maxisorp 96-well plates (Thermo Fisher Scientific #44-2404-21) were coated with 4 ng/µL S6P in 1X TBS-T (20X TBS-T: Thermo Fisher Scientific #28360) for 1 hour at 37°C. Plates were washed four times with 300 µL of 1X TBS-T before blocking with 200 µL SuperBlock blocking buffer (Thermo Fisher Scientific #37515) for 1 hour at 37°C. Plates were washed four times with 300 µL of 1X TBS-T. One hundred µL 2 nM hACE2-Fc in 1X TBS-T was added to all wells. A volume of 16.7 µL of 300 nM LCB1v1.3 was added to the first row of columns 1 to 3 as a standard, and 16.7 µL of tissue sample was added to the first row of other columns. All samples were processed in triplicate. Samples and standards were serially diluted in a 1:3 fashion and then incubated for 1 hour at 37°C. Plates were washed four times with 300 µL of 1X TBS-T. Fifty µL of 1:5000 anti-hFc-HRP secondary antibody (Invitrogen #A18817) was added to each well and incubated for 1 hour at 37°C. Plates were washed four times with 300 µL of 1X TBS-T. One hundred µL TMB (Thermo Fisher Scientific #34028) was added to each well, and plates were placed on an orbital shaker for 30 seconds before addition of 100 µL of 1 N HCl to stop the reaction. Plates were read for absorbance at 450 nm on a SpectraMax Plus 384 plate reader. Data were analyzed by nonlinear regression in GraphPad Prism to obtain IC_{50} values. Sample TRI2-2 concentrations were determined by using the ratio of standard IC_{50}:standard concentration to sample IC_{50}:sample concentration.

Supplementary Figures

```
ACE2   STIEEQAKTFLDKFNHEAEDLFY
AHB2   -ELEEQVMHVLDQVSELAHELLH
```

Fig. S1. Sequence alignment of AHB2 and ACE2. The main interacting helix of AHB2 and ACE2 were aligned using the Clustal Omega tool *(111)*. Conserved residues between the two sequences are highlighted in cyan.

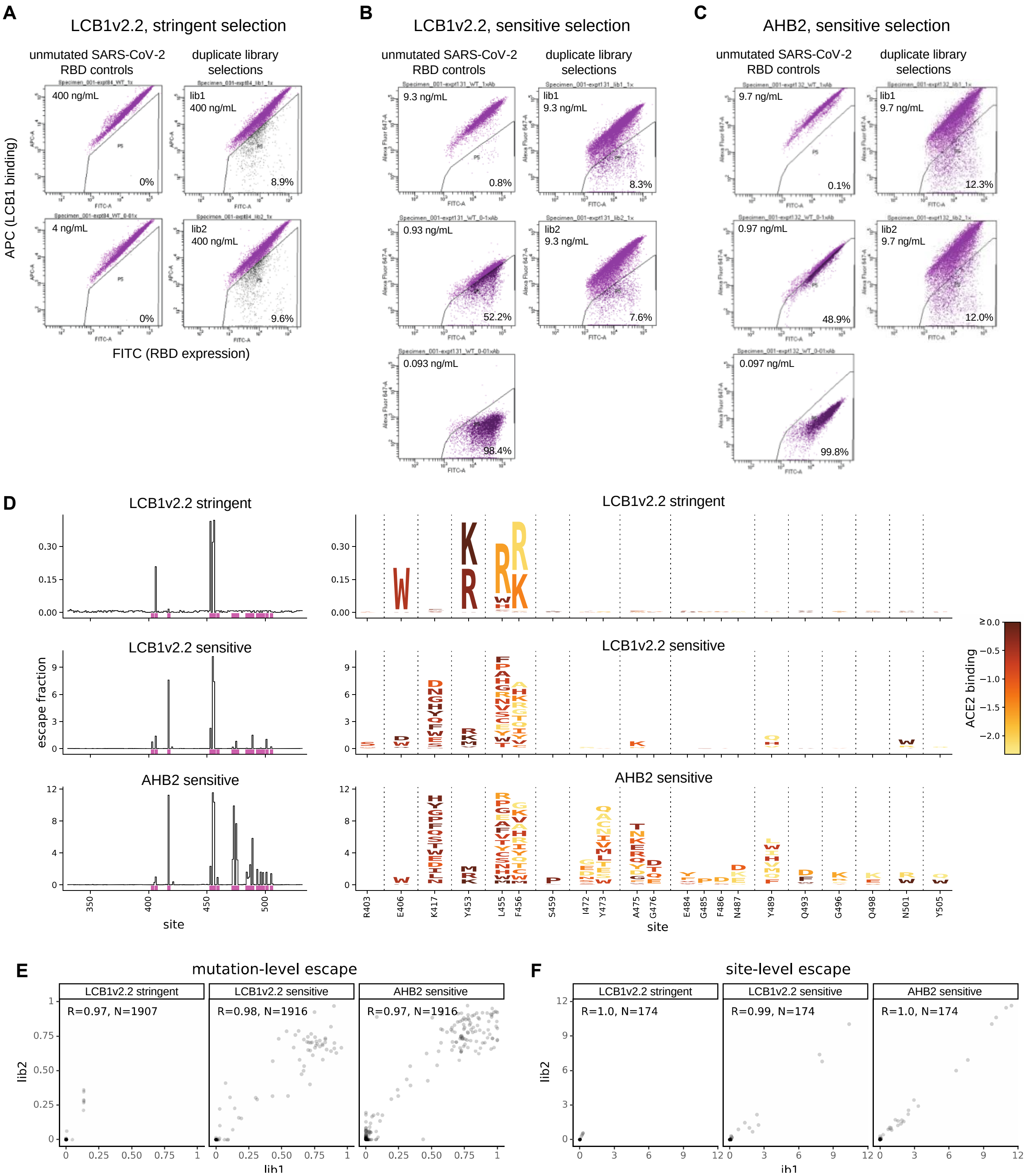

Fig. S2. Site saturation mutagenesis of RBD to predict MON1 (LCB1v2.2) and MON2 (AHB2) escape mutants. (A-C) FACS gates for deep mutational scanning selections. Duplicate yeast display libraries expressing all mutations tolerated in the SARS-CoV-2 RBD were labeled with LCB1_v2.2 **(A-B)** or AHB2 **(C)**. The population of cells indicated escaping minibinder binding was sorted and deep sequenced, enabling calculation of the

“escape fraction” of each RBD mutation (the fraction of yeast expressing that mutant that were found in the escape bin). **(D)** Miniprotein escape profiles averaged across duplicate deep mutational scanning library selections. Line plots, left, show the total escape (sum of per-mutation escape) at all sites in the RBD. Sites marked in pink are shown in logoplots, right. Logoplots illustrate per-mutation escape by the height of letters reflecting individual amino acid mutations. Letters are colored according to their previously measured effects on ACE2-binding affinity *(38)*, according to the scale bar at right. **(E-F)** Correlation in escape between duplicate libraries, at the level of individual mutations **(E)** or total escape per site **(F)**.

A

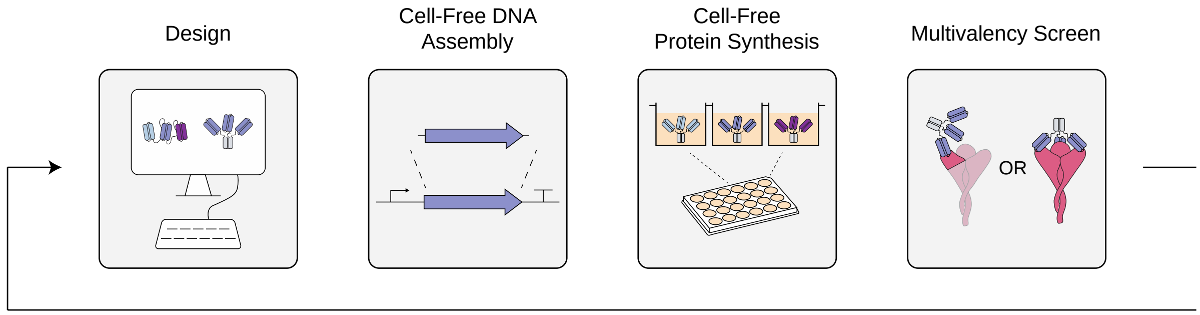

B

Cell-based protein expression

Day	Time Required (hr)	Step
1	1	Gibson assembly
1	12	Transformation
2	24	Pick colonies and culture
3	2	Miniprep cultures
4	24 to 48	Sequencing results
4	12	Transform expression strain
5	24	Start expression culture
6	1	Harvest expression culture
6	1	Cell lysis
6	1	Protein purification
6	24	Extensive protein dialysis
7	NA	Use protein

C

Cell-free protein expression

Day	Time Required (hr)	Step
1	1	Gibson assembly
1	2	LET PCR
1	4 to 20	CFPS
2	1	Protein purification
2	24	Extensive protein dialysis
3	NA	Use protein

D

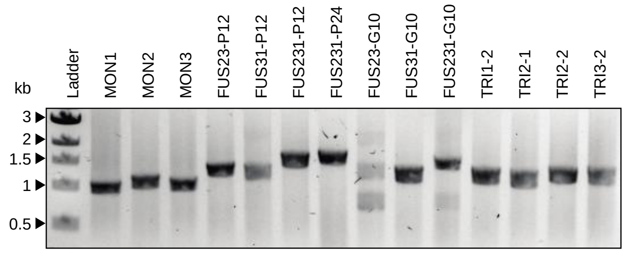

E

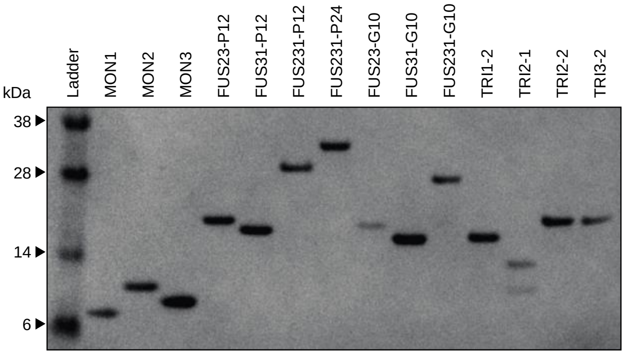

Fig. S3. Cell-free DNA assembly and protein synthesis of multivalent minibinders. Data are from a single experiment. **(A)** A cell-free workflow for the expression and evaluation of multivalent minibinders. **(B)** Step-by-step workflow for cell-based DNA assembly and protein expression. **(C)** Standard step-by-step workflow for cell-free DNA assembly and protein expression. **(D)** Agarose gel of linear DNA templates for CFPS assembled via Gibson assembly and amplified via PCR. **(E)** SDS-PAGE of purified proteins expressed via CFPS.

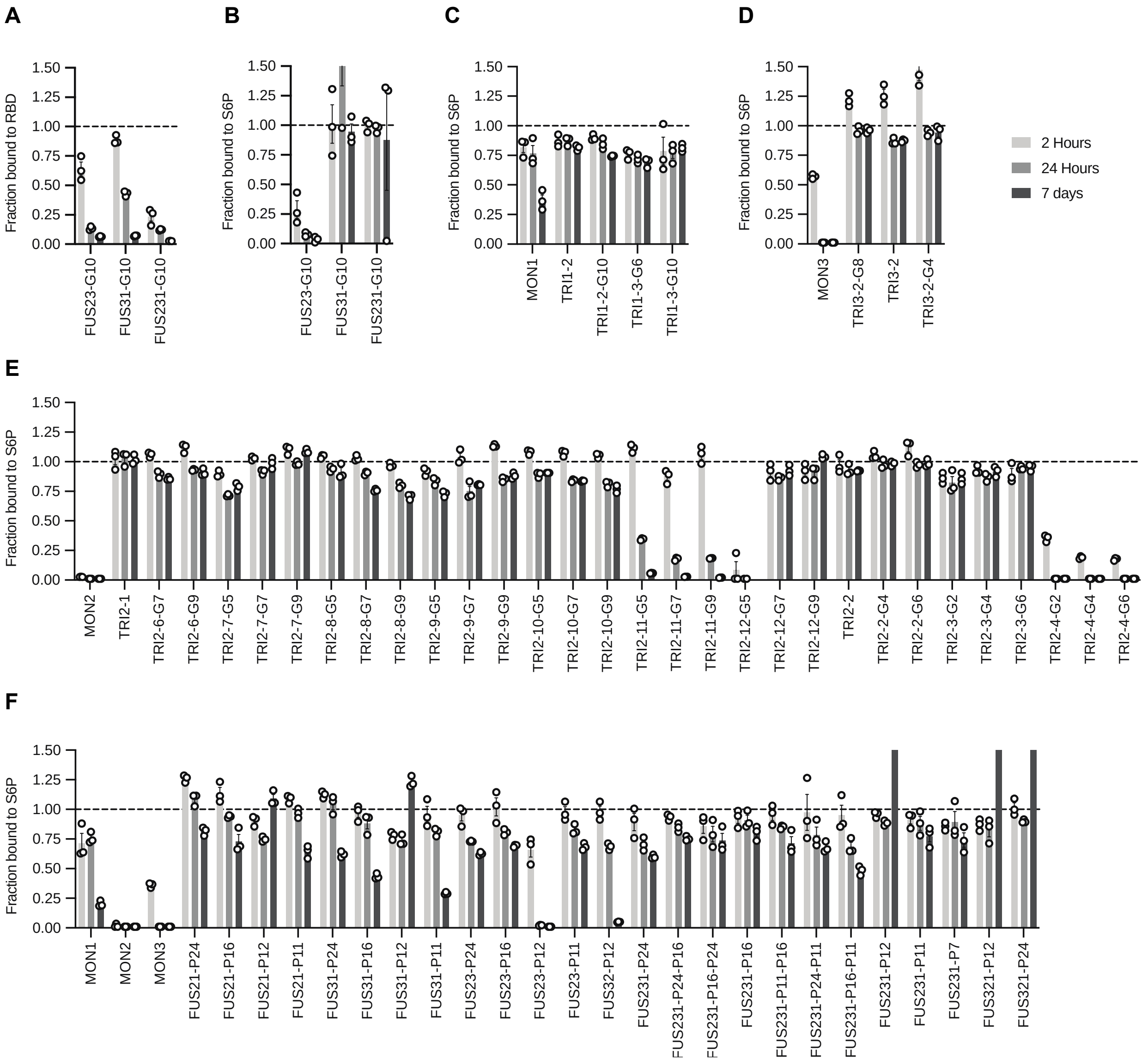

Fig. S4. Off-rate screen of multivalent minibinders. Dissociation of minibinder constructs from RBD or S6P over time was measured via competition AlphaLISA (mean ± SEM, n = 3 technical replicates from a single independent experiment). **(A)** Dissociation of FUS proteins with G10 linkers from RBD measured via competition with MON1. **(B)** Dissociation of FUS proteins with G10 linkers from S6P measured via competition with MON1. **(C)** Dissociation of TRI1 proteins from S6P measured via competition with TRI2-1. **(D)** Dissociation of TRI3 proteins from S6P measured via competition with TRI2-1. **(E)** Dissociation of TRI2 proteins from S6P measured via competition with TRI2-1. **(F)** Dissociation of FUS proteins from S6P measured via competition with FUS231-P24.

A

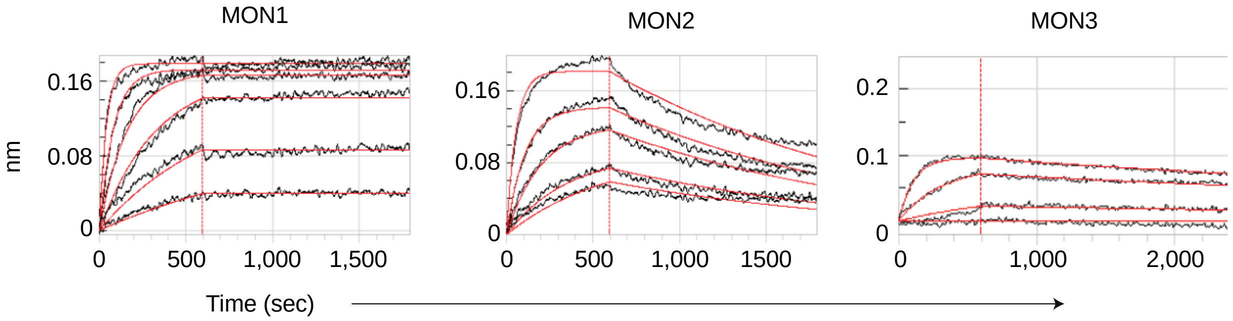

B

		MON1	MON2	MON3
RBD	k_a (x10[5] $M^{-1}s^{-1}$)	12.5	17.4	13.9
	k_d (x10[-5] s^{-1})	<4.27	61.8	15.8
	K_D (x10[-12] M)	<34.2	355	113.2

Fig. S5. Kinetics of MON1, MON2, and MON3 binding to the SARS-CoV-2 RBD. Kinetic curves of Minibinders MON1 at 2-fold dilutions from 20 – 0.625 nM, MON2 at 2-fold dilutions from 10 – 0.625 nM, and MON3 at 3-fold dilutions from 20 – 0.25 nM were analyzed against monovalent soluble RBD covalently linked to AR2G biosensors. **(A)** Sensorgrams of minibinders binding to RBD (n = 1 technical replicate from a single experiment). Red curves are fits obtained from 1:1 Langmuir binding model. Sensorgram images were copied directly from export files from ForteBio Data Analysis v11.0 software without modification. **(B)** Summary of determined kinetic and equilibrium binding parameters for each minibinder. The < symbol indicates a cut-off of less than 5% dissociation observed during the dissociation phase, indicating insufficient time to accurately quantify the dissociation rate constant. The reported rate constants and equilibrium binding constants are estimates due to the very tight binding of the minibinders studied.

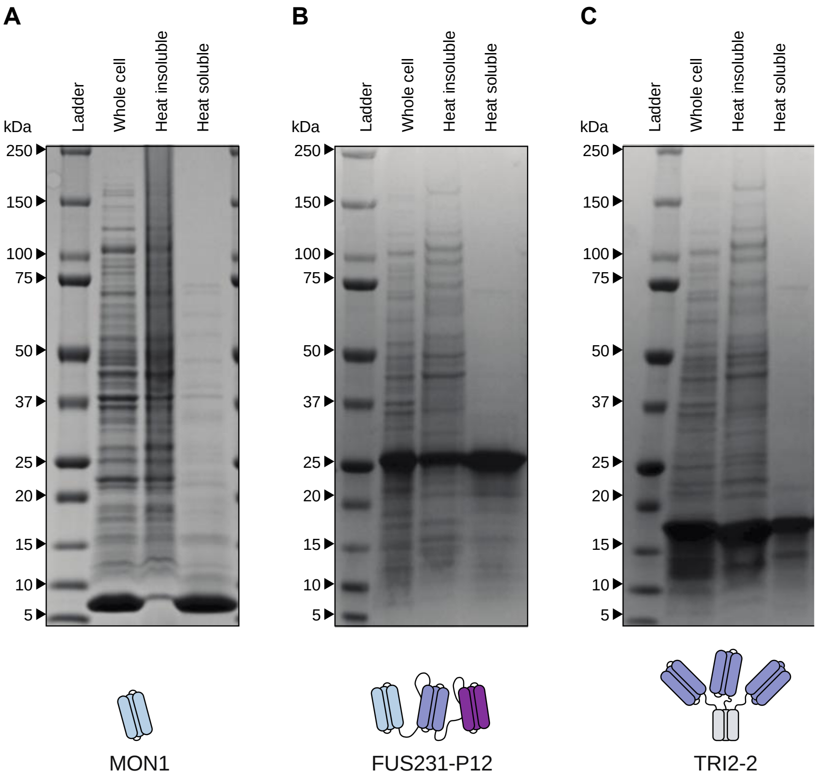

Fig. S6. Heat-based purification of mono- and multivalent minibinders. Proteins were expressed in *E. coli* using standard protein overexpression procedures. Harvested cells were heat treated at 70 °C for 5 min to lyse cells and precipitate cellular proteins. Bands observed in the Heat insoluble lane are indicative of protein precipitated during the heat treatment step. Bands observed in the Heat soluble step are indicative of proteins that did not precipitate during the heat treatment step. Data are from a single experiment. **(A)** SDS PAGE of MON1. **(B)** SDS PAGE of FUS231-P12. **(C)** SDS PAGE of TRI2-2.

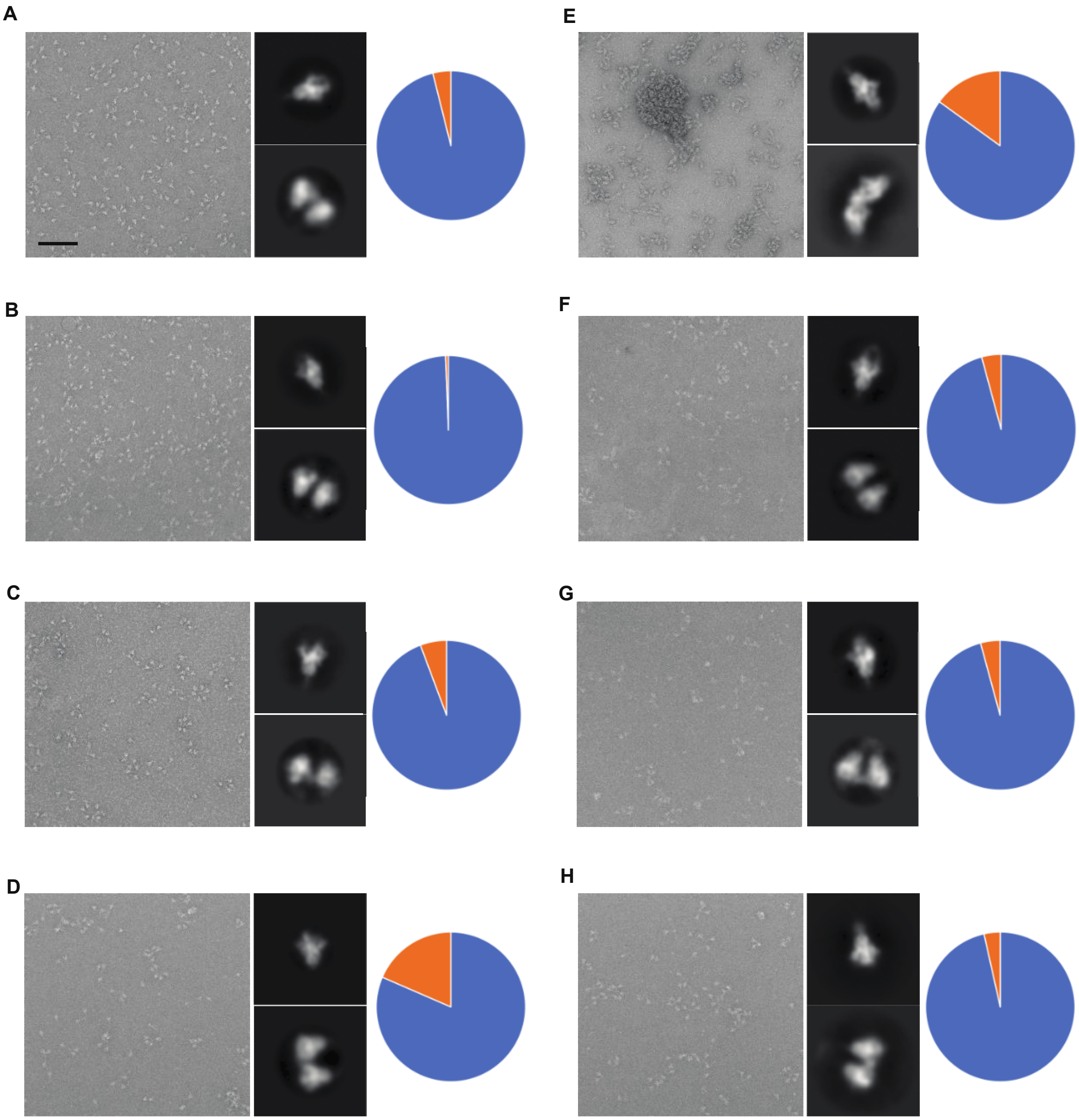

Fig. S7. Negative stain EM analysis of minibinder-mediated crosslinking and aggregation of S trimers. Representative electron micrograph and 2D class averages of SARS-CoV-2 S in complex with TRI2-2 **(A)**, FUS231-G10 **(B)**, FUS231-P24 **(C)**, FUS31-G8 **(D)**, TRI1-5-G2 **(E)**, TRI1-5-G4 **(F)**, TRI1-5-G6 **(G)** or FUS31-G10 **(H)**. Scale bar: 100 nm. After two-dimensional classification, the number of particles assigned to classes with well-defined single (blue) or two neighboring cross-linked (orange) S trimers are presented as pie charts. The fraction of total cross-linked S trimers is underestimated since higher-order cross-linked trimers did not yield well-defined 2D averages.

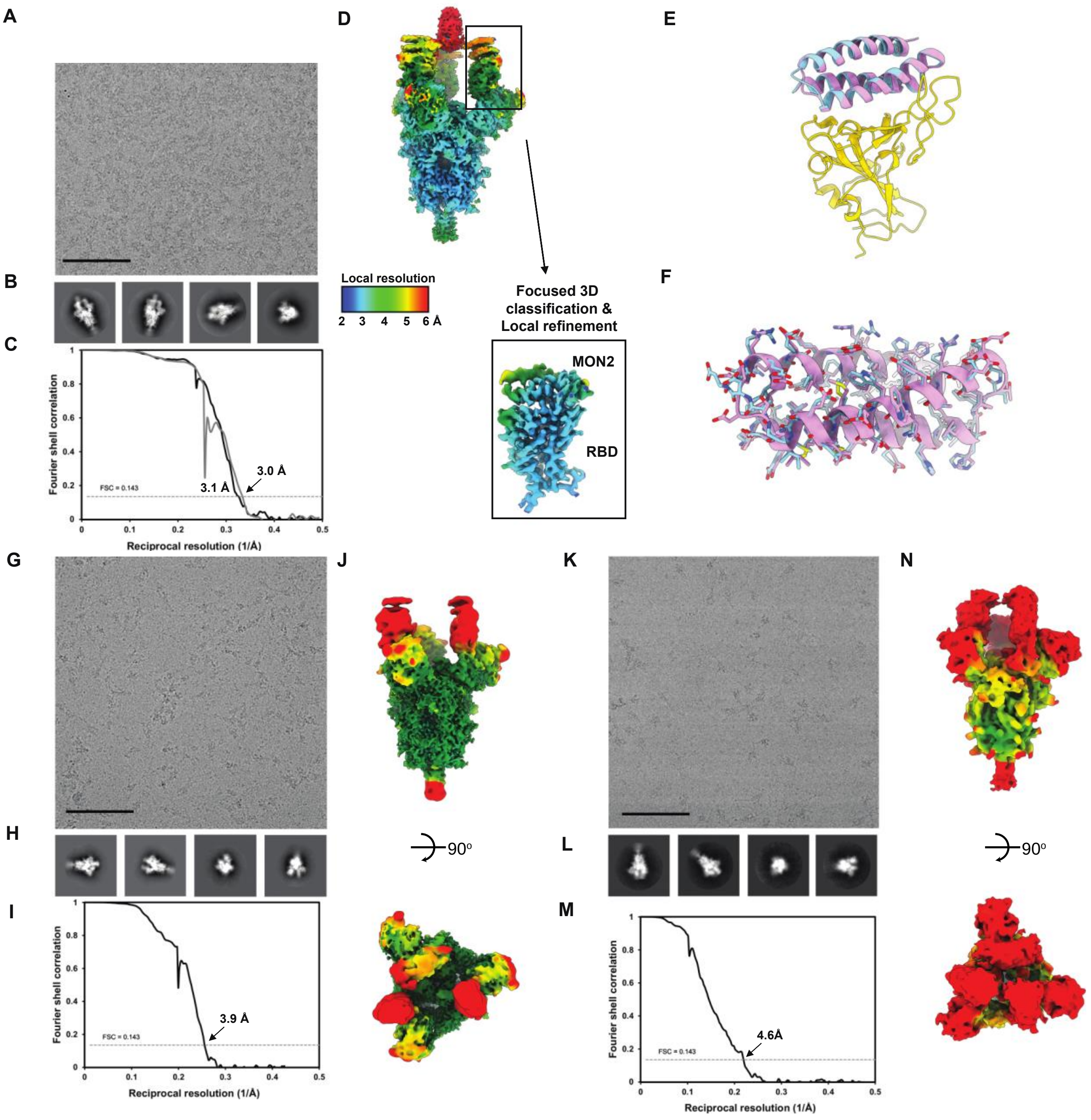

Fig. S7. Cryo-EM data collection and processing of the S6P/TRI2-2, S6P/FUS31-G10, and S6P/FUS231-P24 datasets. (A and B) Representative electron micrograph (A) and 2D class averages (B) are shown of SARS-CoV-2 S in complex with TRI2-2 embedded in vitreous ice. Scale bar: 100 nm. **(C)** Gold-standard Fourier shell correlation curves are shown for TRI2-2-bound SARS-CoV-2 S trimer (black line) and locally refined RBD/TRI2-2 region (gray line). The 0.143 cutoff is indicated by a horizontal dashed line. **(D)** Local resolution maps were calculated using cryoSPARC for the whole reconstruction as well as for the locally refined RBD/MON2 region. **(E)** A ribbon diagram of the RBD/MON2 designed model (pink) is shown superimposed with the MON2 cryoEM

structure (cyan). **(F)** The MON2 designed model (pink) was superimposed with the S6P/TRI2-2 cryoEM structure (cyan) with side chains displayed as sticks. **(G to J)** Representative electron micrograph (G), 2D class averages (H), gold-standard Fourier shell correlation curve (I) and local resolution map (J) are shown for SARS-CoV-2 S in complex with FUS231-P24 embedded in vitreous ice. **(K to N)** Representative electron micrograph (K), 2D class averages (L), gold-standard Fourier shell correlation curve (M), and local resolution map (N) are shown for SARS-CoV-2 S in complex with FUS31-G10 embedded in vitreous ice.

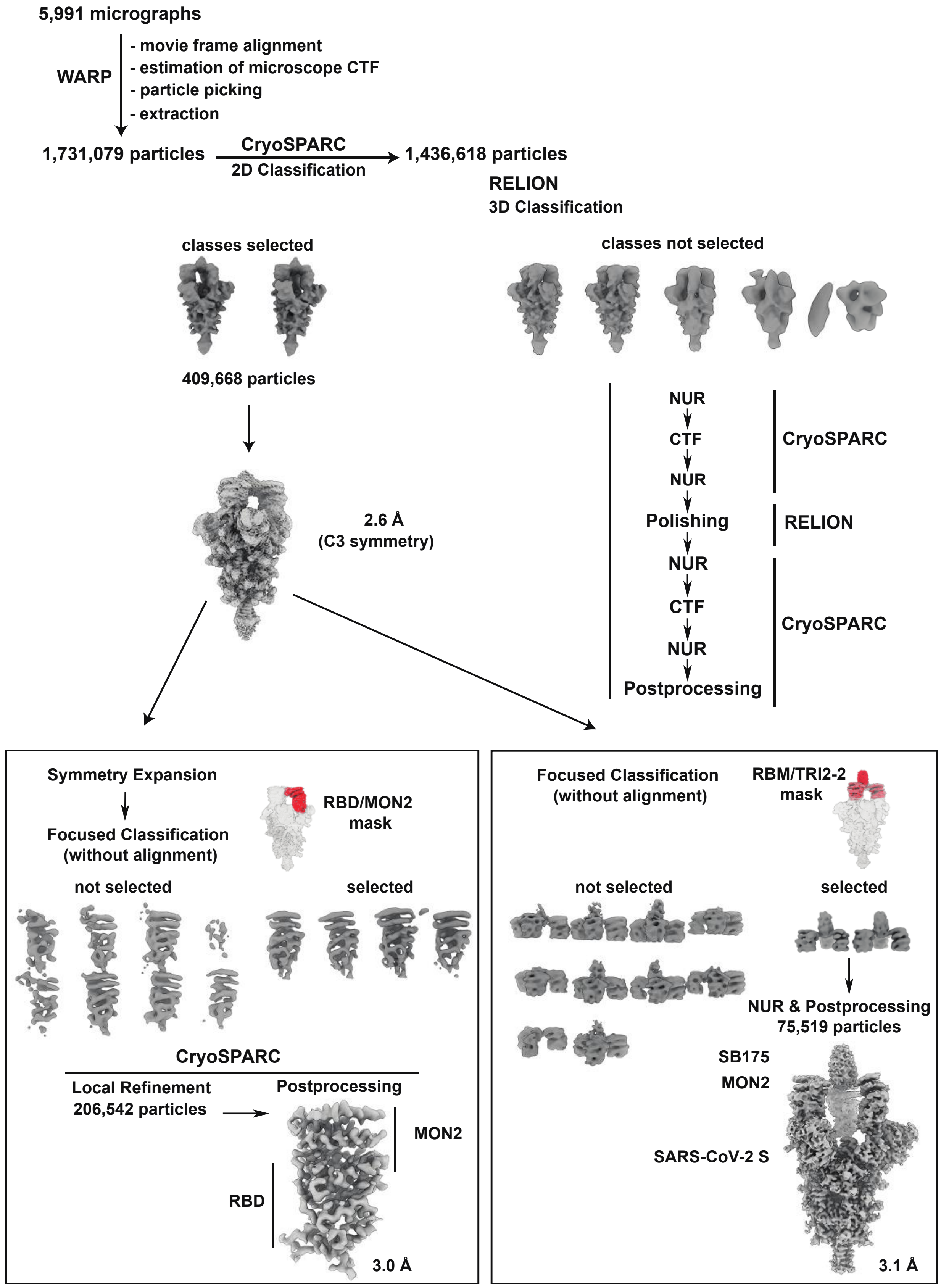

Fig. S8. Cryo-EM processing scheme of SARS-CoV-2 S/TRI2-2 complex.

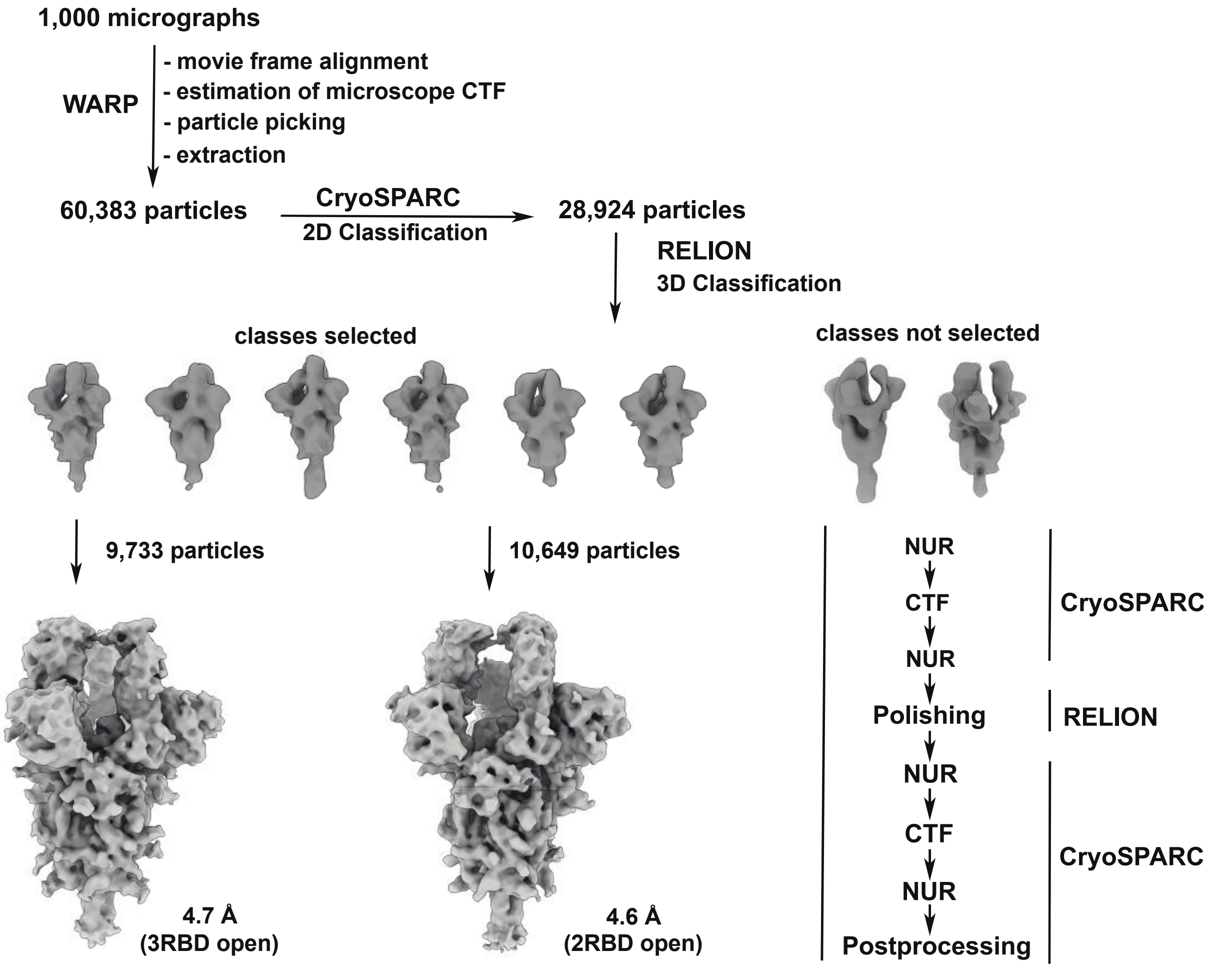

Fig. S9. Cryo-EM processing scheme of SARS-CoV-2 S/FUS31-G10 complex.

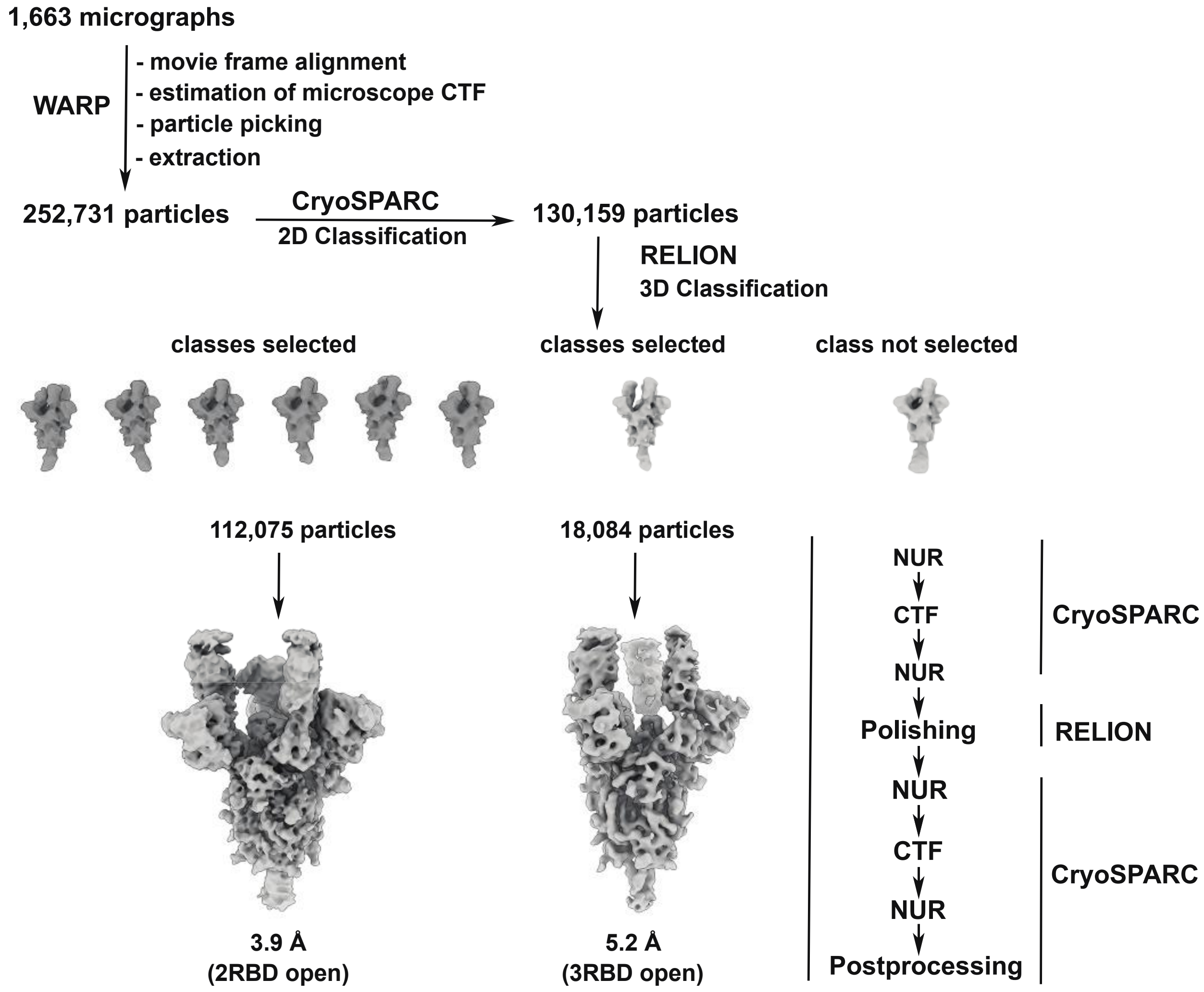

Fig. S10. Cryo-EM processing scheme of SARS-CoV-2 S/FUS231-P24 complex.

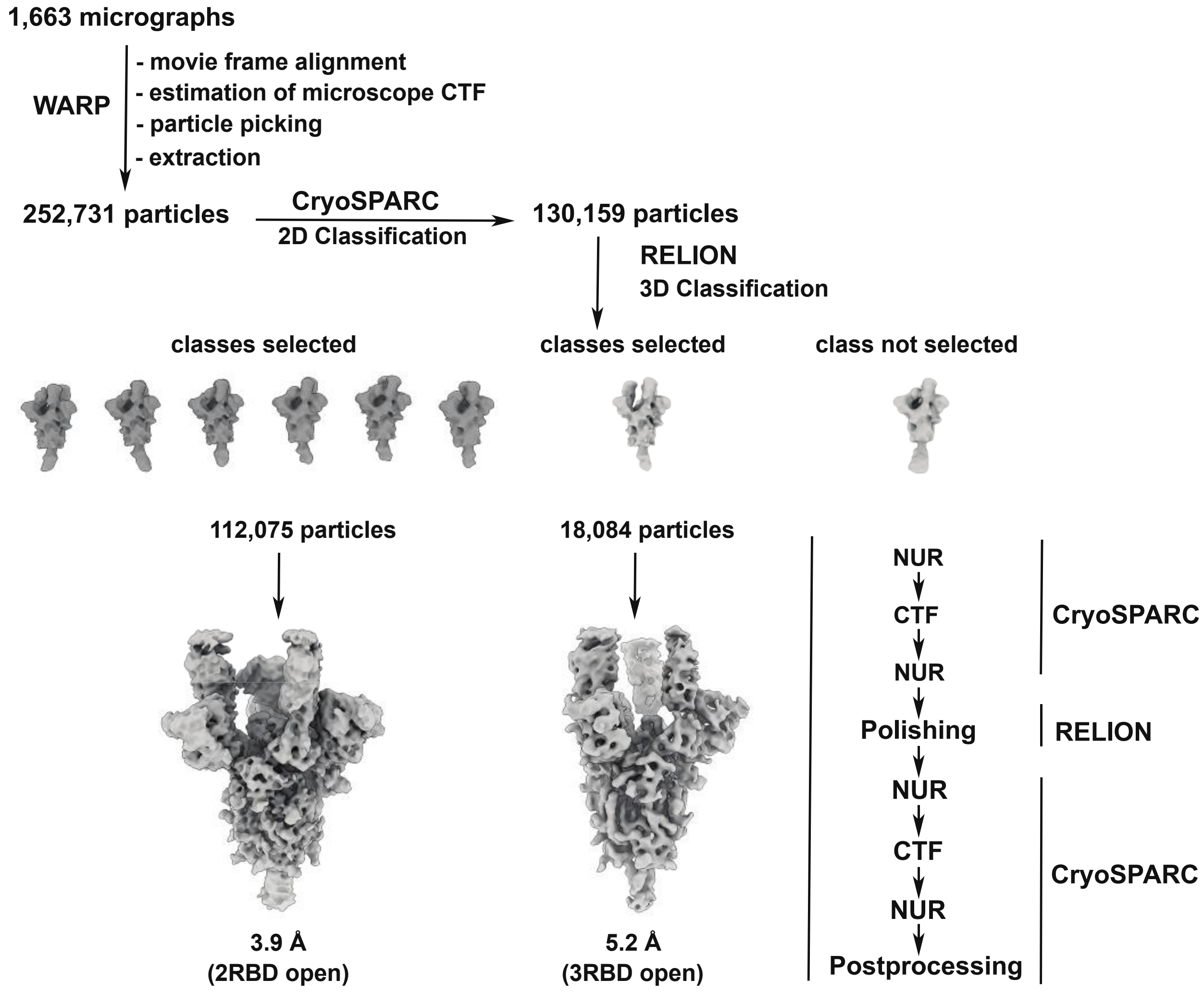

Fig. S11. Cryo-EM processing scheme of SARS-CoV-2 S/FUS231-P24 complex.

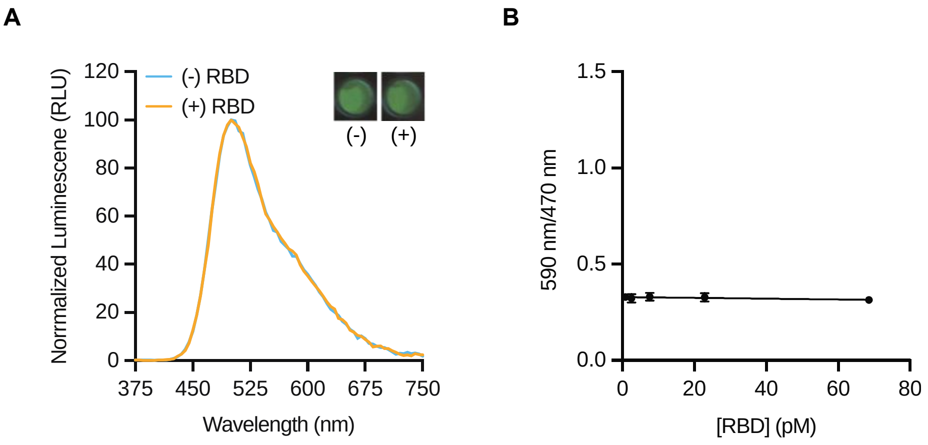

Fig. S12. FUS231-P12 BRET sensor does not detect monomeric RBD. (A) Luminescence emission spectra and image of the BRET sensor (100 pM) in the presence (yellow trace, 100 pM) and absence (blue trace) of RBD. **(B)** Titration of RBD at 100 pM sensor (Mean ± SEM, n = 3 technical replicates, error bars that aren't visible are obscured by the marker).

A

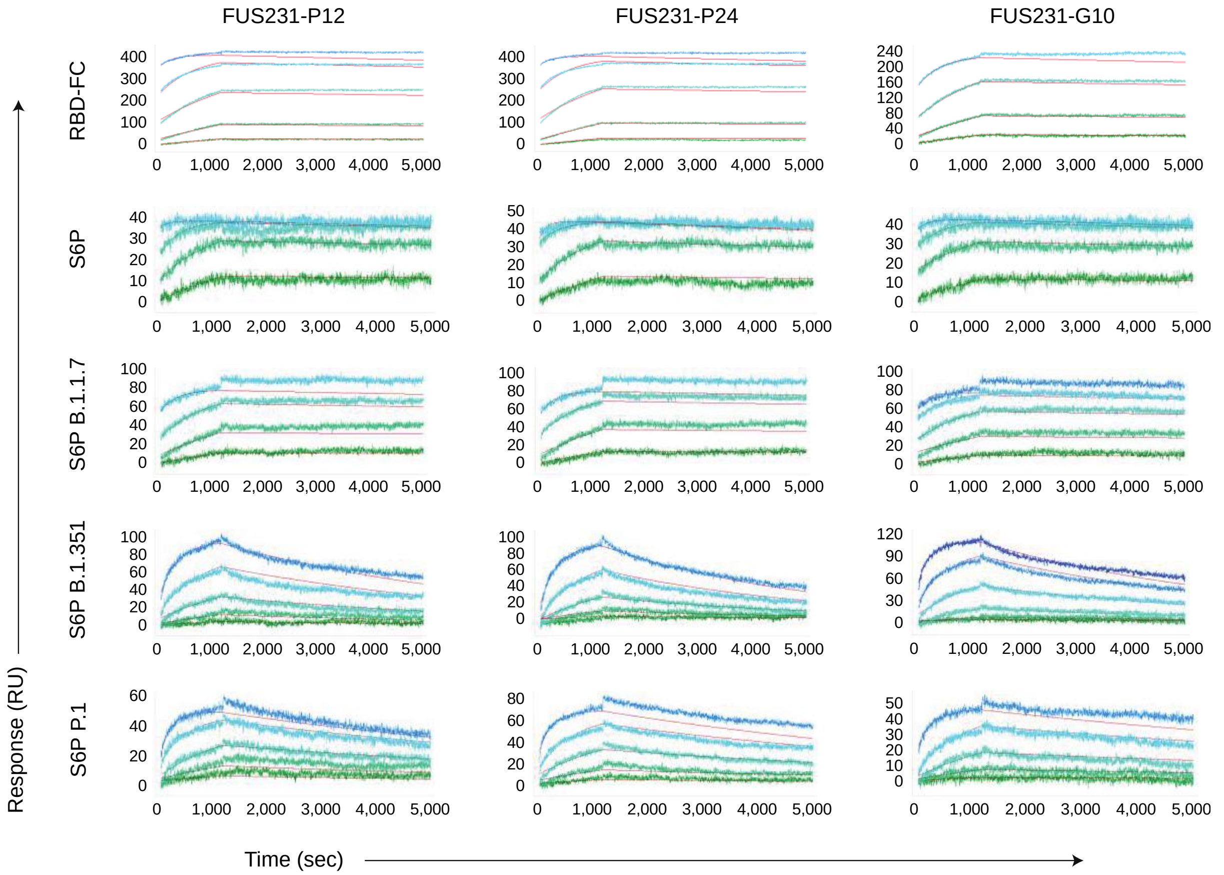

B

		FUS231-P12	FUS231-P24	FUS231-G10
RBD-FC	k_a (x10^5 $M^{-1}s^{-1}$)	6.5	7.5	9.9
	k_d (x10^{-5} s^{-1})	<1.4	<1.4	<1.4
	K_D^* (x10^{-12} M)	<21.9	<18.8	<14.4
S6P	k_a (x10^5 $M^{-1}s^{-1}$)	38.2	38.9	29.2
	k_d (x10^{-5} s^{-1})	1.7	2.7	1.9
	K_D^* (x10^{-12} M)	4.5	7.1	6.5
S6P B.1.1.7	k_a (x10^5 $M^{-1}s^{-1}$)	14.8	17.4	10.8
	k_d (x10^{-5} s^{-1})	<1.4	<1.4	<1.4
	K_D^* (x10^{-12} M)	<9.6	<82.1	<13.2
S6P B.1.351	k_a (x10^5 $M^{-1}s^{-1}$)	4.2	4.3	2.2
	k_d (x10^{-5} s^{-1})	17.6	25.8	19.9
	K_D^* (x10^{-12} M)	411.0	597.4	899.8
S6P P.1	k_a (x10^5 $M^{-1}s^{-1}$)	8.1	6.2	5.3
	k_d (x10^{-5} s^{-1})	10.9	12.2	8.6
	K_D^* (x10^{-12} M)	133.5	195.3	163.1

Fig. S13. Kinetic analysis of interactions between multivalent minibinders and SARS-CoV-2 S protein variants using surface plasmon resonance. Multivalent minibinders were injected at concentrations ranging from 20 nM to 0.08 nM in three-fold serial dilutions against S protein variants covalently coupled to the chip via amine coupling. The double-referenced data were fit globally to a simple 1:1 Langmuir binding model in Carterra's Kinetic tool. **(A)** Sensorgrams of FUS231 proteins binding to the RBD,

S6P, and S6P mutants (n = 1 technical replicate from a single independent experiment). **(B)** Summary of kinetic and equilibrium parameters of the measured interactions. *All listed K_D values are apparent K_Ds due to the potential for multivalency. The < symbol indicates a cut-off of less than 5% dissociation observed during the dissociation phase, indicating insufficient time to accurately quantify the dissociation rate constant.

A

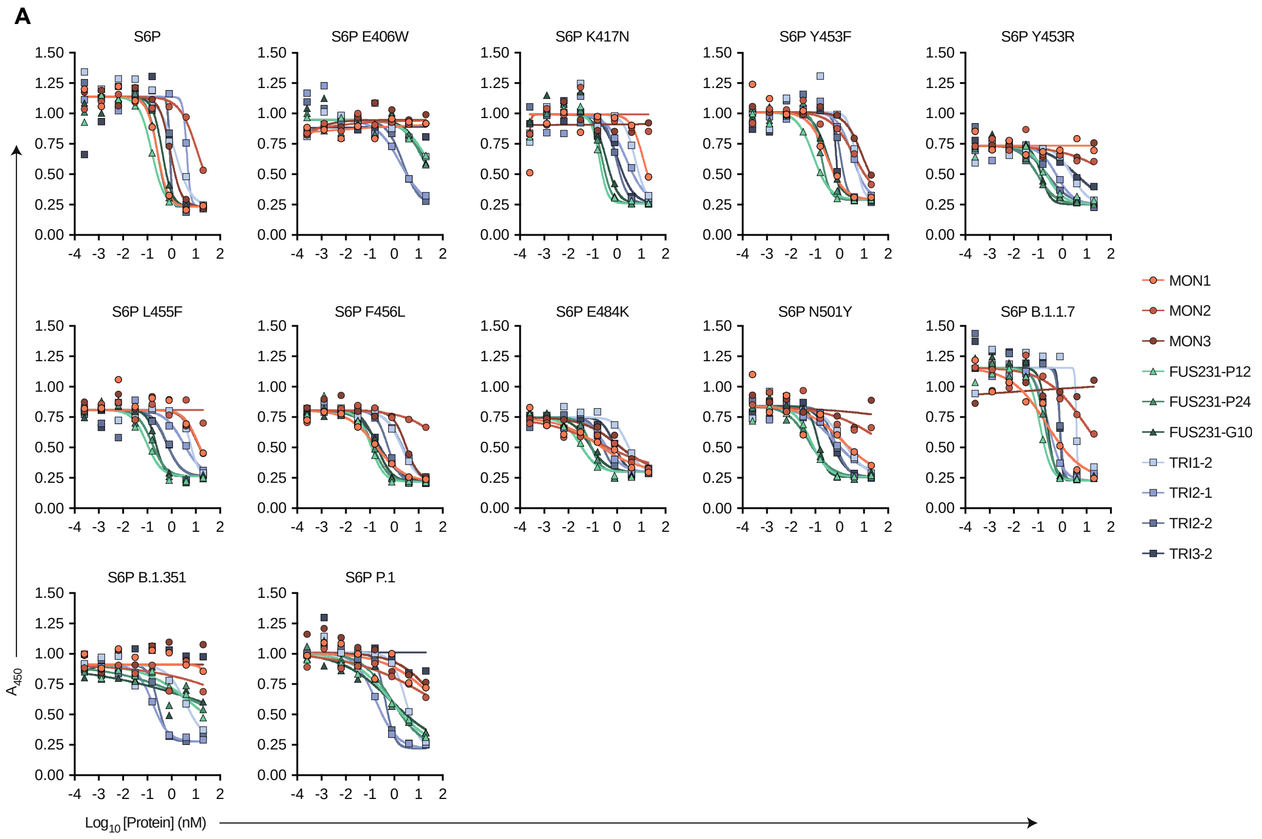

B

Protein	ACE2 Competition ELISA IC_{50} (pM) WT	E406W	K417N	Y453F	Y453R	L455F	Y456L	E484K	N510Y	B.1.1.7	B.1.351	P.1
MON1	261*	N/A	12,250	286	N/A	12,430	181*	166	1,403	224	N/A	N/A
MON2	11,700	N/A	N/A	4,011	N/A	N/A	N/A	237	N/A	8,482	N/A	N/A
MON3	895	N/A	N/A	8,743	N/A	11,870	3,079	1,028	N/A	N/A	N/A	N/A
FUS231-P12	153*	N/A	191*	83*	225*	95*	111*	31*	67*	118*	6,525	727
FUS231-P24	260*	18,300	245*	175*	96*	156*	134*	81	55*	187	12,820	803
FUS231-G10	415*	18,930	361*	319	138*	223*	194*	74	132	240	N/A	733
TRI1-2	1,388	N/A	5,628	4,479	2,458	5,897	1,939	2,721	1,404	3,830	3,832	2,997
TRI2-1	4,247	1,720*	2,412	3,362	573	3,052	2,182	329	631	267	165	175
TRI2-2	870	2,242*	934	1,090	249*	648*	543*	244	625	792	270	429
TRI3-2	844	N/A	1,086	722	3,936	627*	246*	772	464	786	N/A	N/A

C

S6P Variant	[S6P] (pM)
WT	1,200
E406W	6,900
K417N	1,700
Y453F	500
Y453R	600
L455F	1,300
F456L	1,100
E484K	100
N501Y	200
B.1.1.7	300
B.1.351	200
P.1	200

D

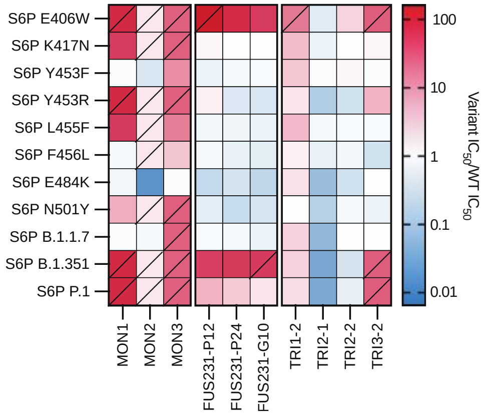

Fig. S14. Competition of ACE2 and mini binder constructs for S6P. (A) Competition ELISA curves (mean, n = 2 technical replicates representative of two independent experiments). **(B)** Summary of minibinder construct competition IC_{50} values. An * denotes

an IC_{50} value less than 2-fold lower than the limiting concentration of S6P variant present in the well. N/A indicates an IC_{50} value higher than the tested concentration range and greater than 20,000 pM. **(C)** Summary of EC_{50} values of S6P variants binding to ACE2 used as the concentration for minibinder construct competition. **(D)** Ratio of mutant to wild type (WT) IC_{50} values of minibinder constructs. The ratio in cells containing a slash was determined using the highest tested minibinder construct concentration which is indicative of a measurement at the upper limit of detection.

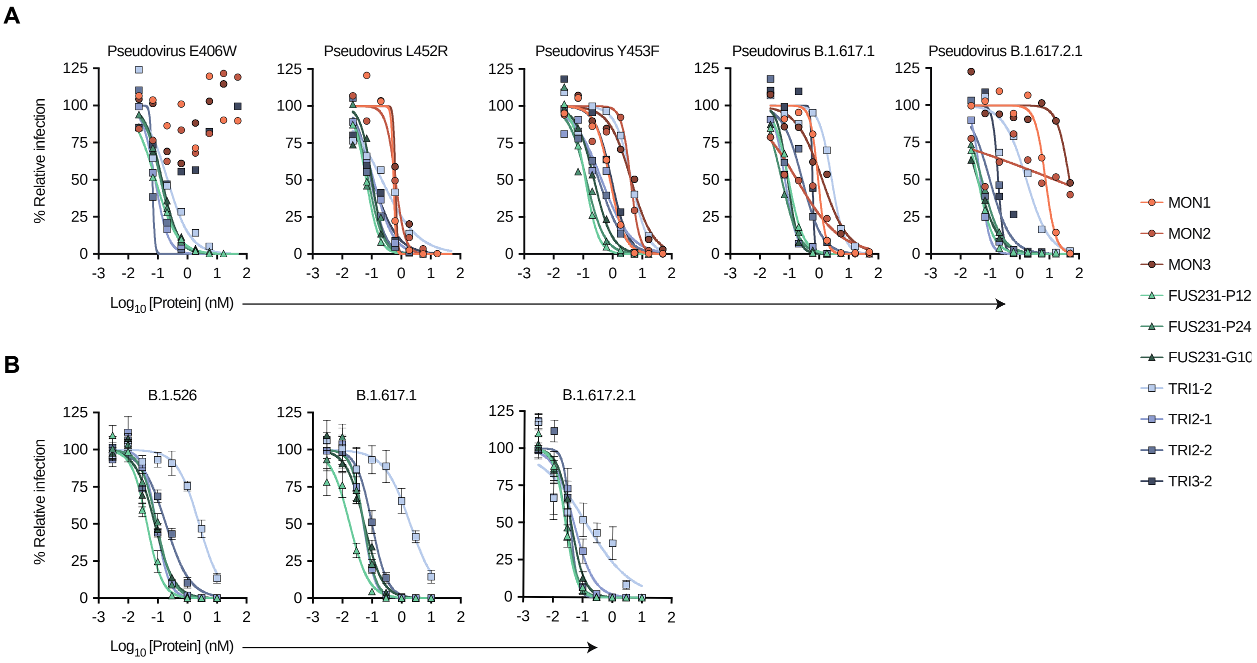

C

Protein	Pseudovirus Neutralization IC_{50} (pM) E406W	L452R	Y453F	B.1.617.1	B.1.617.2.1
MON1	N/A	570	817	941	7,421
MON2	N/A	639	4,102	180	47,860
MON3	N/A	637	4,260	1,296	15,050
FUS231-P12	84	65	120	94	39
FUS231-P24	124	77	130	55	53
FUS231-G10	138	103	237	88	38
TRI1-2	214	223	4,406	2,714	47
TRI2-1	81	117	430	71	98
TRI2-2	67	94	343	273	1,680
TRI3-2	N/A	109	928	607	188

D

Protein	Authentic SARS-CoV-2 Variant Neutralization IC_{50} (pM) B.1.526	B.1.617.1	B.1.617.2.1
FUS231-P12	43	18	26
FUS231-P24	92	53	27
FUS231-G10	80	55	40
TRI1-2	2,504	1,971	127
TRI2-1	90	52	49
TRI2-2	188	101	38

Fig. S15. Neutralization of additional SARS-CoV-2 variants. (A) Neutralization of SARS-CoV-2 pseudovirus variants by minibinder constructs (mean, n = 2 technical replicates representative of two independent experiments). **(B)** Neutralization of SARS-CoV-2 variants by minibinder constructs (mean ± SEM, n = 4 technical replicates from two independent experiments). **(C)** Table summarizing neutralization potencies of multivalent minibinder constructs against SARS-CoV-2 pseudovirus variants. N/A indicates an IC_{50} value above the tested concentration range and an IC_{50} greater than 50,000 pM. **(D)** Table summarizing neutralization potencies of multivalent minibinder constructs against SARS-CoV-2 variants.

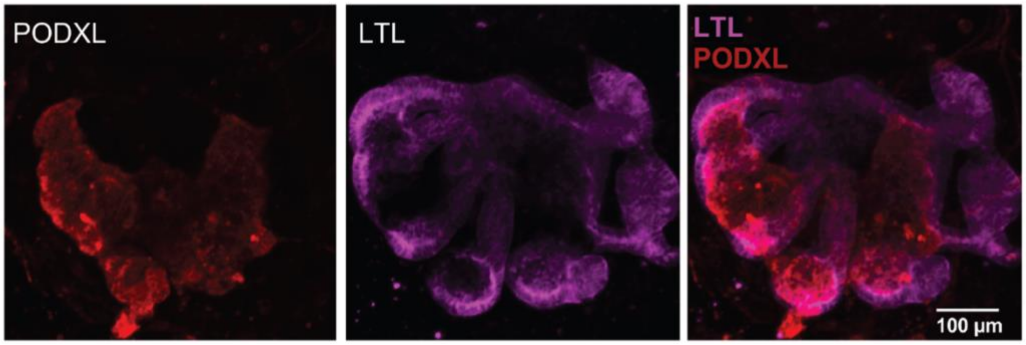

Fig. S16. Representative confocal images of human kidney organoids. Organoids were derived from H9 human embryonic stem cells (LTL, *Lotus tetragonolobus* lectin, proximal tubule marker in magenta; PODXL, podocalyxin, podocyte marker in red).

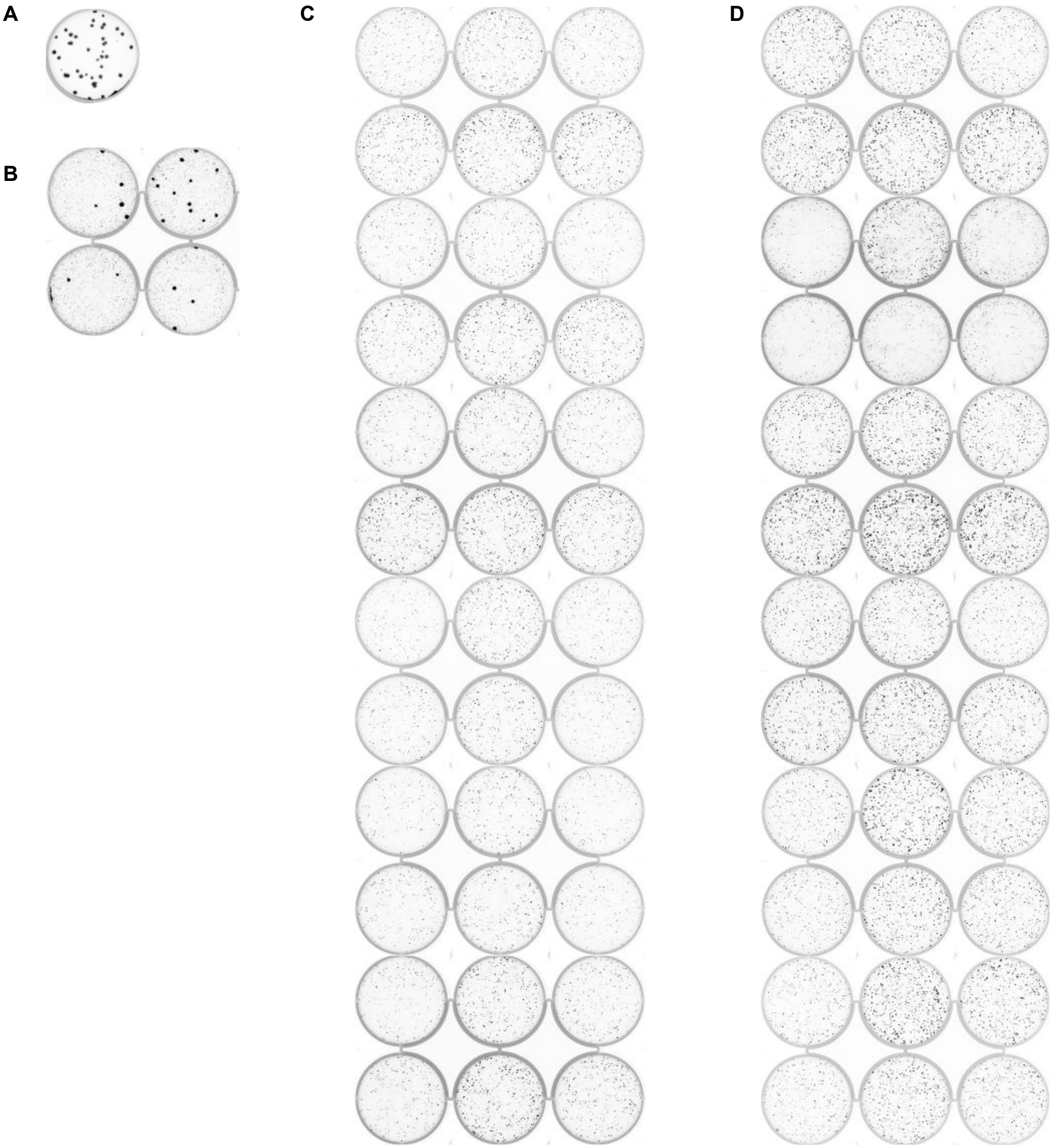

Fig. S17. Replicate plates for VSV-SARS-CoV-2 escape studies. Plaque assays were performed to isolate VSV-SARS-CoV-2 chimera virus escape mutants against a control neutralizing antibody (2B04) and the FUS231-P12 and TRI2-2 multivalent minibinders. Large plaques are indicative of escape. FUS231-P12 and TRI2-2 replicates were performed in three separate experiments consisting of two plates each. **(A)** No inhibitor in overlay. **(B)** 2B04 neutralizing mAb in overlay. **(C)** FUS231-P12 in overlay. **(D)** TRI2-2 in overlay.

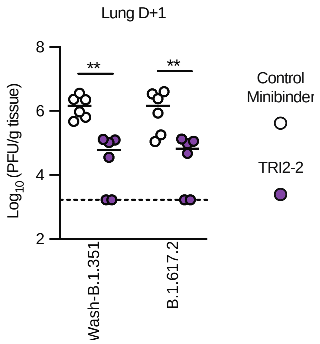

Fig. S18. Measurement of SARS-CoV-2 viral titers in the lung of K18-hACE2-transgenic mice post intranasal delivery of TRI2-2. At 6 dpi (B.1.351) or 7 dpi (B.1.617.2), animals (n = 6 from two independent experiments) were sacrificed and analyzed for SARS-CoV-2 viral titers in the lung by plaque assays (line at median, two-tailed Mann-Whitney test: ** $P < 0.01$).

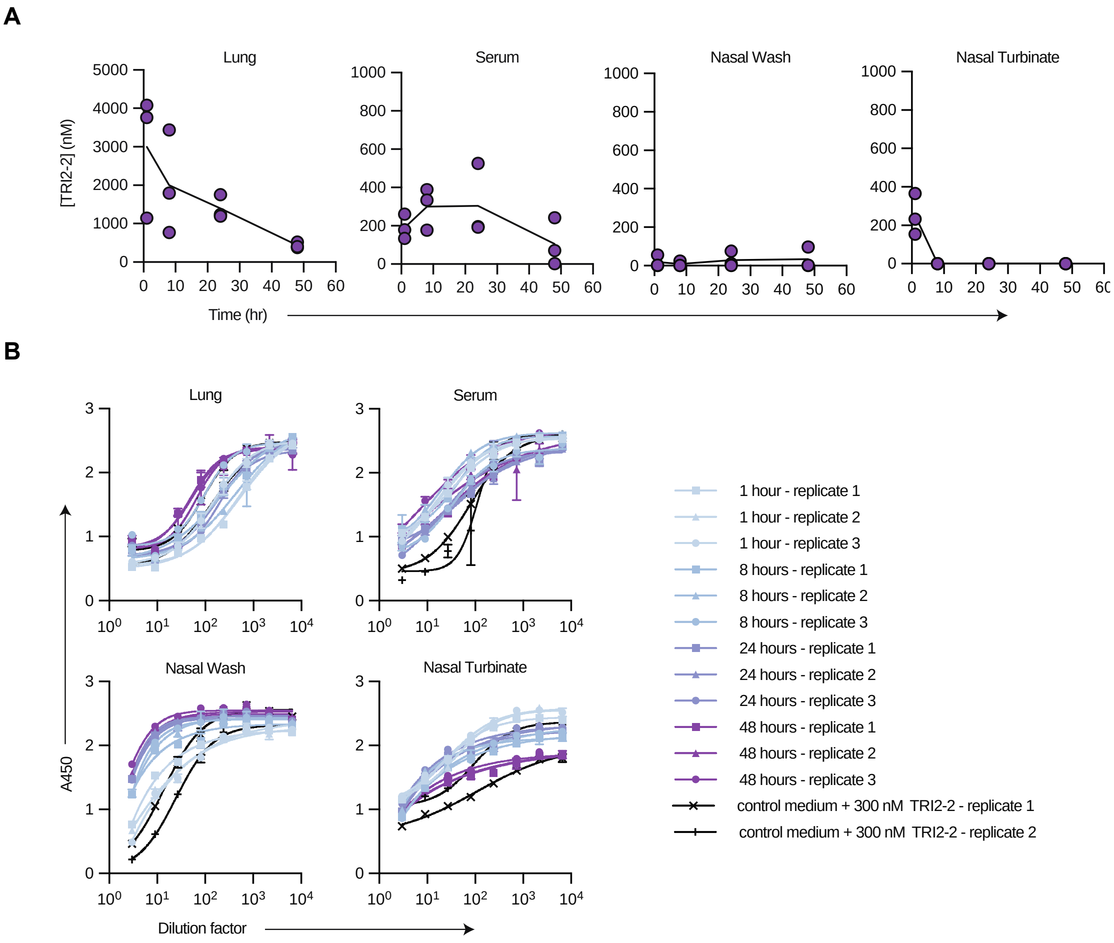

Fig. S19. Pharmacokinetics of TRI2-2 delivered intranasally in C57BL/6J mice. (A) Serum and tissues were collected at 1-, 8-, 24-, and 48-hours post-treatment with 200 µg of TRI2-2 and quantitative competition ELISAs were used to determine concentrations of TRI2-2 present in the samples (n = 3 from a single experiment). The drawn line connects the mean values at each time point. **(B)** Raw data from competition ELISAs. Each curve represents a biological replicate with each data point consisting of n = 2 technical replicates.

Table S1. List of abbreviations used to describe multivalent minibinders in this article.

Abbreviation	Protein (N to C)
MON1	LCB1v2.2
MON2	AHB2
MON3	LCB3v2.2
FUS23-P12	AHB2v2-P12-LCB3v2.2
FUS31-P12	LCB3v2.2-P12-LCB1v2.2
FUS231-P12	AHB2v2-P12-LCB3v2.2-P12-LCB1v2.2
FUS231-P24	AHB2v2-P24-LCB3v2.2-P24-LCB1v2.2
FUS23-G10	AHB2v2-G10-LCB3v2.2
FUS31-G10	LCB3v2.2-G10-LCB1v2.2
FUS231-G10	AHB2v2-G10-LCB3v2.2-G10-LCB1v2.2
TRI1-2	SB175-6G-LCB1v2.2
TRI2-1	AHB2-4G-1rfo
TRI2-2	AHB2-2G-SB175
TRI3-2	LCB3v2.2-6G-SB175
TRI2-6-G7	AHB2-7G-1na0_int2-R3
TRI2-6-G9	AHB2-9G-1na0_int2-R3
TRI2-7-G5	AHB2-5G-1na0_int2
TRI2-7-G7	AHB2-7G-1na0_int2
TRI2-7-G9	AHB2-9G-1na0_int2
TRI2-8-G5	AHB2-5G-6msr

Abbreviation	Protein (N to C)
TRI2-3-G4	AHB2-4G-SB175.1
TRI2-3-G6	AHB2-6G-SB175.1
TRI2-4-G2	AHB2-2G-SB175.2
TRI2-4-G4	AHB2-4G-SB175.2
TRI2-4-G6	AHB2-6G-SB175.2
TRI1-5-G2	36729-2G-LCB1v2.2
TRI1-5-G4	36729-4G-LCB1v2.2
TRI1-5-G6	36729-6G-LCB1v2.2
TRI1-2-G10	SB175-10G-LCB1v2.2
TRI1-3-G6	SB175.1-6G-LCB1v2.2
TRI1-3-G10	SB175.1-10G-LCB1v2.2
TRI3-2-G8	LCB3v2.2-8G-SB175
TRI3-2-G4	LCB3v2.2-4G-SB175
FUS21-P24	AHB2-P24-LCB1v2.1
FUS21-P16	AHB2-P16-LCB1v2.1
FUS21-P12	AHB2-P12-LCB1v2.2
FUS21-P11	AHB2-P11-LCB1v2.1
FUS31-P24	LCB3v2.2-P24-LCB1v2.1
FUS31-P16	LCB3v2.2-P16-LCB1v2.1
FUS31-P11	LCB3v2.2-P11-LCB1v2.1

TRI2-8-G7	AHB2-7G-6msr
TRI2-8-G9	AHB2-9G-6msr
TRI2-9-G5	AHB2-5G-1gcm
TRI2-9-G7	AHB2-7G-1gcm
TRI2-9-G9	AHB2-9G-1gcm
TRI2-10-G5	AHB2-5G-pRO-2-noHis
TRI2-10-G7	AHB2-7G-pRO-2-noHis
TRI2-10-G9	AHB2-9G-pRO-2-noHis
TRI2-11-G5	AHB2-5G-1na0_3
TRI2-11-G7	AHB2-7G-1na0_3
TRI2-11-G9	AHB2-9G-1na0_3
TRI2-12-G5	AHB2-5G-4pn9
TRI2-12-G7	AHB2-7G-4pn9
TRI2-12-G9	AHB2-9G-4pn9
TRI2-2-G4	AHB2-4G-SB175
TRI2-2-G6	AHB2-6G-SB175
TRI2-3-G2	AHB2-2G-SB175.1

FUS23-P24	AHB2-P24-LCB3v2.3
FUS23-P16	AHB2-P16-LCB3v2.3
FUS23-P12	AHB2-P12-LCB3v2.2
FUS23-P11	AHB2-P11-LCB3v2.3
FUS32-P12	LCB3v2.2-P12-AHB2v2
FUS231-P24-P16	AHB2v2-P24-LCB3v2.2-P16-LCB1v2.2
FUS231-P16-P24	AHB2v2-P16-LCB3v2.2-P24-LCB1v2.2
FUS231-P16	AHB2v2-P16-LCB3v2.2-P16-LCB1v2.2
FUS231-P11-P16	AHB2v2-P11-LCB3v2.2-P16-LCB1v2.2
FUS231-P24-P11	AHB2v2-P24-LCB3v2.2-P11-LCB1v2.2
FUS231-P16-P11	AHB2v2-P16-LCB3v2.2-P11-LCB1v2.2
FUS231-P11	AHB2v2-P11-LCB3v2.2-P11-LCB1v2.2
FUS231-P7	AHB2v2-P7-LCB3v2.2-P7-LCB1v2.2
FUS321-P12	LCB3v2.2-P12-AHB2v2-P12-LCB1v2.2
FUS321-P24	LCB3v2.2-P24-AHB2v2-P24-LCB1v2.2
FUS31-G8	LCB3v2.2-G8-LCB1v2.2

Table S2. Oligomerization domains tested in this work.

#	Name	Reference	Notes	Protein Sequence
1	1rfo	*(45)*		GYIPEAPRDGQAYVRKDGEWVLLSTFL
2	SB175	This work	Modified from SB13 (2L6HC3_13) *(112)*	SEALEELEKALRELKKSTDELERSTEELEKNPSEDALVENNRLIVENNKIIVEVLRIIAKVLK
3	SB175.1	This work	Medium truncation of SB175 helices	SPELEKALRELKKSTDELERSTEELEKNGSPEALVENNRLIVENNKIIVEVLRIIAK
4	SB175.2	This work	Large truncation of SB175 helices	SEKALRELKKSTDELERSTEELEKNGSPEALVENNRLIVENNKIIVEVLR
5	36729.2	This work	Modified from 1na0_int2	EEAELAYLLGELAYKLGEYRIAIRAYRIALKRDPNNAEAWYNLGNAYYKQGDYDEAIEYYQKALELDPNNAEAWYNLGNAYYKQGDYDEAIEYYQKALELDPNNAEAWYNLGNAYYKQGDYDEAIEYYQKALEL
6	1na0_int2-R3	This work	Modified from 1na0_int2	EEAELAYLLGELAYKLGEYRIAIRAYRIALKRDPNNAEAWYNLGNAYYKQGDYDEAIEYYQKALELDPNNAEAKQNLGNAKQKQG
7	1na0_int2	This work	Modified from *(113)*	EEAELAYLLGELAYKLGEYRIAIRAYRIALKRDPNNAEAWYNLGNAYYKQGDYDEAIEYYQKALELDPNNAEAWYNLGNAYYKQGDYDEAIEYYQKALELDPNNAEAKQNLGNAKQKQG
8	6msr	*(114)*		GSEYEIRKALEELKASTAELKRATASLRASTEELKKNPSEDALVENNRLIVEHNAIIVENNRIIAAVLELIVRAIK
9	1gcm	*(115)*		RMKQIEDKIEEILSKIYHIENEIARIKKLIGER
10	pRO-2-noHis	*(114)*		GSEYEIRKALEELKASTAELKRSTASLRASTEELKKNPSEDALVENNRLIVENNAIIVENNRIIAAVLELIVRAIK
11	1na0_3	This work		NLAEKMYKAGNAMYRKGQYTIAIIAYTLALLKDPNNAEAWYNLGNAAYKKGEYDEAIEAYQKALELDPNNAEAWYNLGNAYYKQGDYDEAIEYYQKALELDPNNAEAKQNLGNAKQKQG
12	4pn9	*(116)*	Hexamer not trimer	GEIAKSLKEIAKSLKEIAWSLKEIAKSLKG

Table S3. CryoEM data collection and refinement statistics.

	SARS-CoV-2 S/TRI2-2 PDB 7UHC EMD-26512	SARS-CoV-2 S/TRI2-2 (local refinement) PDB 7UHB EMD-26511	SARS-CoV-2 S/FUS31-G10 (2RBD-open) EMD-26509	SARS-CoV-2 S/FUS31-G10 (3RBD-open) EMD-26510	SARS-CoV-2 S/FUS231-P24 (2RBD-open) EMD-26507	SARS-CoV-2 S/FUS231-P24 (3RBD-open) EMD-26508
**Data collection and processing**						
Magnification	105,000	105,000	130,000	130,000	36,000	36,000
Voltage (kV)	300	300	300	300	200	200
Electron exposure ($e^-/Å^2$)	60	60	70	70	60	60
Defocus range (μm)	-0.5 – -2.5	-0.5 – -2.5	-0.5 – -2.5	-0.5 – -2.5	-0.5 – -2.5	-0.5 – -2.5
Pixel size (Å)	0.4215	0.4215	0.525	0.525	1.16	1.16
Symmetry imposed	C3	C1	C1	C1	C1	C1
Final particle images (no.)	75,519	206,541	9,733	10,649	112,075	18,084
Map resolution (Å)	3.12.8	3.02.9	4.57	4.65	3.9	5.2
FSC threshold	0.143	0.143	0.143	0.143	0.143	0.143
Map sharpening *B*factor ($Å^2$)	-63	-31	-63	-71	-99	-167
**Validation**						
MolProbity score	1.05	1.03				
Clashscore	1.19	1.62				
Poor rotamers (%)	0	0				
Ramachandran plot						
Favored (%)	96.62	97.37				
Allowed (%)	2.99	2.25				
Disallowed (%)	0.39	0.38				

Table S4. Comparison of multivalent minibinder and FDA authorized neutralizing antibody potencies. For mAb data, pseudovirus neutralization IC_{50} values (columns 3, 4, and 5) were taken from *(15)* and authentic virus neutralization IC_{50} values (columns 6, 7, and 8) were taken from *(16)*. *Value for WA1/2020 strain as opposed to WA1/2020 D614G.

Neutralizing Protein	Commercial Name	Wu-Hu-1 Pseudovirus IC50 (ng/mL)	B.1.1.529 Pseudovirus IC50 (ng/mL)	Fold-Change Pseudovirus IC50 (B.1.1.529/Wu-Hu-1)	WA1/2020 D614G Authentic Virus IC50 (ng/mL)	B.1.1.529 Authentic Virus IC50 (ng/mL)	Fold-Change Authentic Virus IC50 (B.1.1.529/WA1/2020 D614G)
S309 (VIR-7831)	Sotrovimab	90.6	260	2.9	202	373	1.8
REGN10933	Casirivimab	8.9	>10,000	>1123.6	11	>10,000	>909.1
REGN10987	Imdevimab	25.1	>10,000	>398.4	31	>10,000	>322.6
LY-CoV555	Etesevimab	21.3	>10,000	>469.5	10	>10,000	>1000
LY-CoV016 (CB6)	Bamlanivimab	59.2	>10,000	>168.9	72	>10,000	>138.9
COV2-2196 (AZD8895)	Tixagevimab	8.1	2,772	342.2	6	913	152.2
COV2-2130 (AZD1061)	Cilgavimab	4.3	>10,000	>2325.6	32	381	11.9
TRI2-1	n/a	8.2	n/a	n/a	0.4*	0.7	1.8
TRI2-2	n/a	8.4	n/a	n/a	1.2*	6.2	5.2

Table S5. Estimates of the diversity of mutants in the VSV-SARS-CoV-2 chimera virus pool used in the multivalent minibinder escape selections. Estimates were calculated using the Pedel-AA tool *(109)* for analyzing diversity in randomly mutated protein libraries. Upper and lower estimates for the VSV RNA polymerase L error rate *(61)* were used to calculate the range of plausible library compositions.

		Polymerase Error Rate (error/nucleotide): 10^{-3}		Polymerase Error Rate (error/nucleotide): 10^{-4}	
# Amino Acid Mutations	# Possible Mutants	# Expected Distinct Mutants	% Coverage	# Expected Distinct Mutants	% Coverage
1	2,071	1,700	82.09	703.9	33.99
2	$2.13x10^6$	$2.04x10^5$	9.62	9,420	0.44

Data File S1 (.xlsx). List of DNA and protein sequences for multivalent minibinders used in this article. DNA sequences are the open reading frame coding for the expressed protein. Protein sequences are annotated as follows. Minibinder and homotrimer sequences are denoted by square brackets []. Secondary sequences (e.g., expression tag, purification tag, etc.) are annotated by parenthesis (). Non-minibinder or non-homotrimer sequences are annotated by curly brackets {} (captures linkers and other secondary sequences).

Data File S2 (.xlsx). Individual data for all main text figures. Data are organized by figure panel, with one figure panel per worksheet tab.

Data File S3 (.xlsx). Individual data for all supplementary figures. Data are organized by figure panel, with one figure panel per worksheet tab.

References:

1. A. C. Walls, Y. J. Park, M. A. Tortorici, A. Wall, A. T. McGuire, D. Veesler, Structure, Function, and Antigenicity of the SARS-CoV-2 Spike Glycoprotein. *Cell* **181**, 281-292.e6 (2020).

2. M. Dougan, A. Nirula, M. Azizad, B. Mocherla, R. L. Gottlieb, P. Chen, C. Hebert, R. Perry, J. Boscia, B. Heller, J. Morris, C. Crystal, A. Igbinadolor, G. Huhn, J. Cardona, I. Shawa, P. Kumar, A. C. Adams, J. Van Naarden, K. L. Custer, M. Durante, G. Oakley, A. E. Schade, T. R. Holzer, P. J. Ebert, R. E. Higgs, N. L. Kallewaard, J. Sabo, D. R. Patel, M. C. Dabora, P. Klekotka, L. Shen, D. M. Skovronsky, BLAZE-1 Investigators, Bamlanivimab plus Etesevimab in Mild or Moderate Covid-19. *N. Engl. J. Med.* **385**, 1382–1392 (2021).

3. A. Gupta, Y. Gonzalez-Rojas, E. Juarez, M. Crespo Casal, J. Moya, D. R. Falci, E. Sarkis, J. Solis, H. Zheng, N. Scott, A. L. Cathcart, C. M. Hebner, J. Sager, E. Mogalian, C. Tipple, A. Peppercorn, E. Alexander, P. S. Pang, A. Free, C. Brinson, M. Aldinger, A. E. Shapiro, COMET-ICE Investigators, Early Treatment for Covid-19 with SARS-CoV-2 Neutralizing Antibody Sotrovimab. *N. Engl. J. Med.* **385**, 1941–1950 (2021).

4. D. M. Weinreich, S. Sivapalasingam, T. Norton, S. Ali, H. Gao, R. Bhore, J. Xiao, A. T. Hooper, J. D. Hamilton, B. J. Musser, D. Rofail, M. Hussein, J. Im, D. Y. Atmodjo, C. Perry, C. Pan, A. Mahmood, R. Hosain, J. D. Davis, K. C. Turner, A. Baum, C. A. Kyratsous, Y. Kim, A. Cook, W. Kampman, L. Roque-Guerrero, G. Acloque, H. Aazami, K. Cannon, J. A. Simón-Campos, J. A. Bocchini, B. Kowal, A. T. DiCioccio, Y. Soo, G. P. Geba, N. Stahl, L. Lipsich, N. Braunstein, G. Herman, G. D. Yancopoulos, Trial Investigators, REGEN-COV Antibody Combination and Outcomes in Outpatients with Covid-19. *N. Engl. J. Med.* **385**, e81 (2021).

5. D. Corti, L. A. Purcell, G. Snell, D. Veesler, Tackling COVID-19 with neutralizing monoclonal antibodies. *Cell* **184**, 4593–4595 (2021).

6. Z. Liu, L. A. VanBlargan, L.-M. Bloyet, P. W. Rothlauf, R. E. Chen, S. Stumpf, H. Zhao, J. M. Errico, E. S. Theel, M. J. Liebeskind, B. Alford, W. J. Buchser, A. H. Ellebedy, D. H. Fremont, M. S. Diamond, S. P. J. Whelan, Identification of SARS-CoV-2 spike mutations that attenuate monoclonal and serum antibody neutralization. *Cell Host Microbe* **29**, 477-488.e4 (2021).

7. T. N. Starr, A. J. Greaney, A. Addetia, W. W. Hannon, M. C. Choudhary, A. S. Dingens, J. Z. Li, J. D. Bloom, Prospective mapping of viral mutations that escape antibodies used to treat COVID-19. *Science* **371**, 850–854 (2021).

8. Z. R. Crook, N. W. Nairn, J. M. Olson, Miniproteins as a Powerful Modality in Drug Development. *Trends Biochem. Sci.* **45**, 332–346 (2020).

9. M. McCallum, A. C. Walls, K. R. Sprouse, J. E. Bowen, L. E. Rosen, H. V. Dang, A. De Marco, N. Franko, S. W. Tilles, J. Logue, M. C. Miranda, M. Ahlrichs, L. Carter, G. Snell, M. S. Pizzuto, H. Y. Chu, W. C. Van Voorhis, D. Corti, D. Veesler, Molecular basis of immune evasion by the Delta and Kappa SARS-CoV-2 variants. *Science* **374**,

1621–1626 (2021).

10. M. McCallum, J. Bassi, A. De Marco, A. Chen, A. C. Walls, J. Di Iulio, M. A. Tortorici, M.-J. Navarro, C. Silacci-Fregni, C. Saliba, K. R. Sprouse, M. Agostini, D. Pinto, K. Culap, S. Bianchi, S. Jaconi, E. Cameroni, J. E. Bowen, S. W. Tilles, M. S. Pizzuto, S. B. Guastalla, G. Bona, A. F. Pellanda, C. Garzoni, W. C. Van Voorhis, L. E. Rosen, G. Snell, A. Telenti, H. W. Virgin, L. Piccoli, D. Corti, D. Veesler, SARS-CoV-2 immune evasion by the B.1.427/B.1.429 variant of concern. *Science* **373**, 648–654 (2021).

11. W. Dejnirattisai, D. Zhou, P. Supasa, C. Liu, A. J. Mentzer, H. M. Ginn, Y. Zhao, H. M. E. Duyvesteyn, A. Tuekprakhon, R. Nutalai, B. Wang, C. López-Camacho, J. Slon-Campos, T. S. Walter, D. Skelly, S. A. Costa Clemens, F. G. Naveca, V. Nascimento, F. Nascimento, C. Fernandes da Costa, P. C. Resende, A. Pauvolid-Correa, M. M. Siqueira, C. Dold, R. Levin, T. Dong, A. J. Pollard, J. C. Knight, D. Crook, T. Lambe, E. Clutterbuck, S. Bibi, A. Flaxman, M. Bittaye, S. Belij-Rammerstorfer, S. C. Gilbert, M. W. Carroll, P. Klenerman, E. Barnes, S. J. Dunachie, N. G. Paterson, M. A. Williams, D. R. Hall, R. J. G. Hulswit, T. A. Bowden, E. E. Fry, J. Mongkolsapaya, J. Ren, D. I. Stuart, G. R. Screaton, Antibody evasion by the P.1 strain of SARS-CoV-2. *Cell* **184**, 2939-2954.e9 (2021).

12. R. E. Chen, X. Zhang, J. B. Case, E. S. Winkler, Y. Liu, L. A. VanBlargan, J. Liu, J. M. Errico, X. Xie, N. Suryadevara, P. Gilchuk, S. J. Zost, S. Tahan, L. Droit, J. S. Turner, W. Kim, A. J. Schmitz, M. Thapa, D. Wang, A. C. M. Boon, R. M. Presti, J. A. O'Halloran, A. H. J. Kim, P. Deepak, D. Pinto, D. H. Fremont, J. E. Crowe Jr, D. Corti, H. W. Virgin, A. H. Ellebedy, P.-Y. Shi, M. S. Diamond, Resistance of SARS-CoV-2 variants to neutralization by monoclonal and serum-derived polyclonal antibodies. *Nat. Med.* **27**, 717–726 (2021).

13. R. E. Chen, E. S. Winkler, J. B. Case, I. D. Aziati, T. L. Bricker, A. Joshi, T. L. Darling, B. Ying, J. M. Errico, S. Shrihari, L. A. VanBlargan, X. Xie, P. Gilchuk, S. J. Zost, L. Droit, Z. Liu, S. Stumpf, D. Wang, S. A. Handley, W. B. Stine Jr, P.-Y. Shi, M. E. Davis-Gardner, M. S. Suthar, M. G. Knight, R. Andino, C. Y. Chiu, A. H. Ellebedy, D. H. Fremont, S. P. J. Whelan, J. E. Crowe Jr, L. Purcell, D. Corti, A. C. M. Boon, M. S. Diamond, In vivo monoclonal antibody efficacy against SARS-CoV-2 variant strains. *Nature* **596**, 103–108 (2021).

14. D. A. Collier, A. De Marco, I. A. T. M. Ferreira, B. Meng, R. Datir, A. C. Walls, S. A. Kemp S, J. Bassi, D. Pinto, C. S. Fregni, S. Bianchi, M. A. Tortorici, J. Bowen, K. Culap, S. Jaconi, E. Cameroni, G. Snell, M. S. Pizzuto, A. F. Pellanda, C. Garzoni, A. Riva, A. Elmer, N. Kingston, B. Graves, L. E. McCoy, K. G. C. Smith, J. R. Bradley, N. Temperton, L. Lourdes Ceron-Gutierrez, G. Barcenas-Morales, W. Harvey, H. W. Virgin, A. Lanzavecchia, L. Piccoli, R. Doffinger, M. Wills, D. Veesler, D. Corti, R. K. Gupta, Sensitivity of SARS-CoV-2 B.1.1.7 to mRNA vaccine-elicited antibodies. *Nature* **593**, 136–141 (2021).

15. E. Cameroni, J. E. Bowen, L. E. Rosen, C. Saliba, S. K. Zepeda, K. Culap, D. Pinto, L. A. VanBlargan, A. De Marco, J. di Iulio, F. Zatta, H. Kaiser, J. Noack, N. Farhat, N. Czudnochowski, C. Havenar-Daughton, K. R. Sprouse, J. R. Dillen, A. E. Powell, A.

Chen, C. Maher, L. Yin, D. Sun, L. Soriaga, J. Bassi, C. Silacci-Fregni, C. Gustafsson, N. M. Franko, J. Logue, N. T. Iqbal, I. Mazzitelli, J. Geffner, R. Grifantini, H. Chu, A. Gori, A. Riva, O. Giannini, A. Ceschi, P. Ferrari, P. E. Cippà, A. Franzetti-Pellanda, C. Garzoni, P. J. Halfmann, Y. Kawaoka, C. Hebner, L. A. Purcell, L. Piccoli, M. S. Pizzuto, A. C. Walls, M. S. Diamond, A. Telenti, H. W. Virgin, A. Lanzavecchia, G. Snell, D. Veesler, D. Corti, Broadly neutralizing antibodies overcome SARS-CoV-2 Omicron antigenic shift. *Nature* **602**, 664–670 (2021).

16. L. VanBlargan, J. Errico, P. Halfmann, S. Zost, J. Crowe, L. Purcell, Y. Kawaoka, D. Corti, D. Fremont, M. Diamond, An infectious SARS-CoV-2 B.1.1.529 Omicron virus escapes neutralization by therapeutic monoclonal antibodies. *Nature Medicine* **28**, 490–495 (2022).

17. M. McCallum, N. Czudnochowski, L. E. Rosen, S. K. Zepeda, J. E. Bowen, A. C. Walls, K. Hauser, A. Joshi, C. Stewart, J. R. Dillen, A. E. Powell, T. I. Croll, J. Nix, H. W. Virgin, D. Corti, G. Snell, D. Veesler, Structural basis of SARS-CoV-2 Omicron immune evasion and receptor engagement. *Science* **375**, 864–868 (2022).

18. J. B. Case, S. Mackin, J. Errico, Z. Chong, E. A. Madden, B. Guarino, M. A. Schmid, K. Rosenthal, K. Ren, A. Jung, L. Droit, S. A. Handley, P. J. Halfmann, Y. Kawaoka, J. E. Crowe Jr, D. H. Fremont, H. W. Virgin, Y.-M. Loo, M. T. Esser, L. A. Purcell, D. Corti, M. S. Diamond, Resilience of S309 and AZD7442 monoclonal antibody treatments against infection by SARS-CoV-2 Omicron lineage strains. *bioRxiv* , 2022.03.17.484787 (2022).

19. D. Pinto, Y. J. Park, M. Beltramello, A. C. Walls, M. A. Tortorici, S. Bianchi, S. Jaconi, K. Culap, F. Zatta, A. De Marco, A. Peter, B. Guarino, R. Spreafico, E. Cameroni, J. B. Case, R. E. Chen, C. Havenar-Daughton, G. Snell, A. Telenti, H. W. Virgin, A. Lanzavecchia, M. S. Diamond, K. Fink, D. Veesler, D. Corti, Cross-neutralization of SARS-CoV-2 by a human monoclonal SARS-CoV antibody. *Nature* **583**, 290–295 (2020).

20. D. Pinto, M. M. Sauer, N. Czudnochowski, J. S. Low, M. A. Tortorici, M. P. Housley, J. Noack, A. C. Walls, J. E. Bowen, B. Guarino, L. E. Rosen, J. di Iulio, J. Jerak, H. Kaiser, S. Islam, S. Jaconi, N. Sprugasci, K. Culap, R. Abdelnabi, C. Foo, L. Coelmont, I. Bartha, S. Bianchi, C. Silacci-Fregni, J. Bassi, R. Marzi, E. Vetti, A. Cassotta, A. Ceschi, P. Ferrari, P. E. Cippà, O. Giannini, S. Ceruti, C. Garzoni, A. Riva, F. Benigni, E. Cameroni, L. Piccoli, M. S. Pizzuto, M. Smithey, D. Hong, A. Telenti, F. A. Lempp, J. Neyts, C. Havenar-Daughton, A. Lanzavecchia, F. Sallusto, G. Snell, H. W. Virgin, M. Beltramello, D. Corti, D. Veesler, Broad betacoronavirus neutralization by a stem helix-specific human antibody. *Science* **373**, 1109–1116 (2021).

21. T. N. Starr, N. Czudnochowski, Z. Liu, F. Zatta, Y.-J. Park, A. Addetia, D. Pinto, M. Beltramello, P. Hernandez, A. J. Greaney, R. Marzi, W. G. Glass, I. Zhang, A. S. Dingens, J. E. Bowen, M. A. Tortorici, A. C. Walls, J. A. Wojcechowskyj, A. De Marco, L. E. Rosen, J. Zhou, M. Montiel-Ruiz, H. Kaiser, J. R. Dillen, H. Tucker, J. Bassi, C. Silacci-Fregni, M. P. Housley, J. di Iulio, G. Lombardo, M. Agostini, N. Sprugasci, K. Culap, S. Jaconi, M. Meury, E. Dellota Jr, R. Abdelnabi, S.-Y. C. Foo, E. Cameroni, S. Stumpf, T. I. Croll, J. C. Nix, C. Havenar-Daughton, L. Piccoli, F. Benigni, J. Neyts, A.

Telenti, F. A. Lempp, M. S. Pizzuto, J. D. Chodera, C. M. Hebner, H. W. Virgin, S. P. J. Whelan, D. Veesler, D. Corti, J. D. Bloom, G. Snell, SARS-CoV-2 RBD antibodies that maximize breadth and resistance to escape. *Nature* **597**, 97–102 (2021).

22. C. A. Jette, A. A. Cohen, P. N. P. Gnanapragasam, F. Muecksch, Y. E. Lee, K. E. Huey-Tubman, F. Schmidt, T. Hatziioannou, P. D. Bieniasz, M. C. Nussenzweig, A. P. West Jr, J. R. Keeffe, P. J. Bjorkman, C. O. Barnes, Broad cross-reactivity across sarbecoviruses exhibited by a subset of COVID-19 donor-derived neutralizing antibodies. *Cell Rep.* **36**, 109760 (2021).

23. L. A. VanBlargan, L. J. Adams, Z. Liu, R. E. Chen, P. Gilchuk, S. Raju, B. K. Smith, H. Zhao, J. B. Case, E. S. Winkler, B. M. Whitener, L. Droit, I. D. Aziati, T. L. Bricker, A. Joshi, P.-Y. Shi, A. Creanga, A. Pegu, S. A. Handley, D. Wang, A. C. M. Boon, J. E. Crowe Jr, S. P. J. Whelan, D. H. Fremont, M. S. Diamond, A potently neutralizing SARS-CoV-2 antibody inhibits variants of concern by utilizing unique binding residues in a highly conserved epitope. *Immunity* **54**, 2399-2416.e6 (2021).

24. Y.-J. Park, A. De Marco, T. N. Starr, Z. Liu, D. Pinto, A. C. Walls, F. Zatta, S. K. Zepeda, J. E. Bowen, K. R. Sprouse, A. Joshi, M. Giurdanella, B. Guarino, J. Noack, R. Abdelnabi, S.-Y. C. Foo, L. E. Rosen, F. A. Lempp, F. Benigni, G. Snell, J. Neyts, S. P. J. Whelan, H. W. Virgin, J. D. Bloom, D. Corti, M. S. Pizzuto, D. Veesler, Antibody-mediated broad sarbecovirus neutralization through ACE2 molecular mimicry. *Science* **375**, 449–454 (2022).

25. L. Cao, I. Goreshnik, B. Coventry, J. B. Case, L. Miller, L. Kozodoy, R. E. Chen, L. Carter, A. C. Walls, Y.-J. Park, E.-M. Strauch, L. Stewart, M. S. Diamond, D. Veesler, D. Baker, De novo design of picomolar SARS-CoV-2 miniprotein inhibitors. *Science* **370**, 426–431 (2020).

26. J. B. Case, R. E. Chen, L. Cao, B. Ying, E. S. Winkler, M. Johnson, I. Goreshnik, M. N. Pham, S. Shrihari, N. M. Kafai, A. L. Bailey, X. Xie, P.-Y. Shi, R. Ravichandran, L. Carter, L. Stewart, D. Baker, M. S. Diamond, Ultrapotent miniproteins targeting the SARS-CoV-2 receptor-binding domain protect against infection and disease. *Cell Host Microbe* **29**, 1151-1161.e5 (2021).

27. K. Javanmardi, C.-W. Chou, C. I. Terrace, A. Annapareddy, T. S. Kaoud, Q. Guo, J. Lutgens, H. Zorkic, A. P. Horton, E. C. Gardner, G. Nguyen, D. R. Boutz, J. Goike, W. N. Voss, H.-C. Kuo, K. N. Dalby, J. D. Gollihar, I. J. Finkelstein, Rapid characterization of spike variants via mammalian cell surface display. *Molecular Cell* **81**, P5099-5111.E8 (2021).

28. J. Silverman, Q. Liu, A. Bakker, W. To, A. Duguay, B. M. Alba, R. Smith, A. Rivas, P. Li, H. Le, E. Whitehorn, K. W. Moore, C. Swimmer, V. Perlroth, M. Vogt, J. Kolkman, W. P. C. Stemmer, Multivalent avimer proteins evolved by exon shuffling of a family of human receptor domains. *Nat. Biotechnol.* **23**, 1556–1561 (2005).

29. L. Detalle, T. Stohr, C. Palomo, P. A. Piedra, B. E. Gilbert, V. Mas, A. Millar, U. F. Power, C. Stortelers, K. Allosery, J. A. Melero, E. Depla, Generation and Characterization of ALX-0171, a Potent Novel Therapeutic Nanobody for the Treatment

of Respiratory Syncytial Virus Infection. *Antimicrob. Agents Chemother.* **60**, 6–13 (2016).

30. E. M. Strauch, S. M. Bernard, D. La, A. J. Bohn, P. S. Lee, C. E. Anderson, T. Nieusma, C. A. Holstein, N. K. Garcia, K. A. Hooper, R. Ravichandran, J. W. Nelson, W. Sheffler, J. D. Bloom, K. K. Lee, A. B. Ward, P. Yager, D. H. Fuller, I. A. Wilson, D. Baker, Computational design of trimeric influenza-neutralizing proteins targeting the hemagglutinin receptor binding site. *Nat. Biotechnol.* **35**, 667–671 (2017).

31. C. J. Bracken, S. A. Lim, P. Solomon, N. J. Rettko, D. P. Nguyen, B. S. Zha, K. Schaefer, J. R. Byrnes, J. Zhou, I. Lui, J. Liu, K. Pance, QCRG Structural Biology Consortium, X. X. Zhou, K. K. Leung, J. A. Wells, Bi-paratopic and multivalent VH domains block ACE2 binding and neutralize SARS-CoV-2. *Nat. Chem. Biol.* **17**, 113–121 (2021).

32. P.-A. Koenig, H. Das, H. Liu, B. M. Kümmerer, F. N. Gohr, L.-M. Jenster, L. D. J. Schiffelers, Y. M. Tesfamariam, M. Uchima, J. D. Wuerth, K. Gatterdam, N. Ruetalo, M. H. Christensen, C. I. Fandrey, S. Normann, J. M. P. Tödtmann, S. Pritzl, L. Hanke, J. Boos, M. Yuan, X. Zhu, J. L. Schmid-Burgk, H. Kato, M. Schindler, I. A. Wilson, M. Geyer, K. U. Ludwig, B. M. Hällberg, N. C. Wu, F. I. Schmidt, Structure-guided multivalent nanobodies block SARS-CoV-2 infection and suppress mutational escape. *Science* **371**, eabe6230 (2021).

33. Y. Xiang, S. Nambulli, Z. Xiao, H. Liu, Z. Sang, W. P. Duprex, D. Schneidman-Duhovny, C. Zhang, Y. Shi, Versatile and multivalent nanobodies efficiently neutralize SARS-CoV-2. *Science* **370**, 1479–1484 (2020).

34. M. Walser, S. Rothenberger, D. L. Hurdiss, A. Schlegel, V. Calabro, S. Fontaine, D. Villemagne, M. Paladino, T. Hospodarsch, A. Neculcea, A. Cornelius, P. Schildknecht, M. Matzner, M. Hänggi, M. Franchini, Y. Kaufmann, D. Schaible, I. Schlegel, C. Iss, T. Looser, S. Mangold, C. Herzog, D. Schiegg, C. Reichen, F. Radom, A. Bosshart, A. Lehmann, M. A. Haeuptle, A. Zürcher, T. Vagt, G. Sigrist, M. Straumann, K. Proba, N. Veitonmäki, K. M. Dawson, C. Zitt, J. Mayor, S. Ryter, H. Lyoo, C. Wang, W. Li, I. Drulyte, W. Du, H. Kaspar Binz, L. de Waal, K. J. Stittelaar, S. Taplin, S. Lewis, D. Steiner, F. J. M. van Kuppeveld, O. Engler, B.-J. Bosch, M. T. Stumpp, P. Amstutz, Highly potent anti-SARS-CoV-2 multi-DARPin therapeutic candidates. *bioRxiv* , 2020.08.25.256339 (2020).

35. M. Schoof, B. Faust, R. A. Saunders, S. Sangwan, V. Rezelj, N. Hoppe, M. Boone, C. B. Billesbølle, C. Puchades, C. M. Azumaya, H. T. Kratochvil, M. Zimanyi, I. Deshpande, J. Liang, S. Dickinson, H. C. Nguyen, C. M. Chio, G. E. Merz, M. C. Thompson, D. Diwanji, K. Schaefer, A. A. Anand, N. Dobzinski, B. S. Zha, C. R. Simoneau, K. Leon, K. M. White, U. S. Chio, M. Gupta, M. Jin, F. Li, Y. Liu, K. Zhang, D. Bulkley, M. Sun, A. M. Smith, A. N. Rizo, F. Moss, A. F. Brilot, S. Pourmal, R. Trenker, T. Pospiech, S. Gupta, B. Barsi-Rhyne, V. Belyy, A. W. Barile-Hill, S. Nock, Y. Liu, N. J. Krogan, C. Y. Ralston, D. L. Swaney, A. García-Sastre, M. Ott, M. Vignuzzi, QCRG Structural Biology Consortium, P. Walter, A. Manglik, An ultrapotent synthetic nanobody neutralizes SARS-CoV-2 by stabilizing inactive Spike. *Science* **370**, 1473–1479 (2020).

36. T. W. Linsky, R. Vergara, N. Codina, J. W. Nelson, M. J. Walker, W. Su, C. O. Barnes, T.-Y. Hsiang, K. Esser-Nobis, K. Yu, Z. B. Reneer, Y. J. Hou, T. Priya, M. Mitsumoto, A. Pong, U. Y. Lau, M. L. Mason, J. Chen, A. Chen, T. Berrocal, H. Peng, N. S. Clairmont, J. Castellanos, Y.-R. Lin, A. Josephson-Day, R. S. Baric, D. H. Fuller, C. D. Walkey, T. M. Ross, R. Swanson, P. J. Bjorkman, M. Gale Jr, L. M. Blancas-Mejia, H.-L. Yen, D.-A. Silva, De novo design of potent and resilient hACE2 decoys to neutralize SARS-CoV-2. *Science* **370**, 1208–1214 (2020).

37. R. Copin, A. Baum, E. Wloga, K. E. Pascal, S. Giordano, B. O. Fulton, A. Zhou, N. Negron, K. Lanza, N. Chan, A. Coppola, J. Chiu, M. Ni, Y. Wei, G. S. Atwal, A. R. Hernandez, K. Saotome, Y. Zhou, M. C. Franklin, A. T. Hooper, S. McCarthy, S. Hamon, J. D. Hamilton, H. M. Staples, K. Alfson, R. Carrion, S. Ali, T. Norton, S. Somersan-Karakaya, S. Sivapalasingam, G. A. Herman, D. M. Weinreich, L. Lipsich, N. Stahl, A. J. Murphy, G. D. Yancopoulos, C. A. Kyratsous, The monoclonal antibody combination REGEN-COV protects against SARS-CoV-2 mutational escape in preclinical and human studies. *Cell* **184**, 3949-3961.e11 (2021).

38. T. N. Starr, A. J. Greaney, S. K. Hilton, D. Ellis, K. H. D. Crawford, A. S. Dingens, M. J. Navarro, J. E. Bowen, M. A. Tortorici, A. C. Walls, N. P. King, D. Veesler, J. D. Bloom, Deep Mutational Scanning of SARS-CoV-2 Receptor Binding Domain Reveals Constraints on Folding and ACE2 Binding. *Cell* **182**, 1295-1310.e20 (2020).

39. C. Palomo, V. Mas, L. Detalle, E. Depla, O. Cano, M. Vázquez, C. Stortelers, J. A. Melero, Trivalency of a Nanobody Specific for the Human Respiratory Syncytial Virus Fusion Glycoprotein Drastically Enhances Virus Neutralization and Impacts Escape Mutant Selection. *Antimicrob. Agents Chemother.* **60**, 6498–6509 (2016).

40. Z. Z. Sun, E. Yeung, C. A. Hayes, V. Noireaux, R. M. Murray, Linear DNA for rapid prototyping of synthetic biological circuits in an Escherichia coli based TX-TL cell-free system. *ACS Synth. Biol.* **3**, 387–397 (2014).

41. A. D. Silverman, A. S. Karim, M. C. Jewett, Cell-free gene expression: an expanded repertoire of applications. *Nat. Rev. Genet.* **21**, 151–170 (2020).

42. C.-L. Hsieh, J. A. Goldsmith, J. M. Schaub, A. M. DiVenere, H.-C. Kuo, K. Javanmardi, K. C. Le, D. Wrapp, A. G. Lee, Y. Liu, C.-W. Chou, P. O. Byrne, C. K. Hjorth, N. V. Johnson, J. Ludes-Meyers, A. W. Nguyen, J. Park, N. Wang, D. Amengor, J. J. Lavinder, G. C. Ippolito, J. A. Maynard, I. J. Finkelstein, J. S. McLellan, Structure-based design of prefusion-stabilized SARS-CoV-2 spikes. *Science* **369**, 1501–1505 (2020).

43. M. Mammen, S.-K. Choi, G. M. Whitesides, Polyvalent Interactions in Biological Systems: Implications for Design and Use of Multivalent Ligands and Inhibitors. *Angew. Chem. Int. Ed Engl.* **37**, 2754–2794 (1998).

44. T. D. Pollard, E. M. De La Cruz, Take advantage of time in your experiments: a guide to simple, informative kinetics assays. *Mol. Biol. Cell* **24**, 1103–1110 (2013).

45. S. Güthe, L. Kapinos, A. Möglich, S. Meier, S. Grzesiek, T. Kiefhaber, Very fast folding and association of a trimerization domain from bacteriophage T4 fibritin. *J. Mol.*

Biol. **337**, 905–915 (2004).

46. A. Gräwe, V. Stein, Linker Engineering in the Context of Synthetic Protein Switches and Sensors. *Trends Biotechnol.* **39**, 731–744 (2020).

47. Z. Ke, J. Oton, K. Qu, M. Cortese, V. Zila, L. McKeane, T. Nakane, J. Zivanov, C. J. Neufeldt, B. Cerikan, J. M. Lu, J. Peukes, X. Xiong, H. G. Kräusslich, S. H. W. Scheres, R. Bartenschlager, J. A. G. Briggs, Structures and distributions of SARS-CoV-2 spike proteins on intact virions. *Nature* **588**, 498–502 (2020).

48. B. Turoňová, M. Sikora, C. Schürmann, W. J. H. Hagen, S. Welsch, F. E. C. Blanc, S. von Bülow, M. Gecht, K. Bagola, C. Hörner, G. van Zandbergen, J. Landry, N. T. D. de Azevedo, S. Mosalaganti, A. Schwarz, R. Covino, M. D. Mühlebach, G. Hummer, J. Krijnse Locker, M. Beck, In situ structural analysis of SARS-CoV-2 spike reveals flexibility mediated by three hinges. *Science* **370**, 203–208 (2020).

49. H. Yao, Y. Song, Y. Chen, N. Wu, J. Xu, C. Sun, J. Zhang, T. Weng, Z. Zhang, Z. Wu, L. Cheng, D. Shi, X. Lu, J. Lei, M. Crispin, Y. Shi, L. Li, S. Li, Molecular Architecture of the SARS-CoV-2 Virus. *Cell* **183**, 730-738.e13 (2020).

50. D. Wrapp, N. Wang, K. S. Corbett, J. A. Goldsmith, C.-L. Hsieh, O. Abiona, B. S. Graham, J. S. McLellan, Cryo-EM structure of the 2019-nCoV spike in the prefusion conformation. *Science* **367**, 1260–1263 (2020).

51. H. W. Yeh, O. Karmach, A. Ji, D. Carter, M. M. Martins-Green, H. W. Ai, Red-shifted luciferase-luciferin pairs for enhanced bioluminescence imaging. *Nat. Methods* **14**, 971–974 (2017).

52. B. B. Kim, H. Wu, Y. A. Hao, M. Pan, M. Chavarha, Y. Zhao, M. Westberg, F. St-Pierre, J. C. Wu, M. Z. Lin, A red fluorescent protein with improved monomericity enables ratiometric voltage imaging with ASAP3. *Scientific Rep* **12**, 3678 (2020).

53. L. Piccoli, Y. J. Park, M. A. Tortorici, N. Czudnochowski, A. C. Walls, M. Beltramello, C. Silacci-Fregni, D. Pinto, L. E. Rosen, J. E. Bowen, O. J. Acton, S. Jaconi, B. Guarino, A. Minola, F. Zatta, N. Sprugasci, J. Bassi, A. Peter, A. De Marco, J. C. Nix, F. Mele, S. Jovic, B. F. Rodriguez, S. V. Gupta, F. Jin, G. Piumatti, G. Lo Presti, A. F. Pellanda, M. Biggiogero, M. Tarkowski, M. S. Pizzuto, E. Cameroni, C. Havenar-Daughton, M. Smithey, D. Hong, V. Lepori, E. Albanese, A. Ceschi, E. Bernasconi, L. Elzi, P. Ferrari, C. Garzoni, A. Riva, G. Snell, F. Sallusto, K. Fink, H. W. Virgin, A. Lanzavecchia, D. Corti, D. Veesler, Mapping Neutralizing and Immunodominant Sites on the SARS-CoV-2 Spike Receptor-Binding Domain by Structure-Guided High-Resolution Serology. *Cell* **183**, 1024-1042.e21 (2020).

54. J. Beumer, M. H. Geurts, M. M. Lamers, J. Puschhof, J. Zhang, J. van der Vaart, A. Z. Mykytyn, T. I. Breugem, S. Riesebosch, D. Schipper, P. B. van den Doel, W. de Lau, C. Pleguezuelos-Manzano, G. Busslinger, B. L. Haagmans, H. Clevers, A CRISPR/Cas9 genetically engineered organoid biobank reveals essential host factors for coronaviruses. *Nat. Commun.* **12**, 5498 (2021).

55. J. M. Penninger, A. Mirazimi, N. Montserrat, Inhibition of SARS-CoV-2 infections in

engineered human tissues using clinical-grade soluble human ACE2. *Cell* **181**, 905-913.e7 (2020).

56. J. L. Harder, R. Menon, E. A. Otto, J. Zhou, S. Eddy, N. L. Wys, C. O'Connor, J. Luo, V. Nair, C. Cebrian, J. R. Spence, M. Bitzer, O. G. Troyanskaya, J. B. Hodgin, R. C. Wiggins, B. S. Freedman, M. Kretzler, European Renal cDNA Bank (ERCB), Nephrotic Syndrome Study Network (NEPTUNE), Organoid single cell profiling identifies a transcriptional signature of glomerular disease. *JCI Insight* **4** (2019), doi:10.1172/jci.insight.122697.

57. B. S. Freedman, C. R. Brooks, A. Q. Lam, H. Fu, R. Morizane, V. Agrawal, A. F. Saad, M. K. Li, M. R. Hughes, R. V. Werff, D. T. Peters, J. Lu, A. Baccei, A. M. Siedlecki, M. T. Valerius, K. Musunuru, K. M. McNagny, T. I. Steinman, J. Zhou, P. H. Lerou, J. V. Bonventre, Modelling kidney disease with CRISPR-mutant kidney organoids derived from human pluripotent epiblast spheroids. *Nat. Commun.* **6**, 8715 (2015).

58. W. B. Alsoussi, J. S. Turner, J. B. Case, H. Zhao, A. J. Schmitz, J. Q. Zhou, R. E. Chen, T. Lei, A. A. Rizk, K. M. McIntire, E. S. Winkler, J. M. Fox, N. M. Kafai, L. B. Thackray, A. O. Hassan, F. Amanat, F. Krammer, C. T. Watson, S. H. Kleinstein, D. H. Fremont, M. S. Diamond, A. H. Ellebedy, A Potently Neutralizing Antibody Protects Mice against SARS-CoV-2 Infection. *The Journal of Immunology* **205**, 915–922 (2020).

59. J. M. Errico, H. Zhao, R. E. Chen, Z. Liu, J. B. Case, M. Ma, A. J. Schmitz, M. J. Rau, J. A. J. Fitzpatrick, P.-Y. Shi, M. S. Diamond, S. P. J. Whelan, A. H. Ellebedy, D. H. Fremont, Structural mechanism of SARS-CoV-2 neutralization by two murine antibodies targeting the RBD. *Cell Rep.* **37**, 109881 (2021).

60. W. Su, S. F. Sia, A. J. Schmitz, T. L. Bricker, T. N. Starr, A. J. Greaney, J. S. Turner, B. M. Mohammed, Z. Liu, K. T. Choy, T. L. Darling, A. Joshi, K. M. Cheng, A. Y. L. Wong, H. H. Harastani, J. M. Nicholls, S. P. J. Whelan, J. D. Bloom, H.-L. Yen, A. H. Ellebedy, A. C. M. Boon, Neutralizing Monoclonal Antibodies That Target the Spike Receptor Binding Domain Confer Fc Receptor-Independent Protection against SARS-CoV-2 Infection in Syrian Hamsters. *MBio* **12**, e0239521 (2021).

61. G. W. Wertz, R. Moudy, L. A. Ball, Adding genes to the RNA genome of vesicular stomatitis virus: positional effects on stability of expression. *J. Virol.* **76**, 7642–7650 (2002).

62. A. O. Hassan, J. B. Case, E. S. Winkler, L. Thackray, M. Kafai, A. L. Bailey, B. T. Mccune, J. M. Fox, R. E. Chen, W. B. Al, J. S. Turner, A. J. Schmitz, T. Lei, S. Shrihari, P. Keeler, D. H. Fremont, S. Greco, P. B. Mccray, S. Perlman, M. J. Holtzman, A. H. Ellebedy, M. S. Diamond, A SARS-CoV-2 infection model in mice demonstrates protection by neutralizing antibodies. *Cell* **182**, 744-753.e4 (2020).

63. A. C. Walls, X. Xiong, Y. J. Park, M. A. Tortorici, J. Snijder, J. Quispe, E. Cameroni, R. Gopal, M. Dai, A. Lanzavecchia, M. Zambon, F. A. Rey, D. Corti, D. Veesler, Unexpected Receptor Functional Mimicry Elucidates Activation of Coronavirus Fusion. *Cell* **176**, 1026-1039.e15 (2019).

64. F. A. Lempp, L. B. Soriaga, M. Montiel-Ruiz, F. Benigni, J. Noack, Y.-J. Park, S. Bianchi, A. C. Walls, J. E. Bowen, J. Zhou, H. Kaiser, A. Joshi, M. Agostini, M. Meury, E. Dellota Jr, S. Jaconi, E. Cameroni, J. Martinez-Picado, J. Vergara-Alert, N. Izquierdo-Useros, H. W. Virgin, A. Lanzavecchia, D. Veesler, L. A. Purcell, A. Telenti, D. Corti, Lectins enhance SARS-CoV-2 infection and influence neutralizing antibodies. *Nature* **598**, 342–347 (2021).

65. L. Guo, W. Bi, X. Wang, W. Xu, R. Yan, Y. Zhang, K. Zhao, Y. Li, M. Zhang, X. Cai, S. Jiang, Y. Xie, Q. Zhou, L. Lu, B. Dang, Engineered trimeric ACE2 binds viral spike protein and locks it in “Three-up” conformation to potently inhibit SARS-CoV-2 infection. *Cell Res.* **31**, 98–100 (2021).

66. A. Glasgow, J. Glasgow, D. Limonta, P. Solomon, I. Lui, Y. Zhang, M. A. Nix, N. J. Rettko, S. Zha, R. Yamin, K. Kao, O. S. Rosenberg, J. V. Ravetch, A. P. Wiita, K. K. Leung, S. A. Lim, X. X. Zhou, T. C. Hobman, T. Kortemme, J. A. Wells, Engineered ACE2 receptor traps potently neutralize SARS-CoV-2. *Proc. Natl. Acad. Sci. U. S. A.* **117**, 28046–28055 (2020).

67. Y. Higuchi, T. Suzuki, T. Arimori, N. Ikemura, E. Mihara, Y. Kirita, E. Ohgitani, O. Mazda, D. Motooka, S. Nakamura, Y. Sakai, Y. Itoh, F. Sugihara, Y. Matsuura, S. Matoba, T. Okamoto, J. Takagi, A. Hoshino, Engineered ACE2 receptor therapy overcomes mutational escape of SARS-CoV-2. *Nat. Commun.* **12**, 3802 (2021).

68. D. M. Weinreich, S. Sivapalasingam, T. Norton, S. Ali, H. Gao, R. Bhore, B. J. Musser, Y. Soo, D. Rofail, J. Im, C. Perry, C. Pan, R. Hosain, A. Mahmood, J. D. Davis, K. C. Turner, A. T. Hooper, J. D. Hamilton, A. Baum, C. A. Kyratsous, Y. Kim, A. Cook, W. Kampman, A. Kohli, Y. Sachdeva, X. Graber, B. Kowal, T. DiCioccio, N. Stahl, L. Lipsich, N. Braunstein, G. Herman, G. D. Yancopoulos, REGN-COV2, a Neutralizing Antibody Cocktail, in Outpatients with Covid-19. *N. Engl. J. Med.* **384**, 238–251 (2021).

69. P. Chen, A. Nirula, B. Heller, R. L. Gottlieb, J. Boscia, J. Morris, G. Huhn, J. Cardona, B. Mocherla, V. Stosor, I. Shawa, A. C. Adams, J. Van Naarden, K. L. Custer, L. Shen, M. Durante, G. Oakley, A. E. Schade, J. Sabo, D. R. Patel, P. Klekotka, D. M. Skovronsky, SARS-CoV-2 Neutralizing Antibody LY-CoV555 in Outpatients with Covid-19. *N. Engl. J. Med.* **384**, 229–237 (2020).

70. M. P. O’Brien, E. Forleo-Neto, B. J. Musser, F. Isa, K.-C. Chan, N. Sarkar, K. J. Bar, R. V. Barnabas, D. H. Barouch, M. S. Cohen, C. B. Hurt, D. R. Burwen, M. A. Marovich, P. Hou, I. Heirman, J. D. Davis, K. C. Turner, D. Ramesh, A. Mahmood, A. T. Hooper, J. D. Hamilton, Y. Kim, L. A. Purcell, A. Baum, C. A. Kyratsous, J. Krainson, R. Perez-Perez, R. Mohseni, B. Kowal, A. T. DiCioccio, N. Stahl, L. Lipsich, N. Braunstein, G. Herman, G. D. Yancopoulos, D. M. Weinreich, Covid-19 Phase 3 Prevention Trial Team, Subcutaneous REGEN-COV Antibody Combination to Prevent Covid-19. *N. Engl. J. Med.* **385**, 1184–1195 (2021).

71. A. Chevalier, D.-A. Silva, G. J. Rocklin, D. R. Hicks, R. Vergara, P. Murapa, S. M. Bernard, L. Zhang, K.-H. Lam, G. Yao, C. D. Bahl, S.-I. Miyashita, I. Goreshnik, J. T. Fuller, M. T. Koday, C. M. Jenkins, T. Colvin, L. Carter, A. Bohn, C. M. Bryan, D. A. Fernández-Velasco, L. Stewart, M. Dong, X. Huang, R. Jin, I. A. Wilson, D. H. Fuller, D.

Baker, Massively parallel de novo protein design for targeted therapeutics. *Nature* **550**, 74–79 (2017).

72. A. Quijano-Rubio, H.-W. Yeh, J. Park, H. Lee, R. A. Langan, S. E. Boyken, M. J. Lajoie, L. Cao, C. M. Chow, M. C. Miranda, J. Wi, H. J. Hong, L. Stewart, B.-H. Oh, D. Baker, De novo design of modular and tunable protein biosensors. *Nature* **591**, 482–487 (2021).

73. S. K. Elledge, X. X. Zhou, J. R. Byrnes, A. J. Martinko, I. Lui, K. Pance, S. A. Lim, J. E. Glasgow, A. A. Glasgow, K. Turcios, N. S. Iyer, L. Torres, M. J. Peluso, T. J. Henrich, T. T. Wang, C. M. Tato, K. K. Leung, B. Greenhouse, J. A. Wells, Engineering luminescent biosensors for point-of-care SARS-CoV-2 antibody detection. *Nat. Biotechnol.* **39**, 928–935 (2021).

74. K. H. D. Crawford, R. Eguia, A. S. Dingens, A. N. Loes, K. D. Malone, C. R. Wolf, H. Y. Chu, M. A. Tortorici, D. Veesler, M. Murphy, D. Pettie, N. P. King, A. B. Balazs, J. D. Bloom, Protocol and Reagents for Pseudotyping Lentiviral Particles with SARS-CoV-2 Spike Protein for Neutralization Assays. *Viruses* **12**, 513 (2020).

75. A. O. Hassan, S. Shrihari, M. J. Gorman, B. Ying, D. Yaun, S. Raju, R. E. Chen, I. P. Dmitriev, E. Kashentseva, L. J. Adams, C. Mann, M. E. Davis-Gardner, M. S. Suthar, P.-Y. Shi, E. O. Saphire, D. H. Fremont, D. T. Curiel, G. Alter, M. S. Diamond, An intranasal vaccine durably protects against SARS-CoV-2 variants in mice. *Cell Rep.* **36**, 109452 (2021).

76. J. B. Case, P. W. Rothlauf, R. E. Chen, Z. Liu, H. Zhao, A. S. Kim, L.-M. Bloyet, Q. Zeng, S. Tahan, L. Droit, M. X. G. Ilagan, M. A. Tartell, G. Amarasinghe, J. P. Henderson, S. Miersch, M. Ustav, S. Sidhu, H. W. Virgin, D. Wang, S. Ding, D. Corti, E. S. Theel, D. H. Fremont, M. S. Diamond, S. P. J. Whelan, Neutralizing Antibody and Soluble ACE2 Inhibition of a Replication-Competent VSV-SARS-CoV-2 and a Clinical Isolate of SARS-CoV-2. *Cell Host Microbe* **28**, 475-485.e5 (2020).

77. S. J. Fleishman, A. Leaver-Fay, J. E. Corn, E.-M. Strauch, S. D. Khare, N. Koga, J. Ashworth, P. Murphy, F. Richter, G. Lemmon, J. Meiler, D. Baker, RosettaScripts: a scripting language interface to the Rosetta macromolecular modeling suite. *PLoS One* **6**, e20161 (2011).

78. Y. Hsia, R. Mout, W. Sheffler, N. I. Edman, I. Vulovic, Y.-J. Park, R. L. Redler, M. J. Bick, A. K. Bera, A. Courbet, A. Kang, T. J. Brunette, U. Nattermann, E. Tsai, A. Saleem, C. M. Chow, D. Ekiert, G. Bhabha, D. Veesler, D. Baker, Design of multi-scale protein complexes by hierarchical building block fusion. *Nat. Commun.* **12**, 2294 (2021).

79. P.-S. Huang, Y.-E. A. Ban, F. Richter, I. Andre, R. Vernon, W. R. Schief, D. Baker, RosettaRemodel: a generalized framework for flexible backbone protein design. *PLoS One* **6**, e24109 (2011).

80. Y.-R. Lin, N. Koga, R. Tatsumi-Koga, G. Liu, A. F. Clouser, G. T. Montelione, D. Baker, Control over overall shape and size in de novo designed proteins. *Proc. Nat. Acad. Sci. U. S. A.* **112**, E5478–E5485 (2015).

81. G. J. Rocklin, T. M. Chidyausiku, I. Goreshnik, A. Ford, S. Houliston, A. Lemak, L. Carter, R. Ravichandran, V. K. Mulligan, A. Chevalier, C. H. Arrowsmith, D. Baker, Global analysis of protein folding using massively parallel design, synthesis, and testing. *Science* **357**, 168–175 (2017).

82. B. Dang, H. Wu, V. K. Mulligan, M. Mravic, Y. Wu, T. Lemmin, A. Ford, D.-A. Silva, D. Baker, W. F. DeGrado, De novo design of covalently constrained mesosize protein scaffolds with unique tertiary structures. *Proc. Nat. Acad. Sci. U. S. A.* **114**, 10852–10857 (2017).

83. F. Khatib, S. Cooper, M. D. Tyka, K. Xu, I. Makedon, Z. Popovic, D. Baker, F. Players, Algorithm discovery by protein folding game players. *Proc. Natl. Acad. Sci. U. S. A.* **108**, 18949–18953 (2011).

84. A. J. Greaney, T. N. Starr, P. Gilchuk, S. J. Zost, E. Binshtein, A. N. Loes, S. K. Hilton, J. Huddleston, R. Eguia, K. H. D. Crawford, A. S. Dingens, R. S. Nargi, R. E. Sutton, N. Suryadevara, P. W. Rothlauf, Z. Liu, S. P. J. Whelan, R. H. Carnahan, J. E. Crowe Jr, J. D. Bloom, Complete Mapping of Mutations to the SARS-CoV-2 Spike Receptor-Binding Domain that Escape Antibody Recognition. *Cell Host Microbe* **29**, 44-57.e9 (2021).

85. T. N. Starr, A. J. Greaney, A. S. Dingens, J. D. Bloom, Complete map of SARS-CoV-2 RBD mutations that escape the monoclonal antibody LY-CoV555 and its cocktail with LY-CoV016. *Cell Rep Med* **2**, 100255 (2021).

86. B. A. Rabe, C. Cepko, A Simple Enhancement for Gibson Isothermal Assembly. *bioRxiv* , 2020.06.14.150979 (2020).

87. D. G. Gibson, L. Young, R.-Y. Chuang, J. C. Venter, C. A. Hutchison 3rd, H. O. Smith, Enzymatic assembly of DNA molecules up to several hundred kilobases. *Nat. Methods* **6**, 343–345 (2009).

88. T. Ojima-Kato, S. Nagai, H. Nakano, Ecobody technology: rapid monoclonal antibody screening method from single B cells using cell-free protein synthesis for antigen-binding fragment formation. *Sci. Rep.* **7**, 13979 (2017).

89. Z. Chen, R. D. Kibler, A. Hunt, F. Busch, J. Pearl, M. Jia, Z. L. VanAernum, B. I. M. Wicky, G. Dods, H. Liao, M. S. Wilken, C. Ciarlo, S. Green, H. El-Samad, J. Stamatoyannopoulos, V. H. Wysocki, M. C. Jewett, S. E. Boyken, D. Baker, De novo design of protein logic gates. *Science* **368**, 78–84 (2020).

90. P. E. Daddona, H. M. Davis, W. E. Fogler, T. L. Klug, N. C. Ledonne, J. S. Mckinney, Method of removing endotoxin contaminants. *Patent* , WO1989003885A1 (1989).

91. J. Pallesen, N. Wang, K. S. Corbett, D. Wrapp, R. N. Kirchdoerfer, H. L. Turner, C. A. Cottrell, M. M. Becker, L. Wang, W. Shi, W. P. Kong, E. L. Andres, A. N. Kettenbach, M. R. Denison, J. D. Chappell, B. S. Graham, A. B. Ward, J. S. McLellan, Immunogenicity and structures of a rationally designed prefusion MERS-CoV spike antigen. *Proc. Natl. Acad. Sci. U. S. A.* **114**, E7348–E7357 (2017).

92. R. N. Kirchdoerfer, N. Wang, J. Pallesen, D. Wrapp, H. L. Turner, C. A. Cottrell, K. S. Corbett, B. S. Graham, J. S. McLellan, A. B. Ward, Stabilized coronavirus spikes are resistant to conformational changes induced by receptor recognition or proteolysis. *Sci. Rep.* **8**, 15701 (2018).

93. C. J. Russo, L. A. Passmore, Electron microscopy: Ultrastable gold substrates for electron cryomicroscopy. *Science* **346**, 1377–1380 (2014).

94. C. Suloway, J. Pulokas, D. Fellmann, A. Cheng, F. Guerra, J. Quispe, S. Stagg, C. S. Potter, B. Carragher, Automated molecular microscopy: the new Leginon system. *J. Struct. Biol.* **151**, 41–60 (2005).

95. D. Tegunov, P. Cramer, Real-time cryo-electron microscopy data preprocessing with Warp. *Nat. Methods* **16**, 1146–1152 (2019).

96. A. Punjani, J. L. Rubinstein, D. J. Fleet, M. A. Brubaker, cryoSPARC: algorithms for rapid unsupervised cryo-EM structure determination. *Nat. Methods* **14**, 290–296 (2017).

97. J. Zivanov, T. Nakane, B. O. Forsberg, D. Kimanius, W. J. Hagen, E. Lindahl, S. H. Scheres, New tools for automated high-resolution cryo-EM structure determination in RELION-3. *Elife* **7**, e42166 (2018).

98. A. Punjani, H. Zhang, D. J. Fleet, Non-uniform refinement: adaptive regularization improves single-particle cryo-EM reconstruction. *Nat. Methods* **17**, 1214–1221 (2020).

99. J. Zivanov, T. Nakane, S. H. W. Scheres, A Bayesian approach to beam-induced motion correction in cryo-EM single-particle analysis. *IUCrJ* **6**, 5–17 (2019).

100. S. Chen, G. McMullan, A. R. Faruqi, G. N. Murshudov, J. M. Short, S. H. Scheres, R. Henderson, High-resolution noise substitution to measure overfitting and validate resolution in 3D structure determination by single particle electron cryomicroscopy. *Ultramicroscopy* **135**, 24–35 (2013).

101. P. B. Rosenthal, R. Henderson, Optimal determination of particle orientation, absolute hand, and contrast loss in single-particle electron cryomicroscopy. *J. Mol. Biol.* **333**, 721–745 (2003).

102. E. F. Pettersen, T. D. Goddard, C. C. Huang, E. C. Meng, G. S. Couch, T. I. Croll, J. H. Morris, T. E. Ferrin, UCSF ChimeraX: Structure visualization for researchers, educators, and developers. *Protein Sci.* **30**, 70–82 (2021).

103. P. Emsley, B. Lohkamp, W. G. Scott, K. Cowtan, Features and development of Coot. *Acta Crystallogr. D Biol. Crystallogr.* **66**, 486–501 (2010).

104. R. Y.-R. Wang, Y. Song, B. A. Barad, Y. Cheng, J. S. Fraser, F. DiMaio, Automated structure refinement of macromolecular assemblies from cryo-EM maps using Rosetta. *Elife* **5**, e17219 (2016).

105. B. Frenz, A. C. Walls, E. H. Egelman, D. Veesler, F. DiMaio, RosettaES: a sampling strategy enabling automated interpretation of difficult cryo-EM maps. *Nat. Methods* **14**, 797–800 (2017).

106. V. M. Corman, V. C. Haage, T. Bleicker, M. L. Schmidt, B. Mühlemann, M. Zuchowski, W. K. Jo, P. Tscheak, E. Möncke-Buchner, M. A. Müller, A. Krumbholz, J. F. Drexler, C. Drosten, Comparison of seven commercial SARS-CoV-2 rapid point-of-care antigen tests: a single-centre laboratory evaluation study. *Lancet Microbe* **2**, e311–e319 (2021).

107. Y. M. Bar-On, A. Flamholz, R. Phillips, R. Milo, SARS-CoV-2 (COVID-19) by the numbers. *Elife* **9**, 1–15 (2020).

108. P. S. Katsamba, I. Navratilova, M. Calderon-Cacia, L. Fan, K. Thornton, M. Zhu, T. V. Bos, C. Forte, D. Friend, I. Laird-Offringa, G. Tavares, J. Whatley, E. Shi, A. Widom, K. C. Lindquist, S. Klakamp, A. Drake, D. Bohmann, M. Roell, L. Rose, J. Dorocke, B. Roth, B. Luginbühl, D. G. Myszka, Kinetic analysis of a high-affinity antibody/antigen interaction performed by multiple Biacore users. *Anal. Biochem.* **352**, 208–221 (2006).

109. A. E. Firth, W. M. Patrick, GLUE-IT and PEDEL-AA: new programmes for analyzing protein diversity in randomized libraries. *Nucleic Acids Res.* **36**, 281–285 (2008).

110. J. B. Case, A. L. Bailey, A. S. Kim, R. E. Chen, M. S. Diamond, Growth, detection, quantification, and inactivation of SARS-CoV-2. *Virology* **548**, 39–48 (2020).

111. F. Madeira, Y. M. Park, J. Lee, N. Buso, T. Gur, N. Madhusoodanan, P. Basutkar, A. R. N. Tivey, S. C. Potter, R. D. Finn, R. Lopez, The EMBL-EBI search and sequence analysis tools APIs in 2019. *Nucleic Acids Res.* **47**, W636–W641 (2019).

112. S. E. Boyken, Z. Chen, B. Groves, R. A. Langan, G. Oberdorfer, A. Ford, J. M. Gilmore, C. Xu, F. DiMaio, J. H. Pereira, B. Sankaran, G. Seelig, P. H. Zwart, D. Baker, De novo design of protein homo-oligomers with modular hydrogen-bond network-mediated specificity. *Science* **352**, 680–687 (2016).

113. G. Ueda, A. Antanasijevic, J. A. Fallas, W. Sheffler, J. Copps, D. Ellis, G. B. Hutchinson, A. Moyer, A. Yasmeen, Y. Tsybovsky, Y.-J. Park, M. J. Bick, B. Sankaran, R. A. Gillespie, P. J. Brouwer, P. H. Zwart, D. Veesler, M. Kanekiyo, B. S. Graham, R. W. Sanders, J. P. Moore, P. J. Klasse, A. B. Ward, N. P. King, D. Baker, Tailored design of protein nanoparticle scaffolds for multivalent presentation of viral glycoprotein antigens. *Elife* **9**, e57659 (2020).

114. S. E. Boyken, M. A. Benhaim, F. Busch, M. Jia, M. J. Bick, H. Choi, J. C. Klima, Z. Chen, C. Walkey, A. Mileant, A. Sahasrabuddhe, K. Y. Wei, E. A. Hodge, S. Byron, A. Quijano-Rubio, B. Sankaran, N. P. King, J. Lippincott-Schwartz, V. H. Wysocki, K. K. Lee, D. Baker, De novo design of tunable, pH-driven conformational changes. *Science* **364**, 658–664 (2019).

115. P. B. Harbury, P. S. Kim, T. Alber, Crystal structure of an isoleucine-zipper trimer. *Nature* **371**, 80–83 (1994).

116. A. R. Thomson, C. W. Wood, A. J. Burton, G. J. Bartlett, R. B. Sessions, R. L. Brady, D. N. Woolfson, Computational design of water-soluble α-helical barrels. *Science* **346**, 485–488 (2014).
